# Synthesis of Janus All-*Cis* Tetrafluorocyclohexanes Carrying 1,4-Diether Motifs

**DOI:** 10.1021/acs.joc.4c02345

**Published:** 2024-11-27

**Authors:** Thomas J. Poskin, Bruno A. Piscelli, Aidan P. McKay, David B. Cordes, Yuto Eguchi, Shigeyuki Yamada, Rodrigo A. Cormanich, David O’Hagan

**Affiliations:** † School of Chemistry, University of St Andrews, North Haugh, St Andrews KY16 9ST, U.K.; ‡ Instituto de Química, Departamento de Química Orgânica, Universidade Estadual de Campinas, P.O. Box 6154, Campinas, São Paulo 13083-970, Brazil; § Faculty of Molecular Chemistry and Engineering, Kyoto Institute of Technology, Matsugasaki, Sakyo-ku, Kyoto 606-8585, Japan

## Abstract

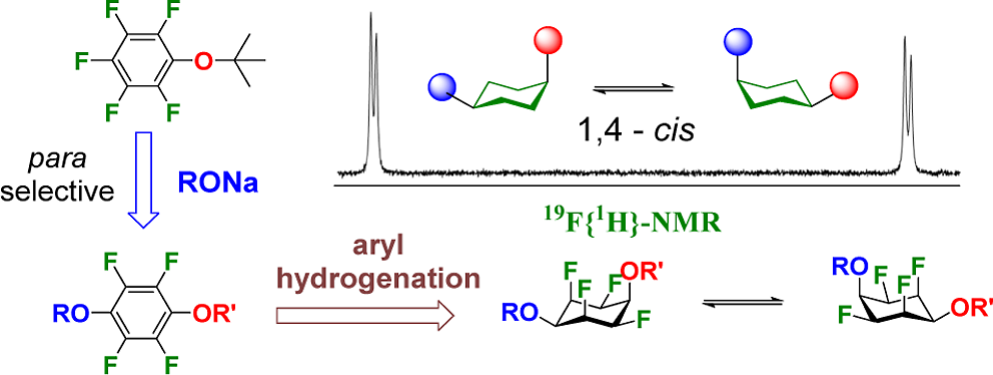

Nucleophilic aromatic substitutions (S_N_Ar) of alkoxides on pentafluoroaryl ethers are explored as a first step in a synthesis sequence to generate all-*cis* 2,3,5,6-tetrafluorocyclohexyl-1,4-dialkyl ethers **1**. The S_N_Ar reaction was explored both experimentally and theoretically to rationalize *ortho*/*para*/*meta* selectivities. *tert*-Butyl deprotection of products followed by phenol alkylations introduces versatility to the synthesis. The final Rh(CAAC) **3** catalyzed aryl hydrogenation step of intermediate tetrafluoroaryl-1,4-diethers generated cyclohexane products **1**. This chemistry introduces a new class of Janus fluorocyclohexane derivatives with ether substituents placed 1,4- to each other.

## Introduction

**Figure 1:**
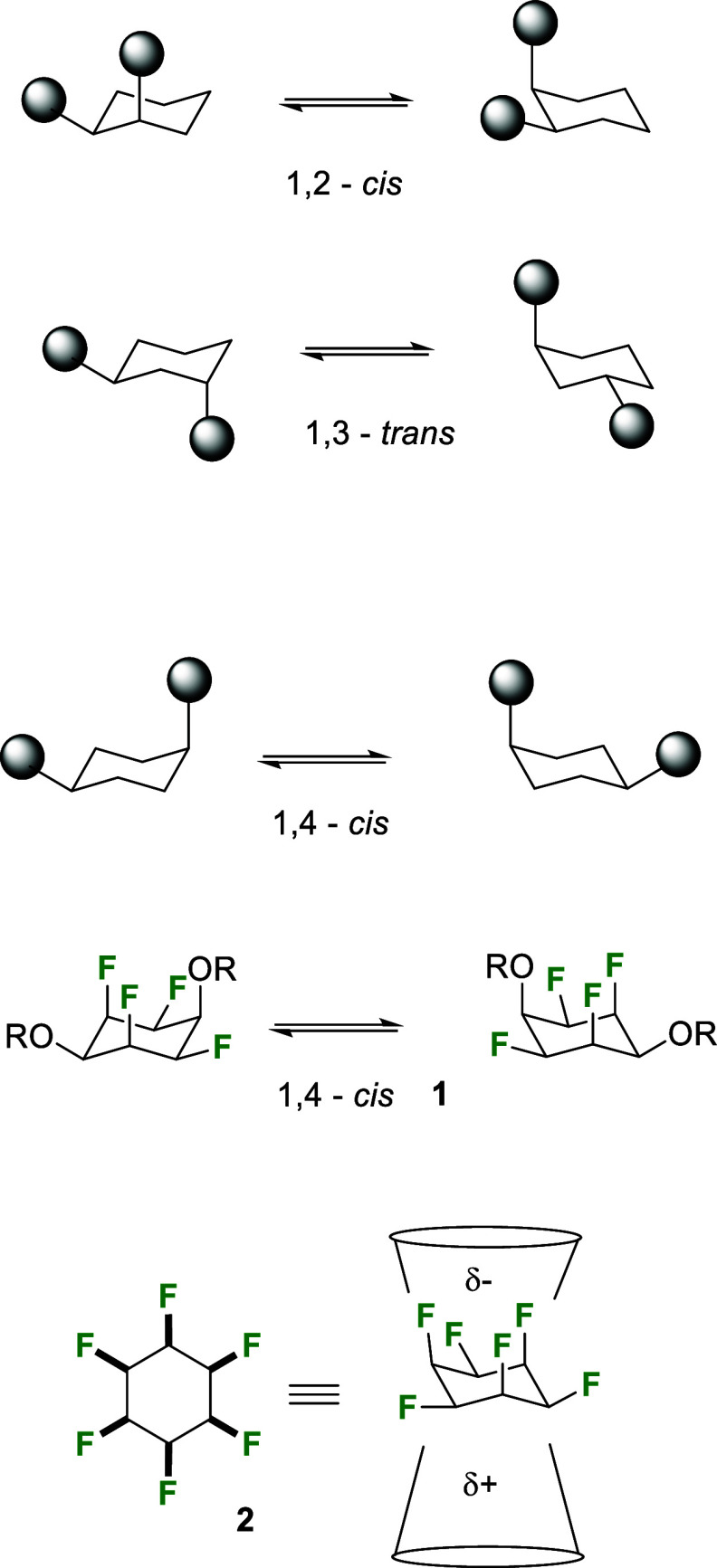
“Thermodynamically disfavored” cyclohexanes and all-*cis*-1,2,3,4,5,6-hexafluorocyclohexane **2**.

There has been a recent discussion regarding the synthesis and properties of “thermodynamically disfavored” cyclohexanes,^1^ where there is an *ax*/*eq* or *eq*/*ax* conformational ambiguity, such as is found in *cis*-1,2, *trans*-1,3, and *cis*-1,4 cyclohexanes, as illustrated in Figure 1. Chemistry to access such substitutions will generally tend toward thermodynamically preferred diequatorial isomers, and thus, specific methods have to be devised to achieve these thermodynamically disfavored *cis*-configurations. The relatively low representation of such compounds across the large organic molecule demographic runs counter to a wider consensus that molecules with an increased dimensionality offer new prospects for uncovering innovative properties from materials^2^ to bioactives.^3^

In this paper, we explore the preparation of *cis*-1,4 cyclohexyl ethers **1**, where the cyclohexane ring also has four fluorine atoms arranged around the ring and with an all-*cis* configuration. This is an extension of our current interest in facially polarized Janus cyclohexanes.^4^ All-*cis*-1,2,3,4,5,6-hexafluorocyclohexane **2** has attracted some interest due to its high polarity, particularly as it is a cyclohexane, which as a class is generally found to be hydrophobic.^5^ In the interconverting chair conformations of this cyclohexane, there are always triaxial C–F bonds pointing in the same direction, which impart a strong molecular dipole. It is the coalignment of the axial C–F bonds, which contribute most significantly to its the molecular polarity.^6^

**Figure 2:**
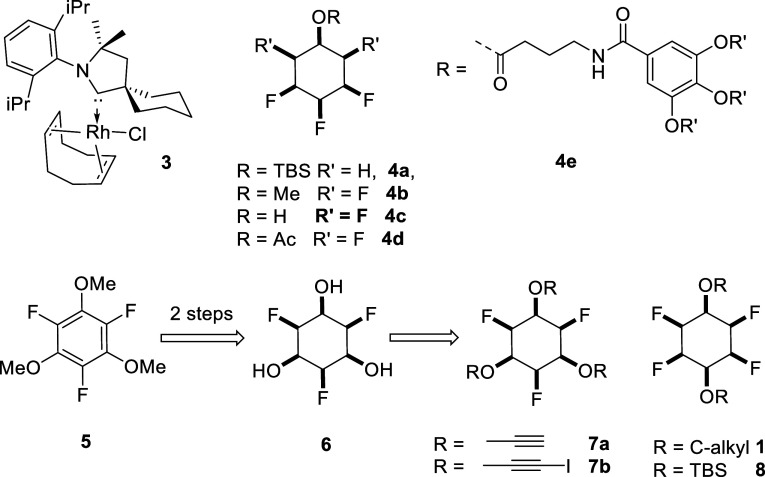
CAAC catalyst **3** and Janus ether substituted cyclohexanes.^8−10^

These systems have been termed Janus cyclohexanes^7^ as they have two faces (electropositive-H and electronegative-F face). The first preparation of **2** in our laboratory^5^ from inositol was significantly improved by the Glorius laboratory,^8^ who developed a direct aryl hydrogenation of hexafluorobenzene. The method involves high pressure hydrogenations (∼50 bar H_2_) using a Rh catalyst, which was applied to aryl hydrogenations by Zeng et al.^9^ The catalyst **3** shown in Figure 2 contains the strongly electron-donating cyclic alkyl amino carbene ligand (CAAC) and reactions are carried out in low polarity solvents. This combination suppressed competing dehydrofluorination reactions, generating a series of Janus cyclohexanes with good efficiency. For the preparation of ethers, Glorius et al.^8c^ exemplified the aryl hydrogenation of fluorophenyl silyl (TBS) ethers to the corresponding all-*cis* pentafluorocyclohexyl ethers, such as **4a**. Subsequently, von Delius’s laboratory^10^ demonstrated aryl hydrogenation to methyl ether **4b** from the pentafluoroanisole precursor. This methyl ether could be converted to alcohol **4c** and derivatized to acetate **4c** and more elaborate esters, such as **4e**, which acted as monomers displaying interesting dynamic behavior due to self-assembly equilibria between the Janus rings. Most recently,^11^ von Delius’s laboratory has extended to examples of all-*cis* 1,3,5-triethers **7a** and **7b** (via triol **6**), which have been prepared by aryl hydrogenations of trifluorotrimethoxybenzene **5** followed by demethylation. The three ether substituents in **7a** and **7b** adopt a thermodynamically favorable triequatorial conformation, and as a consequence, the three C–F bonds are triaxial. This arrangement is attractive as it offers trisubstituted cyclohexanes with a large dipole due to the oriented C–F bonds.

We address here the synthesis of all-*cis* tetrafluoro disubstituted cyclohexanes **1**, where alkyl ether substituents are *cis*-1,4 to each other. They are a class of Janus cyclohexanes where oxygens replace the 1,4-fluorines; however, there will always be an axial and an equatorial C-OR bond in interconverting chair conformations rendering these “thermodynamically disfavored”. The only example of a 1,4-ether that we are aware of is *bis* silyl ether **8**, which was reported in the original Glorius paper.^8^ The new class of Janus 1,4-dialkyl ethers may offer some advantages in that they will have more complex 3D arrangements and will be more dynamic in supramolecular assemblies. The route to these 1,4-cyclohexyl diethers exploits a *para* selective S_N_Ar reaction on *t*ButO-pentafluorophenol ether **10** and then aryl hydrogenations offer access to molecules of class **1**.

## Results and Discussion

**Scheme 1:**
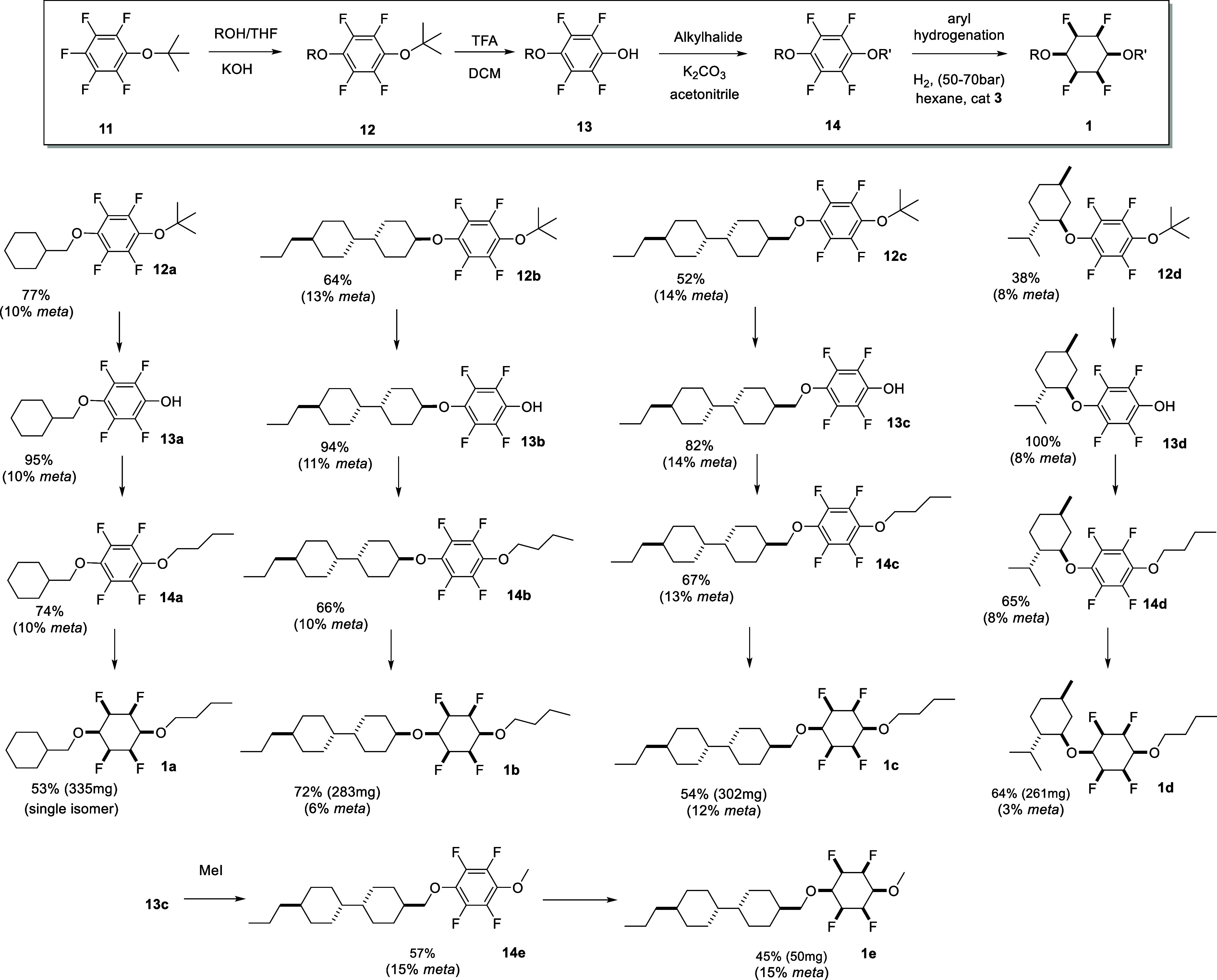
Synthesis Routes to All-*Cis* Tetrafluorocyclohexyl Diethers **1a–1e** from *t*ButO Ether **11**

A general route to 1,4-disubstituted tetrafluorocyclohexyl ether **1** is outlined in Scheme 1. For S_N_Ar reactions on pentafluoroaryl rings, a regiochemical preference for *para* over *meta* attack by nucleophiles is generally observed; although the bias is dependent on the indigenous substituent.

For example, S_N_Ar reactions on C_6_F_5_X systems with electron-withdrawing groups (–NO_2_, –CN) are very highly selective for *para* products^12^ as is a –Ph substituent^13^ or even an unsubstituted –H substituent.^14^ For pentafluoroaryl ethers such as X = OMe, the *para* over the *meta* directing effect is much more modest relative to –H^14^ and –Ph,^13^ due to the mesomeric donor ability of the methoxyl group. In order to maximize the regiochemical preference in favor of *para* addition for an alkoxide nucleophile to a pentafluoroaryl ether, an exploration of progressively larger indigenous ether substituents (–OMe **9**, –O*i*Pr **10**, and –O*t*But **11**) was conducted and with progressively larger alkoxide nucleophiles to enhance both steric and stereoelectronic factors. Reactions were conducted in THF with **9**, **10**, and **11** reacting with sodium methoxide (OMe^–^), *iso*-propoxide (*i*PrO^–^) and *tert*-butoxide (*t*ButO^–^) as nucleophiles. The *ortho*/*para*/*meta* product ratios are represented in Table 1. It emerged that the *meta*/*para* ratio is the lowest in the reactions conducted on the *t*ButO-aryl ether **11** and with *t*ButO^–^ as a nucleophile.

**Table 1:**
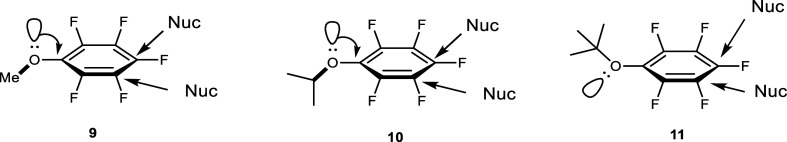
*Ortho*/*Para*/*Meta* Ratios of Products from Reactions of **9–11** with Alkoxide Nucleophiles^a^

nucleophile	**9** (MeO)			**10** (*i*PrO)			**11** (*t*ButO)		
	*ortho*	*para*	*meta*	*ortho*	*para*	*meta*	*ortho*	*para*	*meta*
MeONa	0.55	1.27	1.0	0.0	1.8	1.0	0.0	3.7	1.0
*i*PrONa	0.49	1.34	1.0	0.0	1.7	1.0	0.0	4.3	1.0
*t*ButONa	0.28	1.81	1.0	0.0	2.4	1.0	0.0	7.1	1.0

a Conditions: **9**, **10** or **11** (1.0 equiv), alcohol (0.5 equiv), NaH (0.75 equiv), THF (3 mL), 2.5 h, 50 °C. (Ratios are an averages of triplicates).

It is not clear where the origin of this selectivity arises. In aryl ethers (anisoles), the MeO^–^ substituent tends to adopt a planar orientation with respect to the aromatic ring, maximizing oxygen lone pair conjugation into the ring whereas the *t*ButO^–^ group adopts a perpendicular conformation, which will reduce the donor potential of the oxygen lone pairs.^15^ However, the *ortho* fluorines in **9** also direct the –OMe to a more favored perpendicular conformation,^16^ so stereoelectronically the MeO- and *t*ButO-ethers are more equivalent in this perfluorinated series. In order to explore these effects further, reaction profiles with an alkoxide nucleophile were conducted computationally for MeO- ether **9** and *t*ButO-ether **11**. In each case, reaction profiles were modeled for *para* and *meta* trajectories and with MeO^–^ and *t*ButO^–^ as nucleophiles.

**Figure 3:**
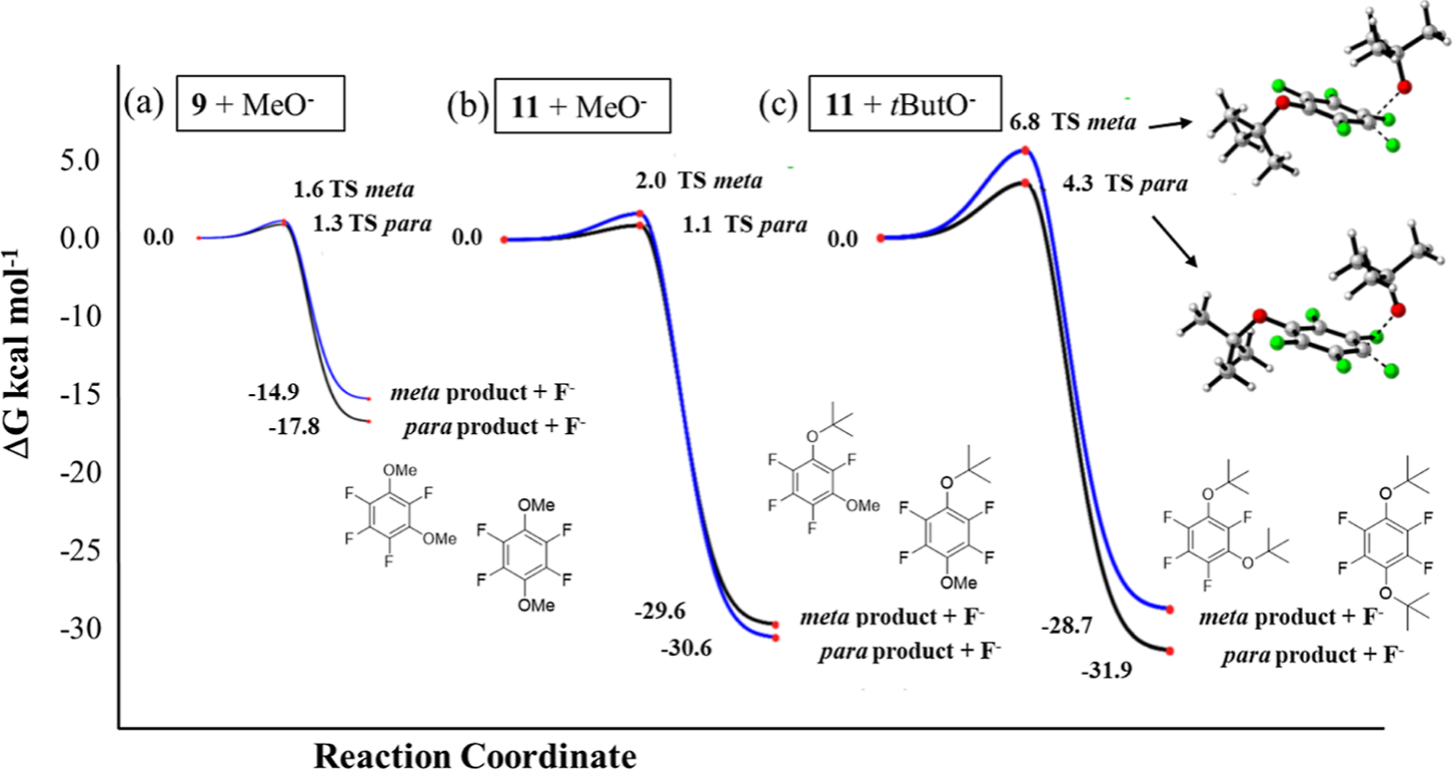
Reaction profiles for S_N_Ar reactions of (a) **9** with OMe^–^; (b) **11** with OMe^–^; and (c) **11** with *t*ButO^–^; each attacking either at the *meta* or *para* positions calculated at the M06-2X/6-311++G** level. The *para* reaction course is kinetically favored in each case.

**Figure 4:**
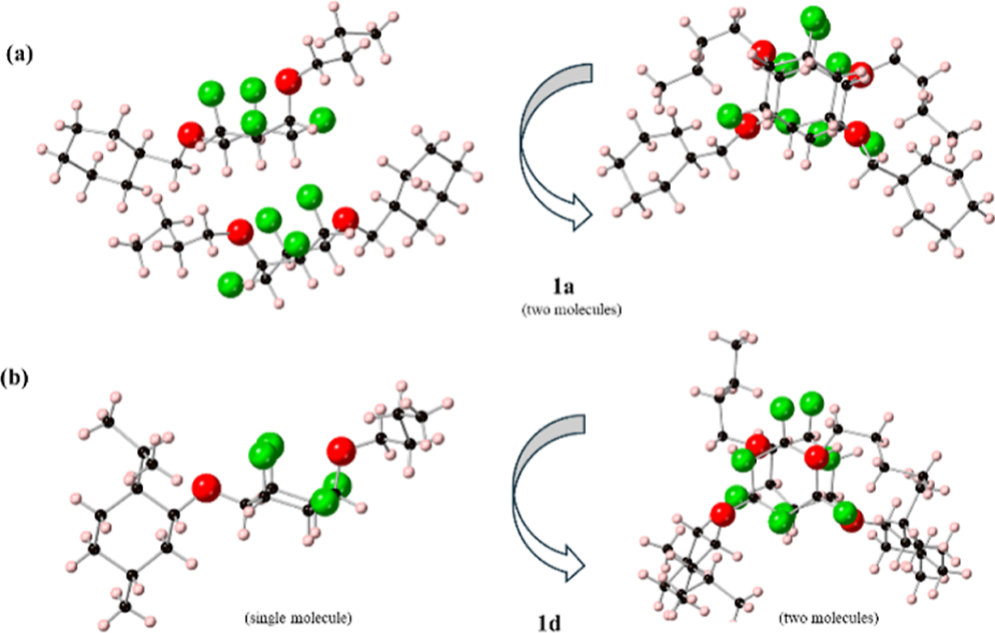
X-ray structures of 1,4-diethers (a) **1a** and (b) **1d** viewed from two trajectories.

It proved challenging to locate transition states (TSs), but they could be found employing a developing version of Autobench^17^ for energy reaction barriers. Considering the methoxyl ether **9** as the substrate and with MeO^–^ as the nucleophile, the energies of the TSs are very similar and only a little (1.3 and 1.6 kcal mol^–1^) above the ground state energy of **9**, as illustrated in Figure 3a. In each case, the reaction is highly exergonic (Δ*G*^0^ = −14.9 and −17.8 kcal mol^–1^) with a high barrier to reversibility and thus the *meta*/*para* ratio appears to be determined kinetically with the *meta* TS a little higher (ΔΔ*G*^‡^ = 0.8 kcal mol^–1^) than that of the *para* TS (Figure 4). When **11** is the substrate and with MeO^–^ again as the nucleophile, the reaction follows a trend similar to that of **9**, with a ΔΔ*G*^‡^ = 0.9 kcal mol^–1^, but the reaction is very much more exergonic for the *meta*/*para* products (Δ*G*^0^ = −29.6 and −30.6 kcal mol^–1^), as illustrated in Figure 3b. On the other hand, when *t*ButO^–^ is the nucleophile combining with tButO ether **11**, there is an increase in the reaction energy barriers to ΔΔ*G*^‡^ = 2.5 kcal mol^–1^ favoring the *para* TS, and the reaction is also high exergonic, as illustrated in Figure 3c. In all of these reactions, intermediate Meisenheimer complexes could not be located computationally, and these reactions appear to progress through a concerted S_N_Ar substitution process. Concerted processes have already been proposed for S_N_Ar of perfluoroaromatics,^18,19^ and this general mechanism for S_N_Ar reactions was evaluated by Jacobsen^20^ and has been discussed more widely.^21^

With the knowledge that the *para*/*meta* ratio is most influenced by an incipient *t*ButO ether, **11** emerged as an attractive starting material in order to most effectively influence the *para*/*meta* regiochemistry. S_N_Ar reactions generated the *t*ButO ether products **12**, predominantly as the *para* products, with lesser levels of *meta* isomers (≤15%), as illustrated in Scheme 1. The ethers were then conveniently cleaved to aryl ether phenols **13** by treatment with trifluoroacetic acid. Subsequent alkylation of phenols **13**, in this case with 1-bromobutane or methyl iodide-generated diaryl ethers **14**.

The resultant ethers **14** were then subject to aryl hydrogenations (H_2_ 50 bar, cat **3**)^8^ to generate a range of all-*cis* tetrafluoro-substituted cyclohexyl diethers **1**. The approach could reasonably be applied quite widely although the reactions explored here were conducted with well-known liquid crystal motifs as the targets.^22^

In the case of **1b–e**, differential scanning calorimetry and polarized optical microscopy analysis showed significant levels of crystalline polymorphism in each case, although no LC phases were observed during both the heating and cooling processes (see Figures S1–S4). In all Janus *cis*-tetrafluorocyclohexane **1b–e**, the alkoxy moieties introduced at the 1,4-positions occupy axial and equatorial positions, forming a bent structure, which appears to prevent LC phase formation.

1,4-Diether products **1a–e** were solids and single crystals of **1a** and **1d** were obtained and submitted for X-ray structure analysis. The resultant structures are illustrated in Figure 4. In each case, the structures adopt the expected chair conformations in the solid state and, consequently, the 1,4-substitution dictates an axial and an equatorial ether bond in each cyclohexane. In both cases, it is the *n*-butyl ether that adopts the axial orientation. One feature of both structures is that the cyclohexane rings do not stack directly above each other; instead, they rotate such that the C–O bonds eclipse C–F bonds in the ring stacks. Essentially, there is less order in ring stacking relative to compounds where the substituents are all equatorial.^23^

**Figure 5:**
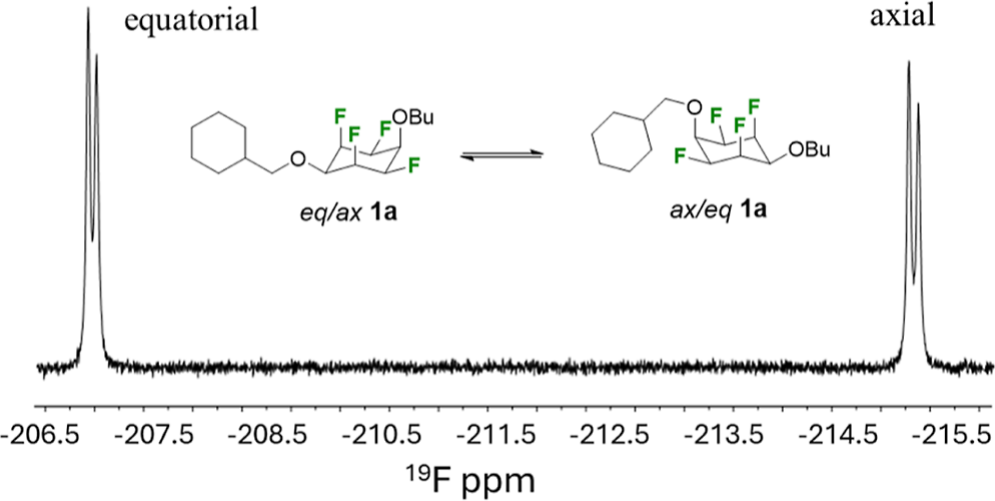
^19^F{^1^H}-NMR of **1a** showing an equilibrium mix of *eq*/*ax***1a** and *ax*/*eq***1a** in solution.

In the solid state, only a single conformer is adopted; however, in solution, ^19^F-[^1^H}NMR analysis indicates that these compounds adopt ∼1:1 mixed ratios of presumably the chair conformers, when the ether substituents are nonequivalent and with these nonequivalent substituents switching between axial and equatorial orientations. This is obvious in the representative NMR spectra of **1a**, as illustrated in Figure 5.

**Figure 6:**
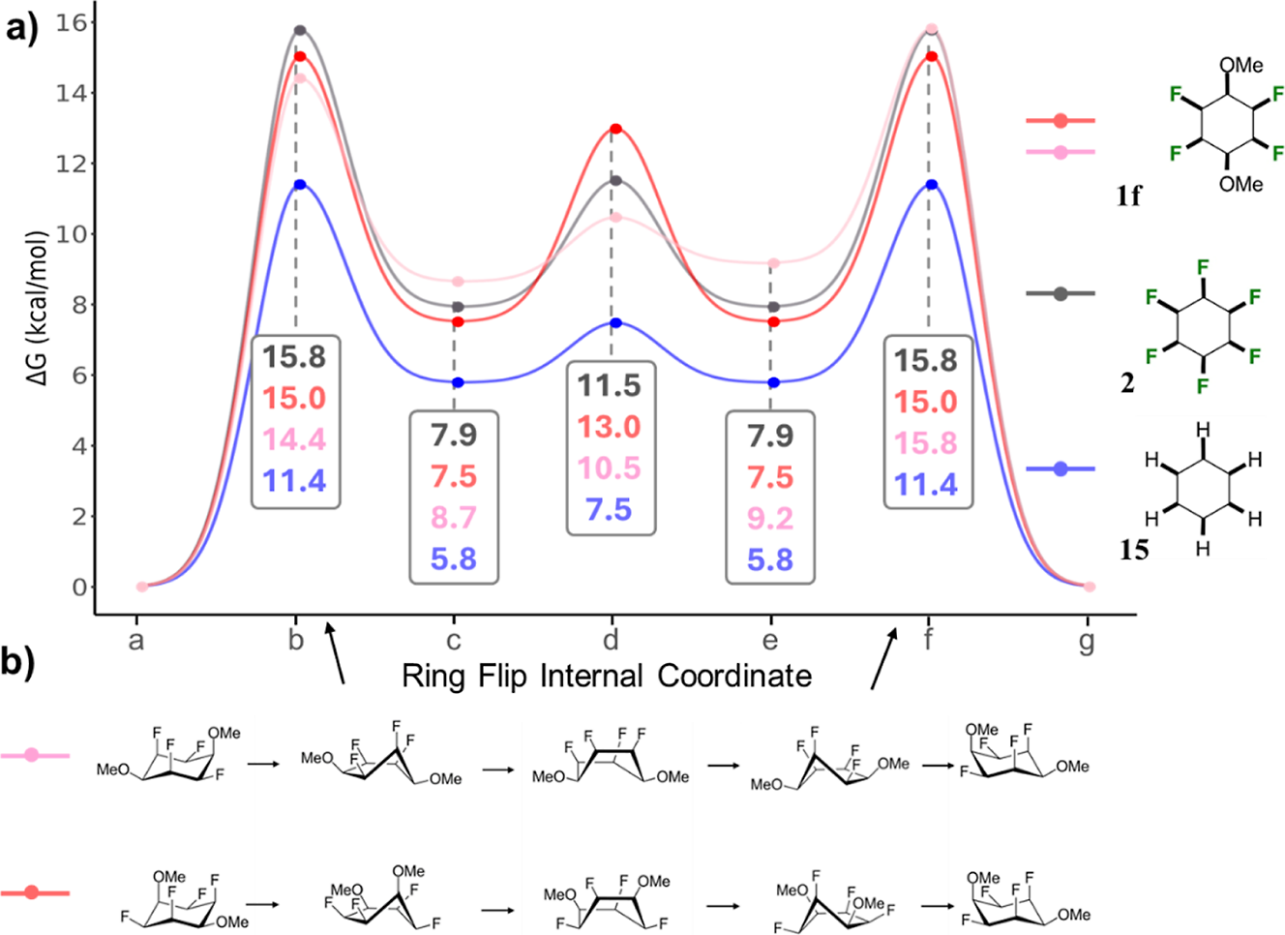
(a) Comparative cyclohexyl interconversion energy profiles for dimethoxy ether **1f**, all-*cis*1,2,3,4,5,6-exafluorocyclohexane **2** and cyclohexane **15** showing the relative energies of intermediates. Energy values in kcal mol^–1^. (b) Schematic of the two interconversion pathways for cyclohexane **1f**.

The pathway and energy barrier to this interconversion was explored theoretically for the symmetric tetrafluoro dimethyl ether **1f** and barriers to interconversion were compared relative to all-*cis*-hexafluorofluorocyclohexane **2** and to cyclohexane **15** itself to act as points of reference. Calculations were carried out at the M06-2X/6-311++G** theory level.^24,25^ The resultant interconversion pathways and energy barriers are illustrated in Figure 6.

The values computed here for the TSs and, also the twisted boat intermediates, are similar to those reported in previous studies for both all-*cis*1,2,3,4,5,6-hexafluorocyclohexane **2**(26) and cyclohexane **15**(27) and, therefore, we assume that the calculated conformational energy profile for **1f** is relatively accurate. It is notable that the highest barrier to interconversion for **1f** at the “half-chair” structure is close to that for **2** at its “half-chair” intermediate, suggesting a similar level of negative electrostatic interaction between the C–O and C–F bonds in **1f**, and C–F and C–F bonds in **2** as the conformational profile progresses through these two intermediates. In fact, the “half-chair” TS in **1f** is 0.8 kcal mol^–1^ lower in energy compared to **2** (15.0 against 15.8 kcal mol^–1^), which is attributed to the formation of OC–H···F stabilizing contacts formed between methoxy groups and fluorine atoms (Figure S8). These half–chair TSs are ∼4.0 kcal mol^–1^ above that for cyclohexane **15**, where only C–H/C–H bond contacts are present. There are two conformational profiles for **1f**, corresponding to two possible symmetrical boat intermediate structures where either two C–O or two C–F bonds occupy the apical positions of the TS structure (see Figure 6b). Ethoxylation at positions 1 and 4 on the fluorinated systems does not significantly affect the ring-flip energy barriers, which are around 15 kcal mol^–1^ and easily surmountable at room temperature. As a result, it is expected that asymmetric compounds **1a–e**, despite showing a single conformer in the solid state, are in a rapid equilibrium between both chair conformers in solution.

In conclusion, we present a synthesis to all-*cis* 2,3,5,6-tetrafluorocyclohexanes **1** carrying 1,4-diether moieties, as a new class of Janus cyclohexanes. These were prepared by S_N_Ar chemistry with various alkoxide nucleophiles on pentafluoroaryl ether **11**. Cleavage of the *t*ButO ethers **12** followed by alkylation of the resultant phenols **13**, allowed a range of 1,4 diethers **14** to be prepared, and these were subjected to aryl hydrogenations to generate cyclohexanes of class **1**. Low levels of the *meta* isomers were evident from the S_N_Ar reaction, a reaction that was investigated computationally. This analysis indicated a maximum energy difference (∼2.5 kcal mol^–1^) between *para* and *meta* TSs, for *t*ButO ether **11** and *t*BuO^–^ as a nucleophile, which is consistent with the experimental outcomes. These “thermodynamically disfavored” compounds form less ordered arrangements in the solid state than previous prepared all-*cis*-fluorocyclohexanes.^23,28^

## Data Availability

The data underlying this study are available in the published article and its Supporting Information.

## Supporting Information Available

Experimental details and analyses of prepared compounds **1a–1e**, copies of ^1^H, ^13^C{^1^H} and ^19^F{^1^H} NMR spectra, X-ray data **1a** and **1d**, details of computation reaction barriers for S_N_Ar reactions on **9** and **11**, and interconversion barriers for cyclohexanes **1f**, **2**, and **15** (PDF)
