# Supplementary material for: Synthesis of Janus All-Cis Tetrafluorocyclohexanes Carrying 1,4-Diether Motifs
Source: J Org Chem. 2024 Nov 27;89(24):18445–51. doi: 10.1021/acs.joc.4c02345 (PMC11667729; doi:10.1021/acs.joc.4c02345)
Supplement: Supplementary file 1 — jo4c02345_si_001.pdf [file jo4c02345_si_001.pdf]

Supplementary Information

Synthesis of Janus all-*cis* tetrafluorocyclohexanes carrying 1,4-diether motifs

Thomas J Poskin,[1] Bruno A. Piscelli,[2] Aidan P. McKay,[1] David B. Cordes,[1] Yuto Eguchi,[3] Shigeyuki Yamada,[3] Rodrigo A. Cormanich[2*] and David O'Hagan[1*]

[1]School of Chemistry, University of St Andrews, North Haugh, St Andrews, KY16 9ST, UK.

E-mail: do1@st-andrews.ac.uk;

[2] Universidade Estadual de Campinas, Instituto de Química, Departamento de Química Orgânica, PO Box 6154–13083-970–Campinas, São Paulo, Brazil.

E-mail: cormanich@unicamp.br

[3]Faculty of Molecular Chemistry and Engineering, Kyoto Institute of Technology, Matsugasaki, Sakyo-ku, Kyoto 606-8585, Japan.

INDEX

1	Analytical Instrumentation	Page S3
2	General procedure for determining S_NAr *ortho:para:meta* ratios with pentafluoro aryl ethers and alkoxide nucleophiles.	Pages S4 - S14
3	Synthesis protocols	Page S15
4	Experimental Section	Pages S16 - S27
5	Images of NMR spectra for synthesized compounds	Pages S28 – S50
6	Differential Scanning Calorimetry (DSC) Profiles	Pages S51 - S54
7	Details of X-ray Crystallography	Pages S55 - S57
8	Details of Theory/Computation Reaction Barrier calculations SNAr	Pages S58 - S62
9	Details of Theory/Computation for cyclohexane interconversions 1a, 2 and 15	Pages S63 - S66

1 Analytical Instrumentation supporting synthesis

All reactions were carried out under an atmosphere of argon in oven-dried glassware. Hydrogenation reactions at elevated pressure were carried out in stainless steel autoclaves using hydrogen gas. Commercially available chemicals were used as received unless otherwise stated.

Analytical thin layer chromatography was performed on pre-coated aluminium plates (Kieselgel 60 F254 silica) and visualisation was achieved using ultraviolet light (254 nm) and/or staining with aqueous $KMnO_4$ solution followed by heating. Flash column chromatography with Kieselgel 60 silica. Automated chromatography was performed on a Biotage Selekt 2 system with a UV/Vis detector using cartridges filled with Kieselgel 60 silica.

Melting points were recorded on an Electrothermal 9100 melting point apparatus and (dec) refers to decomposition.

IR spectra were recorded on a Shimadzu IRAffinity-1 Fourier transform IR spectrophotometer fitted with a Specac Quest ATR accessory (diamond puck). Spectra were recorded of either thin films or solids, with characteristic absorption wavenumbers (ν_{max}) reported in cm^{-1}.

^{1}H, $^{13}C\{^{1}H\}$, and $^{19}F\{^{1}H\}$ NMR spectra were acquired on either a Bruker AVII 400 with a BBFO probe (^{1}H 400 MHz; $^{13}C\{^{1}H\}$ 101 MHz; $^{19}F\{^{1}H\}$ 376 MHz), a Bruker AVIII-HD 500 with a SmartProbe BBFO+ probe (^{1}H 500 MHz, $^{13}C\{^{1}H\}$ 126 MHz, $^{19}F\{^{1}H\}$ 470 MHz) or a Bruker AVIII 500 with a CryoProbe Prodigy BBO probe (^{1}H 500 MHz, $^{13}C\{^{1}H\}$ 126 MHz, $^{19}F\{^{1}H\}$ 470 MHz) in the deuterated solvent stated. All chemical shifts are quoted in parts per million (ppm) relative to the residual solvent peak. All coupling constants, J, are quoted in Hz. Multiplicities are indicated as s (singlet), d (doublet), t (triplet), q (quartet), m (multiplet), and multiples thereof. The abbreviation Ar denotes aromatic and app denotes apparent.

Mass spectrometry (m/z) data were acquired by either electrospray ionisation (ESI), chemical ionisation (CI), electron impact (EI), atmospheric solids analysis probe (ASAP), atmospheric pressure chemical ionization (APCI), matrix-assisted laser desorption/ionization (MALDI), Atmospheric pressure photoionization (APPI), fast atom bombardment (FAB) or nanospray ionisation (NSI).

2. General procedure for determining S_NAr *ortho:para:meta* ratios with pentafluoro aryl ethers and alkoxide nucleophiles.

Typically to a stirred solution of the chosen alcohol (MeOH, isopropanol or tert-butanol) (0.25 mmol, 0.5 eq) in THF (1 mL) was added NaH (60% in paraffin oil, 15 mg, 0.375 mmol, 0.75 eq) under N_2. The resulting mixture was stirred for 20 min at rt. Then a solution of the pentafluoroaryl ether substrate (0.5 mmol, 1 eq) in THF (2 mL) was added and the reaction was heated to 50°C for up to 2.5 h.

The following *ortho : para : meta* ratios were determined by $^{19}F\{^1H\}$-NMR analysis of the product mixture. Data are an average of three experiments for each reaction.

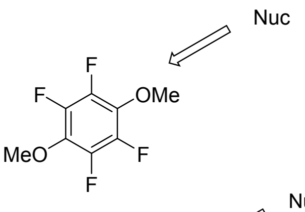

(*ortho* : *para* : *meta* = 0.55 : 1.27 : 1.0).

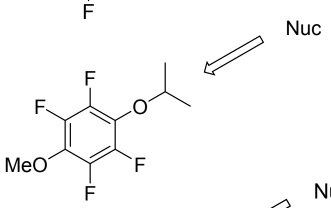

(*ortho* : *para* : *meta* = 0.49 : 1.34 : 1.0)

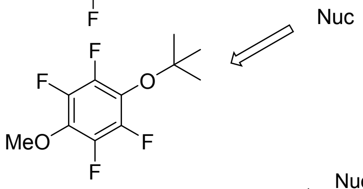

(*ortho* : *para* : *meta* = 0.28 : 1.81 : 1.0).

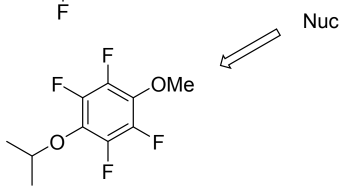

(ortho: *para* : *meta* = 0.0 : 1.8 : 1.0).

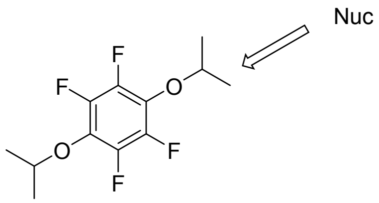

(ortho: *para* : *meta* = 0.0 : 1.7 : 1.0).

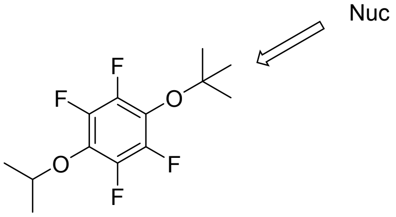

(ortho: *para* : *meta* = 0.0: 2.4 : 1.0).

(ortho: *para* : *meta* = 0.0: 3.7 : 1.0).

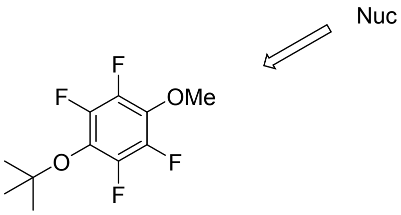

(ortho: *para* : *meta* = 0.0: 4.3: 1.0).

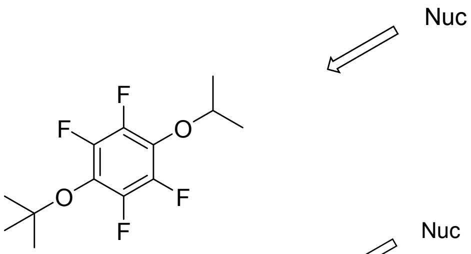

(ortho: *para* : *meta* = 0.0: 7.1 : 1.0).

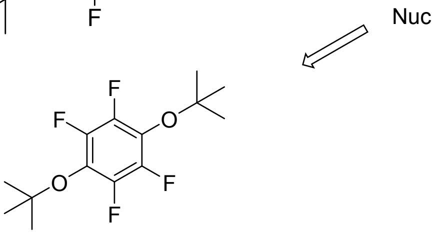

ortho : *para* : *meta* = 0.0 : 3.7 : 1.0.

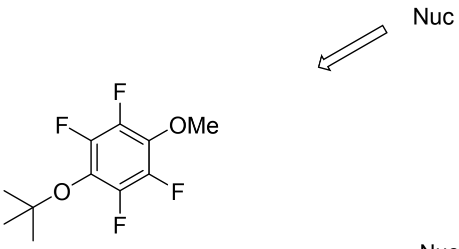

ortho: *para* : *meta* = 0.0: 3.5 : 1.0.

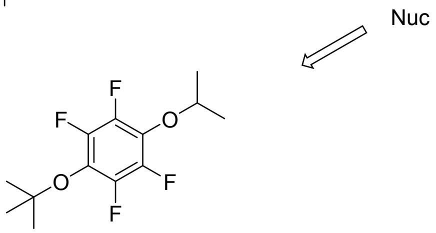

Pentafluoromethoxybenzene (9) with Na methoxide

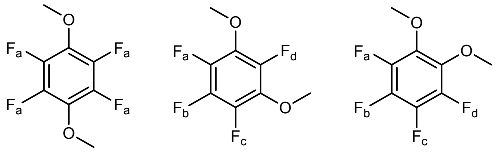

$^{19}F\{^1H\}$ NMR (470 MHz, Chloroform-*d*)

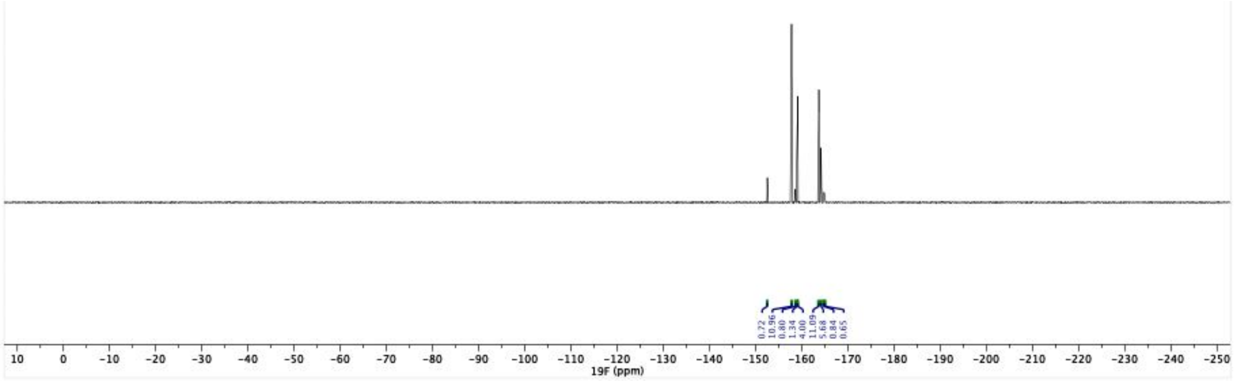

$^{19}F\{^1H\}$ NMR (470 MHz, Chloroform-*d*) enlarged

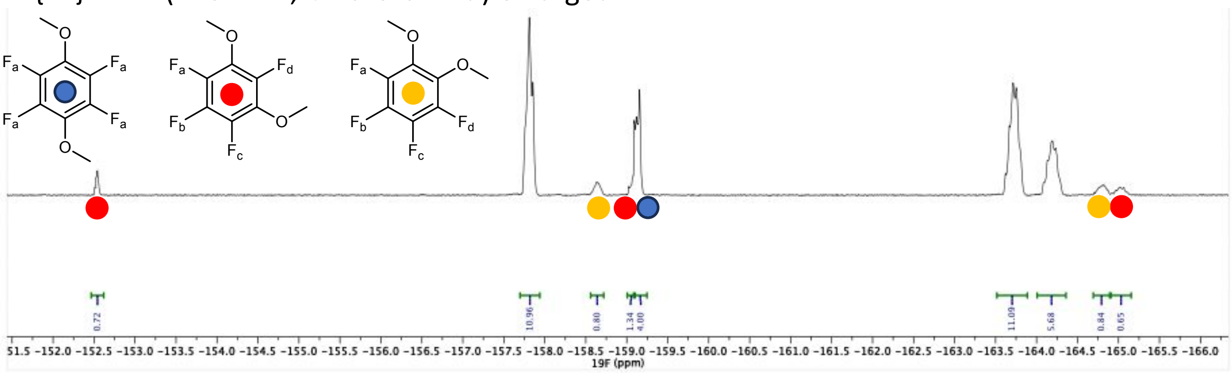

Pentafluoromethoxybenzene (9) with Na isopropoxide

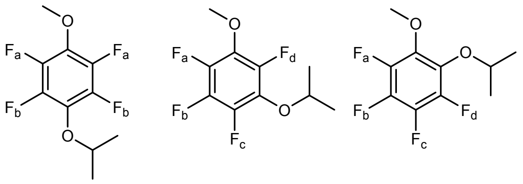

$^{19}F\{^1H\}$ NMR (470 MHz, Chloroform-*d*)

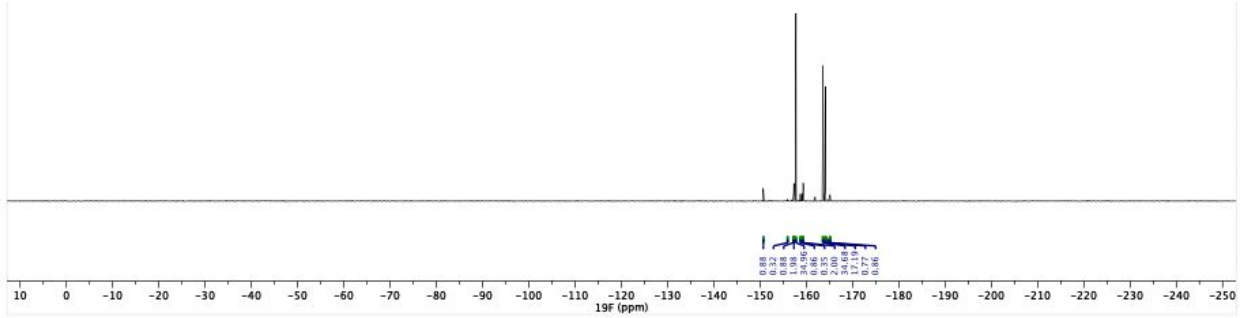

$^{19}F\{^1H\}$ NMR (470 MHz, Chloroform-*d*) enlarged

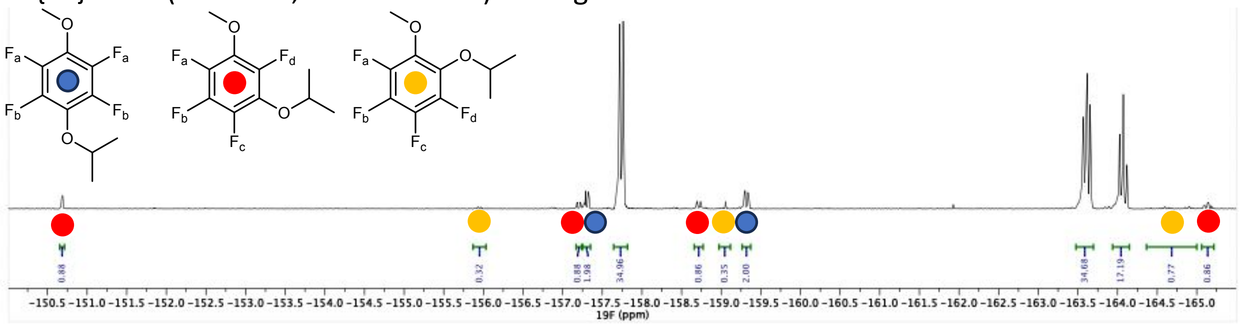

Pentafluoromethoxybenzene (9) with Na isobutoxide

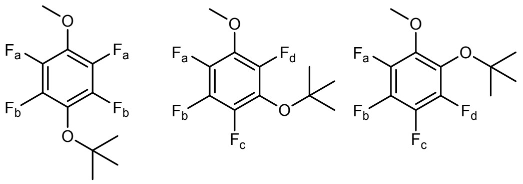

$^{19}F\{^1H\}$ NMR (470 MHz, Chloroform-*d*)

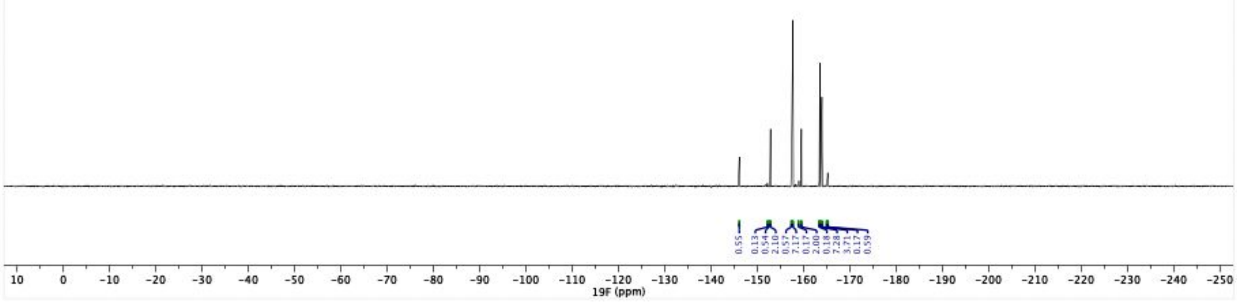

$^{19}F\{^1H\}$ NMR (470 MHz, Chloroform-*d*) enlarged

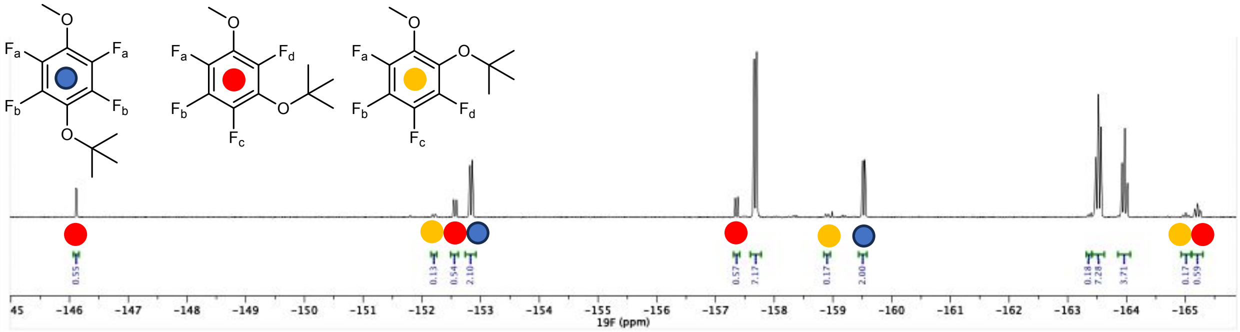

Pentafluoro-isopropoxyl-benzene (10) with Na methoxide

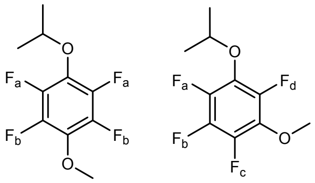

$^{19}F\{^1H\}$ NMR (470 MHz, Chloroform-*d*)

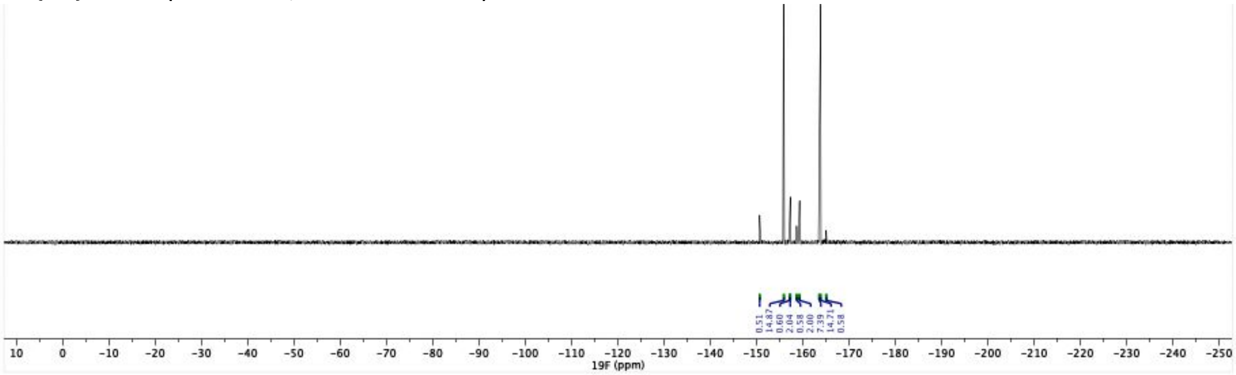

$^{19}F\{^1H\}$ NMR (470 MHz, Chloroform-*d*) enlarged

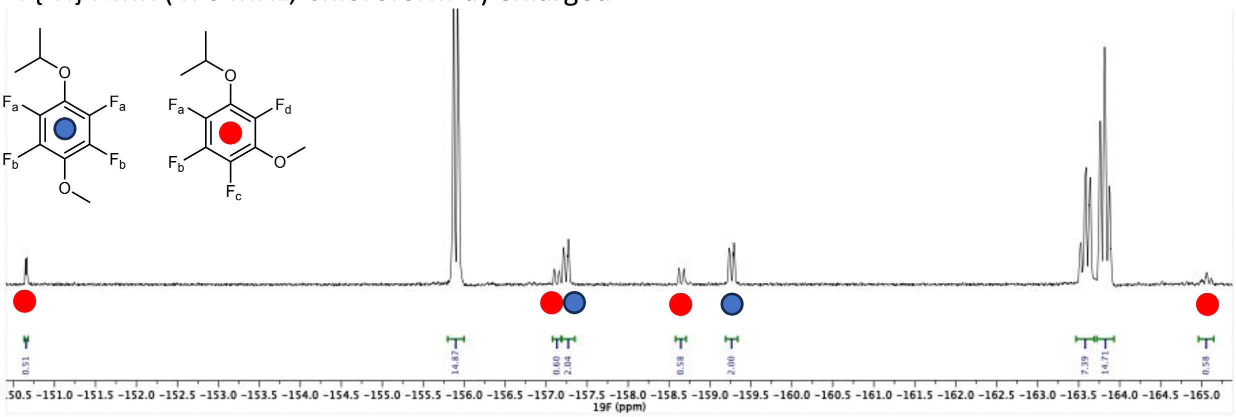

Pentafluoro-isopropoxyl-benzene (10) with Na isopropoxide

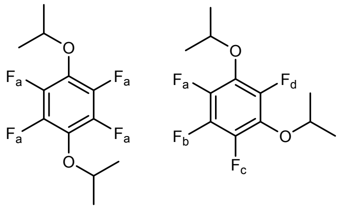

$^{19}F\{^{1}H\}$ NMR (470 MHz, Chloroform-*d*)

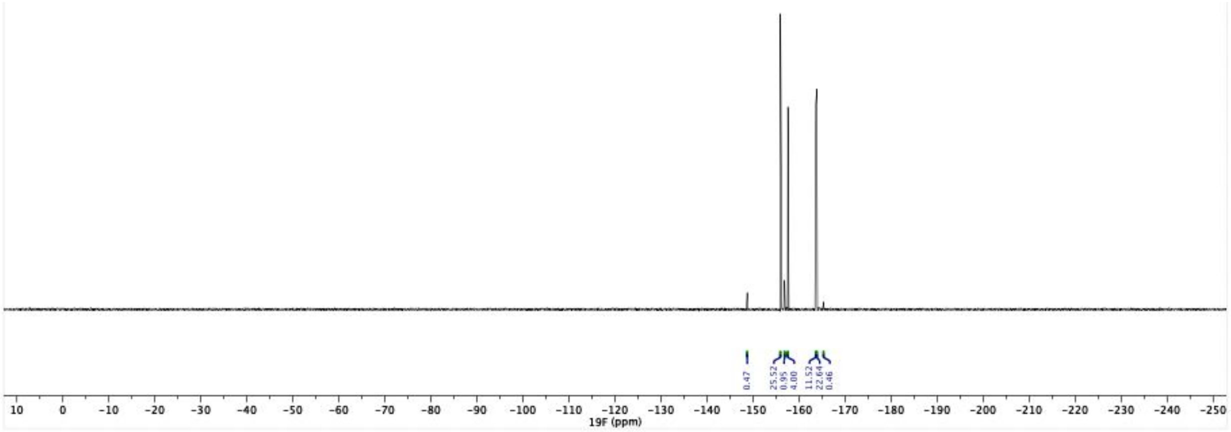

$^{19}F\{^{1}H\}$ NMR (470 MHz, Chloroform-*d*) enlarged

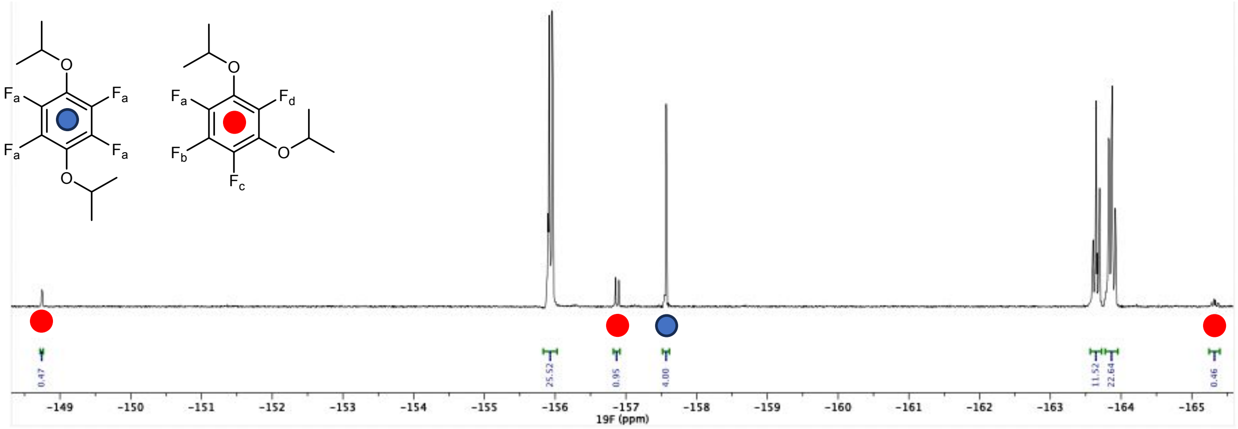

Pentafluoro-isopropoxyl-benzene (10) with Na *tert*-butoxide

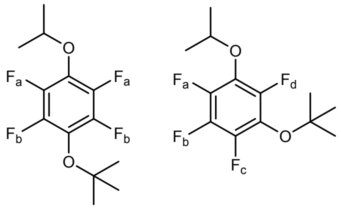

$^{19}F\{^1H\}$ NMR (470 MHz, Chloroform-*d*)

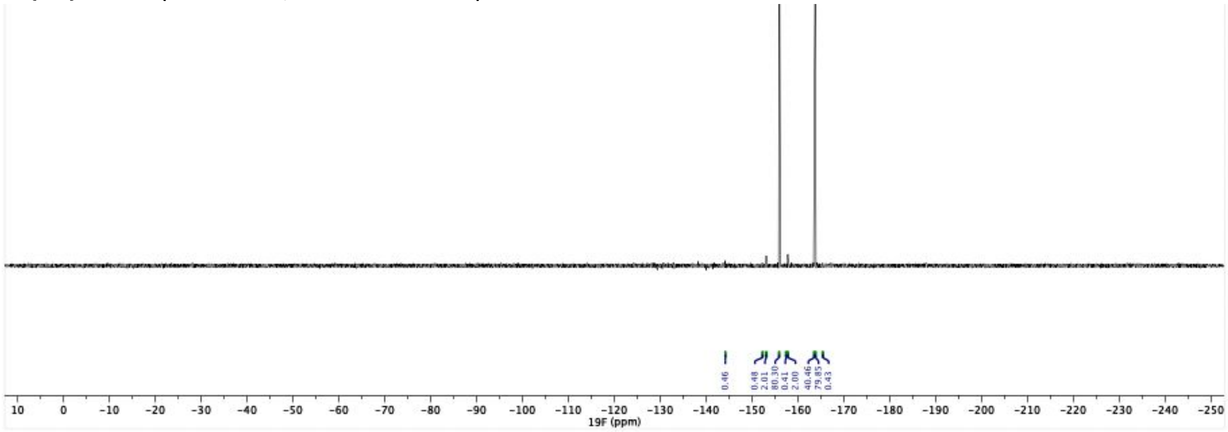

$^{19}F\{^1H\}$ NMR (470 MHz, Chloroform-*d*) enlarged

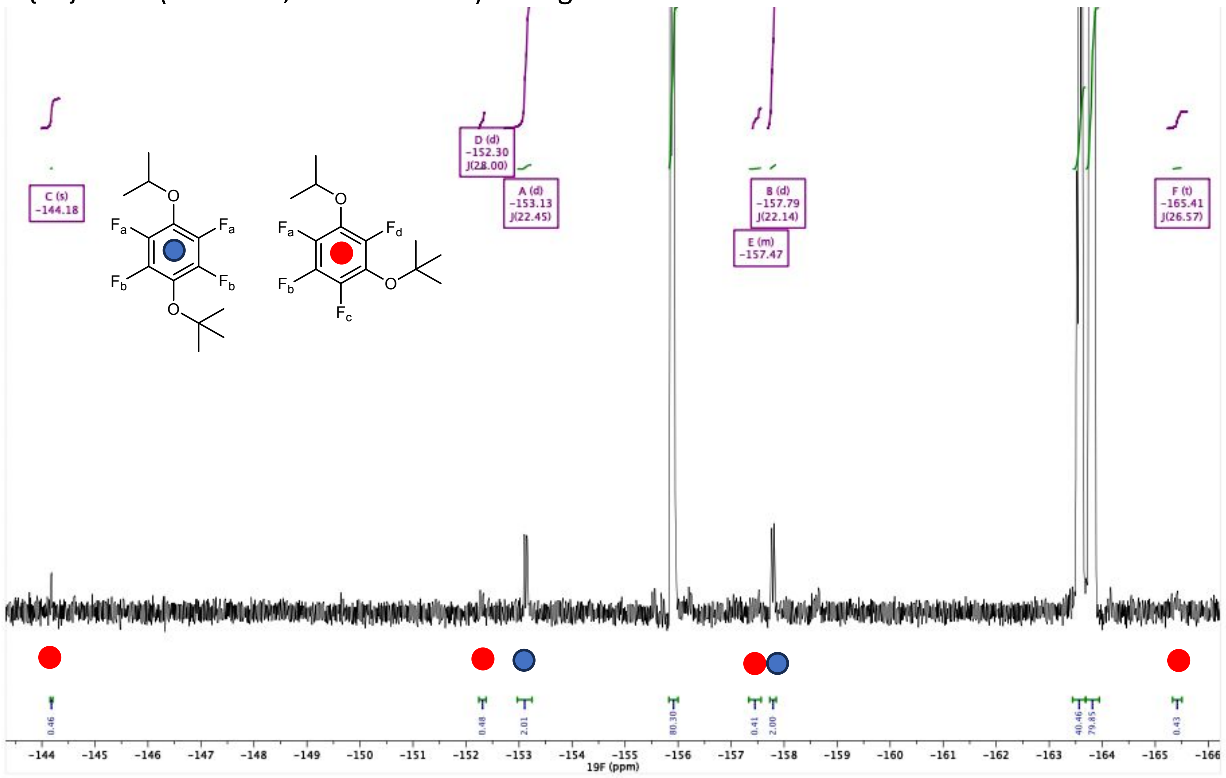

Pentafluoro-isobutoxyl-benzene (11) with Na methoxide

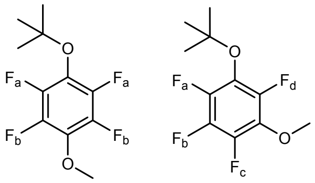

^{19}F{^{1}H} NMR (470 MHz, Chloroform-*d*)

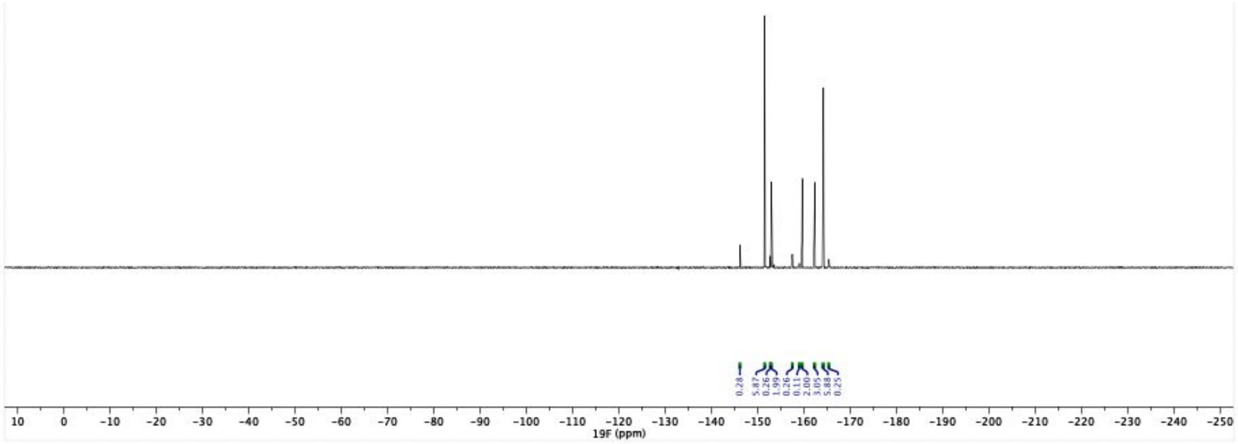

^{19}F{^{1}H} NMR (470 MHz, Chloroform-*d*) enlarged

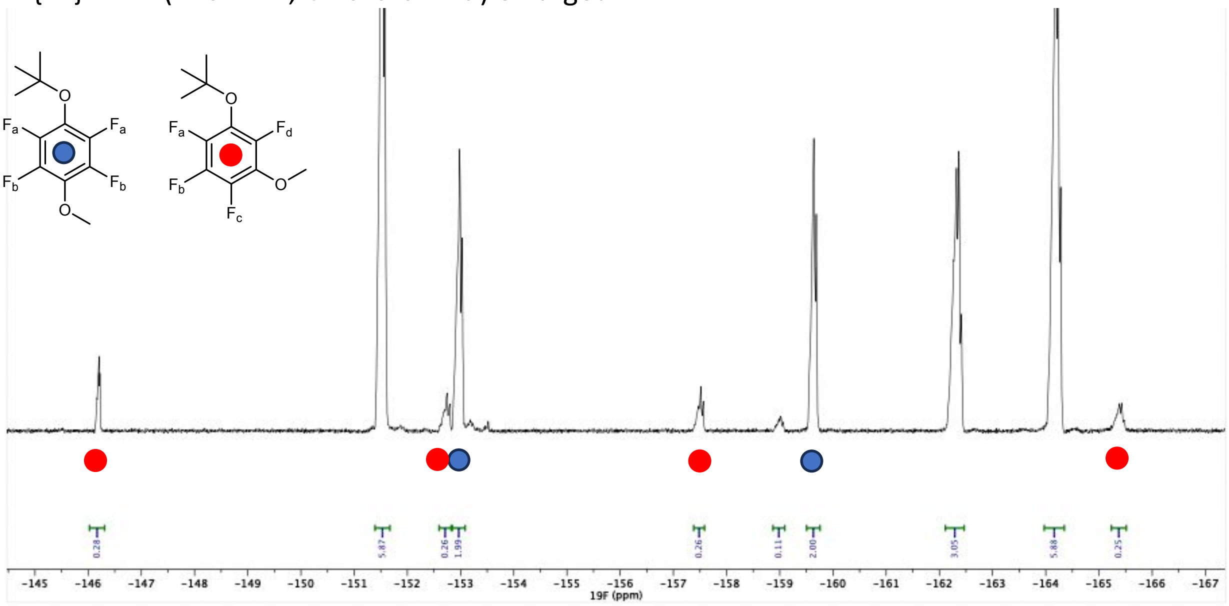

Pentafluoro-isobutoxyl-benzene (11) with Na isopropoxide

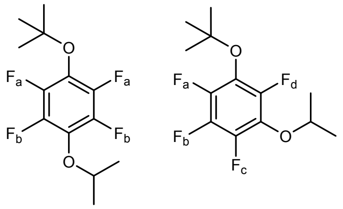

$^{19}F\{^1H\}$ NMR (470 MHz, Chloroform-*d*)

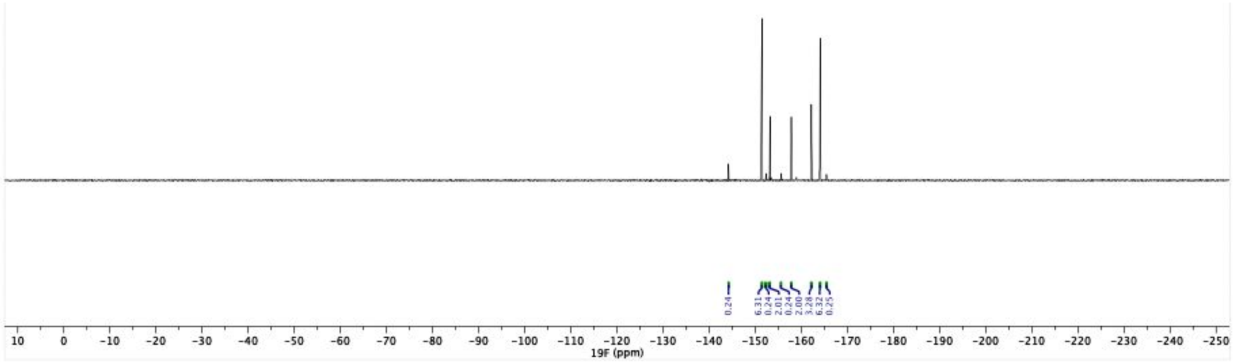

$^{19}F\{^1H\}$ NMR (470 MHz, Chloroform-*d*) zoomed in

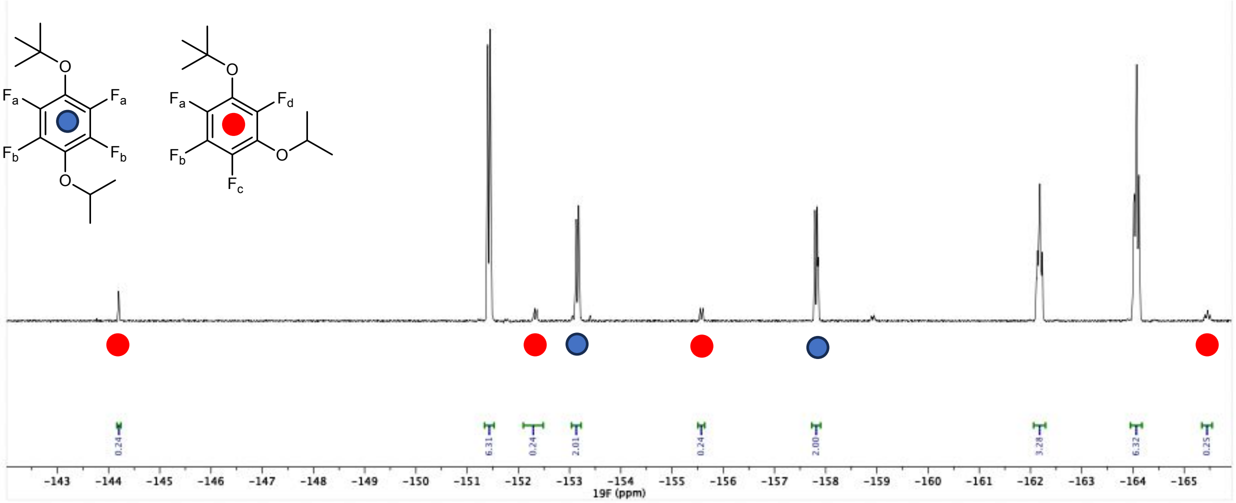

Pentafluoro-isobutoxyl-benzene (11) with Na isobutoxide

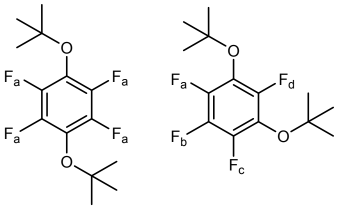

$^{19}F\{^1H\}$ NMR (470 MHz, Chloroform-*d*)

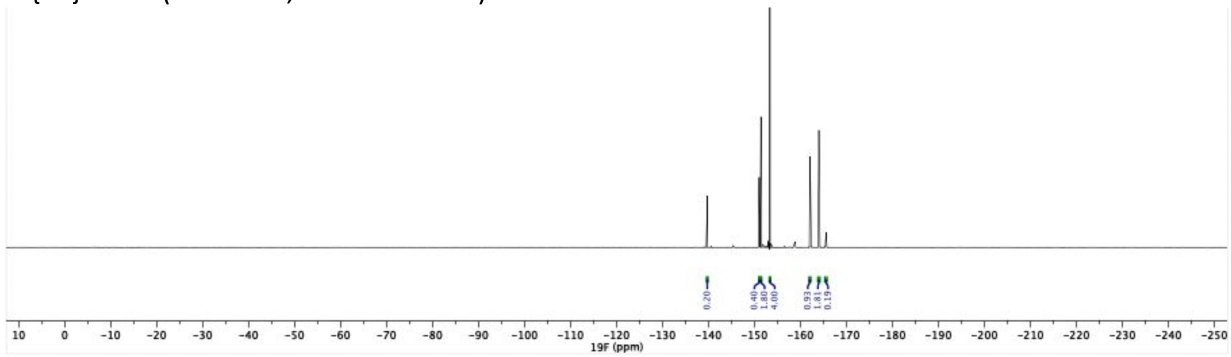

$^{19}F\{^1H\}$ NMR (470 MHz, Chloroform-*d*) enlarged

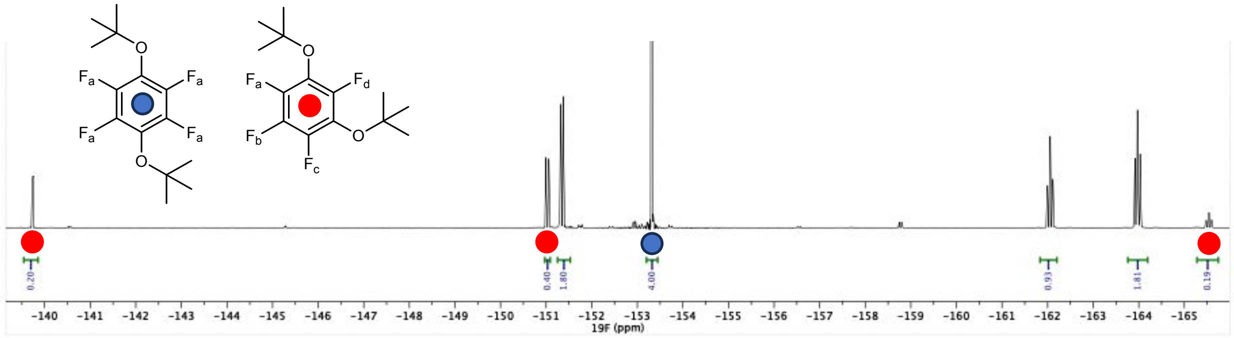

3. Synthesis protocols

General Procedure of aryl hydrogenations to generate 1a-d

Rhodium-CAAC-COD-Cl catalyst **3** (1.6—2 mol%) was added to an oven- dried 9 mL screw-cap vial or a 50 mL glass cylinder equipped with a stirring bar and activated 4 Å molecular sieves (0.2—3.2 g) and/or silica (0.2—1.6 g). Hexane (2—40 mL) and the aromatic substrate (1 eq) were added under argon. The glass vial/cylinder was placed in a stainless steel autoclave (150 mL) under an argon atmosphere and the autoclave was pressurized and depressurized with hydrogen gas three times before the indicated pressure was set (50—70 bar). The reaction mixture was stirred at 25—50 °C on an aluminium heating block, for 1—10 d. After the autoclave was carefully depressurized, the mixture was filtered through cotton inside a short glass column and washed with 10% methanol in DCM. The solvent was removed under reduced pressure to give the product, which was purified by chromatography.

4. Experimental Section

Tert-butoxypentafluorobenzene **11** was prepared as described by; C. L. Cheong, B. J. Wakefield*, J. Chem. Soc., Perkin Trans. 1.,* **1988**, 3301 – 3305.

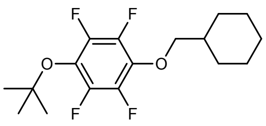

12a

A mixture of KOH (3.50 g, 63.4 mmol) in cyclohexanemethanol (4 mL, 31.7 mmol) was heated to 65°C on an aluminium heating block and left stirring for 20 min. Ether **11** (6 g, 24.98 mmol) was then added over 20 min with constant stirring and the reaction was heated to reflux for 16h. The reaction was then quenched by addition of water (50 mL). The product was extracted into DCM (4 x 25 mL) after washing with HCl (37%) and water. The organics were dried and the solvent was removed to give the title compound as a clear oil (77%, 6.39 g, 19.11 mmol) (including 10% *meta* product).

IR νmax (solid): 2927, 1494, 1041cm^{-1}; ^{1}H NMR (500 MHz, $CDCl_3$) δ : 3.95 (d, *J* = 6.4 Hz, 2H), 1.93 – 1.64 (m), 1.38 (s, 9H), 1.34 – 1.12 (m, 4H); ^{19}F{^{1}H} NMR (470 MHz, $CDCl_3$) δ : ^{19}F NMR (470 MHz, $CDCl_3$) δ : -153.0 (dd, *J* = 22.3, 5.7 Hz), -158.8 (dd, *J* = 21.7, 5.5 Hz); ^{13}C{^{1}H} NMR (126 MHz, $CDCl_3$) δ : 143.9 (apparent dd, *J* = 246.2, 12.8 Hz, Ar-C̲F), 140.7 (apparent d, *J* = 5.5 Hz), 84.4, 81.0, 38.5, 29.5, 28.4, 26.6, 25.8; HRMS (EI$^+$) m/z: [M]$^+$ Calcd for $C_{17}H_{22}F_4O_2$ 334.1550; Found 334.1546.

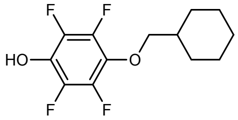

13a

TFA (3.7 mL) was added to a solution of **12a** (1.24 g, 3.71 mmol, 1 eq) in DCM (50 mL) and the reaction was stirred for 23 h. TFA and solvent were removed in vacuo and the product was purified by chromatography (0-10% EtOAc in hexane) to give **13a** (95%, 983 mg, 3.53 mmol) as a clear oil (including 10% *meta* product).

IR νmax (solid): 3310 (O-H), 2926, 1502, 1026 cm^{-1}; ^{1}H NMR (400 MHz, $CDCl_3$) δ : ^{1}H NMR (400 MHz, Chloroform-*d*) δ : 5.38 (s, 1H), 3.88 (d, *J* = 6.4 Hz, 2H), 1.94 – 1.64 (m, 7H), 1.37 – 1.12 (m, 4H); ^{19}F{^{1}H} NMR (377 MHz, $CDCl_3$) δ : -158.3 (dd, *J* = 21.8, 5.6 Hz), -164.7 (dd, *J* = 21.8, 5.7 Hz); ^{13}C{^{1}H} NMR (101 MHz, $CDCl_3$) δ : 142.1 (apparent d, *J* = 246.3 Hz), 138.0 (apparent d, *J* = 255.6 Hz), 130.2, 129.2, 81.4, 38.5, 29.6, 26.6, 25.8; HRMS (EI$^+$) m/z: [M]$^+$ Calcd for $C_{17}H_{22}F_4O_2$ 334.1550; Found 334.1554.

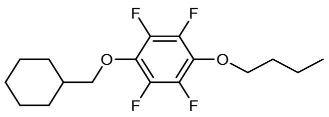

14a

Potassium carbonate (1.49 g, 10.78 mmol, 3 eq) was added to a solution of **13a** (1.0 g, 3.59 mmol, 1 eq) in acetonitrile (50 mL) and stirred at 85 °C for 1 h heated on an aluminium heating block. 1-Bromobutane (591 mg, 4.31 mmol, 1.2 eq) was then added over a 15 min period and the reaction was heated to 90 °C for 23 h. The mixture was then cooled to r.t., diluted with H_2O (100 mL), and the product was extracted into hexane (2x 50 mL). Purification by chromatography (1—20% EtOAc in hexane) gave **14a** (74%, 0.88g, 2.65 mmol) as a clear oil (including 10% *meta* product).

IR νmax (solid): 2927, 1498, 1300, cm^{-1}; ^{1}H NMR (500 MHz, $CDCl_3$) δ : 4.12 (t, *J* = 6.5 Hz, 2H), 3.92 (d, *J* = 6.4 Hz, 2H), 1.86 (apparent d, *J* = 13.3 Hz, 2H), 1.82 – 1.65 (m, 6H), 1.49 (h, *J* = 7.4 Hz, 2H), 1.35 – 1.13 (m, 3H), 1.05 (qd, *J* = 12.2, 3.4 Hz, 2H), 0.97 (t, *J* = 7.4 Hz, 3H); ^{19}F{H^1} NMR (470 MHz, $CDCl_3$) δ : -158.5; ^{13}C{^{1}H} NMR (126 MHz, $CDCl_3$) δ : 143.5 – 142.6 (m), 141.9 – 140.8 (m), 81.1, 75.5, 38.5, 32.0, 29.5, 26.6, 25.8, 19.0, 13.8; HRMS (EI$^+$) m/z: [M]$^+$ Calcd for $C_{17}H_{22}F_4O_2$ 334.1550; Found 334.1540.

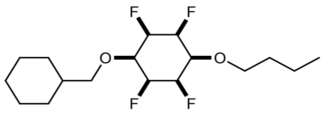

1a

Following the General Procedure, **14a** (620 mg, 1.85 mmol), **3** (20 mg, 2 mol%), 4Å mol sieves (8 g), silica (2 g), and hexane (40 mL) at r.t., for 4d and at 50 bar H_2 gave, after chromatography (60% DCM in hexane) **1a**, (53%, 335 mg, 0.98 mmol) as a white crystalline solid; m.p. 138-140 °C.

IR νmax (solid): 2924, 1377, 1085 cm^{-1}; ^1H NMR (500 MHz, $CDCl_3$) δ : 5.12 (d, *J* = 50.9 Hz, 2H), 4.51 – 4.22 (m, 3H), 3.57 (dd, *J* = 107.4, 39.4 Hz, 4H), 3.20 (q, *J* = 25.3, 23.7 Hz, 1H), 1.80 (d, *J* = 12.8 Hz, 2H), 1.66 (s, 9H), 1.41 (d, *J* = 8.1 Hz, 2H), 1.24 (q, *J* = 25.9, 20.2 Hz, 4H), 1.02 – 0.84 (m, 3H); ^{19}F NMR (470 MHz, $CDCl_3$) δ **Major conformer**: -206.4 (s, F_{eq}), -214.8 (s, F_{ax}); ^{19}F NMR (470 MHz, $CDCl_3$) δ **Minor conformer**: -206.5 (s, F_{ax}), -214.9 (s, F_{ax}); $^{13}C\{^1H\}$ NMR (126 MHz, $CDCl_3$) δ : 86.8 (dd, *J* = 191.5, 48.3 Hz), 76.4, 75.7, 73.9, 73.0, 38.3 (d, *J* = 61.0 Hz), 29.8, 26.7, 26.1, 19.3, 14.0; HRMS (EI$^+$) m/z: $[M]^+$ Calcd for $C_{17}H_{29}F_4O_2$ 341.2098; Found 341.2085.

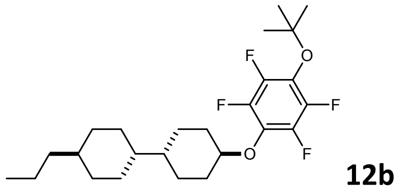

12b

A mixture of KOH (584 mg, 10.4 mmol) and 4'-propyl-[1,1'-bi(cyclohexan)]-4-ol (1.22 g, 5.41 mmol) in THF (10 mL) was heated to 65°C on an aluminium heating block and left stirring for 20 min. Ether **11** (0.7 mL, 1 g, 4.16 mmol) was added over 20 min with stirring and the reaction was heated to reflux for 24 h. The reaction was then quenched by addition of water (50 mL) and the product extracted into DCM and washed with HCl (37%). The organics were dried and the product was purified by chromatography (0-5% EtOAc in hexane) to give **12b** as a white solid (64%, 735 mg, 1.65 mmol) (including 13% *meta* product), m.p. = 60-61 °C.

IR νmax (solid): 2922, 1492, 1369, 1039, 979 cm^{-1}; ^1H NMR (500 MHz, $CDCl_3$) δ : 4.01 (tt, *J* = 10.6, 4.5 Hz, 1H), 2.12 (dd, *J* = 13.2, 3.8 Hz, 2H), 1.88 – 1.63 (m, 8H), 1.55 – 1.43 (m, 2H), 1.38 (s, 9H), 1.33 – 1.24 (m, 2H), 1.13 (t, *J* = 6.0 Hz, 4H), 1.09 – 0.91 (m, 5H), 0.87 (t, *J* = 8.2 Hz, 3H); ^{19}F NMR (470 MHz, $CDCl_3$) δ : -153.1 (dd, *J* = 22.7, 5.9 Hz), -157.5 (dd, *J* = 22.7, 5.8 Hz); $^{13}C\{^1H\}$ NMR (126

MHz, $CDCl_3$) δ : 145.3 – 143.3 (m), 143.3 – 141.0 (m), 84.5 (C2), 84.4 (C28), 42.6 (d, *J* = 61.5 Hz), 39.9, 37.7, 33.6, 32.7, 30.3, 28.4, 28.0, 20.2, 14.6; HRMS (ESI^+) m/z: $[M+Na]^+$ Calcd for $C_{25}H_{36}F_4O_2Na$ 467.2544; Found 467.2537.

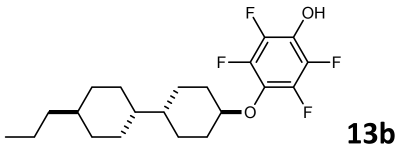

13b

TFA (1.57 mL) was added to a solution of **12b** (700 mg, 1.57 mmol, 1 eq) in DCM (50 mL). After addition the reaction was stirred for 23 h. TFA and solvent were then removed in vacuo and **13b** (94%, 571 mg, 1.47 mmol) was isolated after chromatography (0-20% EtOAc in hexane) as a white solid (including 11% *meta* product), m.p. = 120 - 121 °C.

IR vmax (solid): 2908 , 2360, 1494, 1037, 974 cm^{-1}; ^1H NMR (400 MHz, $CDCl_3$) δ : 5.08 (s, 1H), 3.92 (td, *J* = 11.0, 5.4 Hz, 1H), 2.16 – 2.06 (m, 2H), 1.86 – 1.65 (m, 8H), 1.47 (q, *J* = 12.4, 11.9 Hz, 2H), 1.36 – 1.24 (m, 2H), 1.13 (t, *J* = 5.9 Hz, 4H), 1.08 – 0.92 (m, 5H), 0.86 (t, *J* = 7.3 Hz, 3H); ^{19}F NMR (470 MHz, $CDCl_3$) δ : -156.8 (dd, *J* = 22.1, 5.9 Hz), -164.9 (dd, *J* = 22.2, 5.7 Hz); $^{13}C\{^1H\}$ NMR (101 MHz, $CDCl_3$) δ : 146.0 – 142.8 (m), 137.5 – 135.5 (m), 84.6, 42.6 (d, *J* = 48.3 Hz), 39.9, 37.7, 33.6, 32.6, 30.3, 28.0, 20.2, 14.6 ; HRMS (EI^-) m/z: $[M]^+$ Calcd for $C_{21}H_{27}F_4O_2$ 387.1953. Found 387.1960.

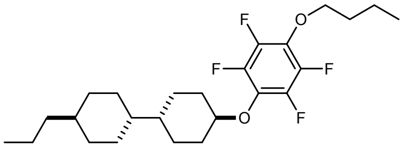

14b

Potassium carbonate (589 mg, 4.26 mmol, 3 eq) was added to a solution of **13b** (550 mg, 1.42 mmol, 1 eq) in acetonitrile (50 mL) and THF (10 mL) and the reaction was stirred at 85 °C for 1 h. Bromobutane (0.23 mL, 2.13 mmol, 1.2 eq) was then added over a 15 min period and the reaction was heated to 90 °C on an aluminium heating block for 23 hours. The mixture was then cooled to r.t., and diluted with H_2O (100 mL), and the products extracted into hexane and purified by

chromatography (1-20% EtOAc in hexane) to yield the title compound (66%, 414 mg, 0.93 mmol) as a gummy solid (including 10% *meta* product), m.p. = 38-40 °C.

IR vmax (solid): 2922, 1494, 1039, 981, cm^{-1}; ^{1}H NMR (500 MHz, $CDCl_3$) δ : 4.13 (t, *J* = 6.5 Hz, 2H), 3.97 (td, *J* = 10.8, 5.3 Hz, 1H), 2.14 – 2.07 (m, 2H), 1.74 (tq, *J* = 20.7, 10.7 Hz, 8H), 1.49 (dt, *J* = 14.6, 7.3 Hz, 4H), 1.30 (dq, *J* = 15.4, 8.0 Hz, 2H), 1.13 (t, *J* = 5.9 Hz, 4H), 1.10 – 0.99 (m, 7H) 0.97 (t, *J* = 7.4 Hz, 3H), 0.87 (t, *J* = 7.3 Hz, 3H); $^{19}F\{^{1}H\}$ NMR (470 MHz, $CDCl_3$) δ : -157.1 (dd, *J* = 21.9, 5.7 Hz), -158.6(dd, *J* = 21.9, 5.8 Hz); $^{13}C\{^{1}H\}$ NMR (126 MHz, $CDCl_3$) δ : 144.2 – 141.1 (m), 137.7 – 135.6 (m), 84.6, 75.4, 42.6 (d, *J* = 61.3 Hz), 39.9, 37.7, 33.6, 32.7, 32.0, 30.3, 28.0, 20.2, 19.0, 14.6, 13.9; HRMS (ESI$^+$) m/z: $[M+Na]^+$ Calcd for $C_{25}H_{36}F_4O_2Na$ 467.2549; Found 467.2544.

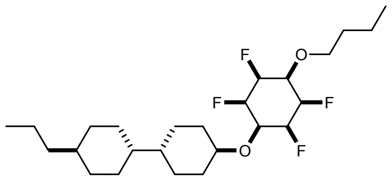

1b

Following the General Procedure, **14b** (390 mg, 0.88 mmol), **3** (10 mg, 2 mol%), 4Å mol sieves (1 g), silica (500 mg), and hexane (20 mL) at r.t., for 3 d, and at 50 bar H_2 gave, after chromatography (30-60% DCM in hexane), **1b** (72%, 283 mg, 0.63 mmol) as a white solid (including 6% *meta* product). m.p. = 236-238 °C dec.

IR vmax (solid): 2922, 2362, 1450, 1043, 871 cm^{-1}; ^{1}H NMR (400 MHz, Chloroform-*d*) δ : 5.22 – 4.90 (m, 4H), 4.55 – 4.15 (m, 2H), 3.80 – 3.57 (m, 2H), 3.54 – 3.32 (m, 1H), 2.12 – 1.96 (m, 2H), 1.80 – 1.56 (m, 8H), 1.41 (td, *J* = 14.4, 6.8 Hz, 4H), 1.29 (dd, *J* = 16.2, 9.8 Hz, 2H,), 1.17 – 1.09 (m, 4H), 1.03 – 0.87 (m), 0.86 (t, *J* = 7.4 Hz, 3H); ^{19}F NMR (376 MHz, $CDCl_3$) δ **Major conformer**: -206.3 ($2F_{eq}$), -214.7 (s, $2F_{ax}$); ^{19}F NMR (376 MHz, $CDCl_3$) δ **Minor conformer**: -206.8 ($2F_{ax}$), -214.5 (s, $2F_{eq}$); ^{19}F NMR (376 MHz, $CDCl_3$) δ ***meta* product** (6%): -206.6 (t, *J* = 11.5 Hz), -214.1 (t, *J* = 24.5 Hz), -215.3 (apparent dd, *J* = 24.1, 11.5 Hz); $^{13}C\{^{1}H\}$ NMR (101 MHz, $CDCl_3$) δ : 148.7 – 145.3 (m), 140.1 – 137.0 (m), 85.5, 75.1, 42.7 (d, *J* = 51.4 Hz), 39.9, 37.7, 33.7, 32.7, 32.1, 30.3, 28.2, 20.2, 19.1, 14.6, 14.0; HRMS (ESI$^+$) m/z: $[M+Na]^+$ Calcd for $C_{25}H_{42}F_4O_2Na$ 473.3019; Found 473.3013.

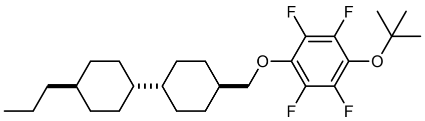

12c

KOH (584 mg, 10.4 mmol), alcohol **13a** (1.29 g, 5.41 mmol), THF (10 mL), and 1-(*tert*-butoxy)-2,3,4,5,6-pentafluorobenzene (~0.7 mL, 1.0 g, 4.16 mmol) were stirred for 24 h at 75°C on an aluminium heating block. After purification by chromatography (0-5% EtOAc in hexane) **12c** (52%, 987.8 mg, 2.15 mmol) was isolated as clear oil (including 14% *meta* product).

IR νmax (solid): 2906, 2017, 1506, 1035, cm^{-1}; ^{1}H NMR (500 MHz, $CDCl_3$) δ : 3.94 (d, *J* = 6.4 Hz, 2H), 1.96 – 1.89 (m, 2H), 1.80 – 1.66 (m, 7H), 1.38 (t, *J* = 1.2 Hz, 9H), 1.29 (p, *J* = 7.2 Hz, 2H), 1.14 (t, *J* = 6.0 Hz, 3H), 1.08 – 0.91 (s, 10H), 0.87 (t, *J* = 7.3 Hz, 3H); ^{19}F NMR (470 MHz, $CDCl_3$) δ ***para* product**: -153.0 (dd, *J* = 22.3, 6.0 Hz), -158.8 (dd, *J* = 22.3, 6.0 Hz); ^{19}F NMR (470 MHz, $CDCl_3$) δ ***meta* product**: -145.4 (d, *J* = 5.8 Hz), -152.9 (dd, *J* = 22.9, 1.2 Hz), -156.6 (d, *J* = 21.5 Hz), -165.3 (td, *J* = 22.2, 5.8 Hz); ^{13}C{^{1}H} NMR (126 MHz, $CDCl_3$) δ : 143.2 – 142.3 (m), 136.8 – 136.2 (m), 81.1 ($O\underline{C}H_2$), 67.9, 43.5, 43.4, 40.0, 38.8, 37.8, 33.7, 30.4 – 29.1 (m), 28.4, 20.2, 14.5; HRMS (ESI^{+}) m/z: $[M+Na]^{+}$ Calcd for $C_{26}H_{38}F_4O_2Na$ 481.2700; Found 481.2705.

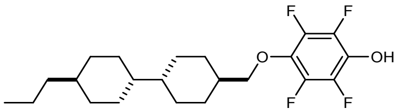

13c

TFA (1.45 mL) was added to a solution of **12c** (664 mg, 1.45 mmol, 1 eq) in DCM (50 mL) and the reaction was stirred for 23 h. TFA and solvent were then removed in vacuo and the product was purified by chromatography (0-20% EtOAc in hexane) to give **13c** (82%, 479.8 mg, 1.19 mmol) as a white solid (including 14% *meta* product), m.p. 119-120 °C.

IR vmax (solid): 2908, 1483, 1375, 1020, 1015, cm^{-1}; ^{1}H NMR (500 MHz, $CDCl_3$) δ : 5.10 (s, 1H, O<u>H</u>), 3.88 (d, *J* = 6.4 Hz, 2H), 1.97 - 1.87 (m, 2H), 1.82 - 1.66 (m, 7H), 1.29 (apparent td, *J* = 13.7, 12.6, 6.3 Hz, 2H), 1.17 – 1.10 (m, 3H), 1.08 – 0.91 (m, 10H), 0.87 (t, *J* = 7.3 Hz, 3H); $^{19}F\{^1H\}$ NMR (470 MHz, $CDCl_3$) δ : -158.2 (dd, *J* = 21.8, 5.6 Hz), -164.8 (dd, *J* = 21.9, 5.7 Hz); $^{13}C\{^1H\}$ NMR (126 MHz, $CDCl_3$) δ : 143.1 – 142.4 (m), 136.6 – 135.5 (m), 81.4 (O<u>C</u>H_2), 43.5, 43.4, 40.0, 38.8, 37.8, 33.7, 30.3 - 29.3 (m), 20.2, 14.6; HRMS (ESI^+) m/z: $[M+Na]^+$ Calcd for $C_{22}H_{30}F_4O_2Na$ 425.2074; Found 425.2087.

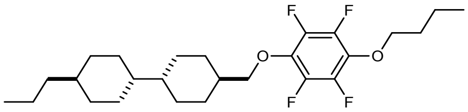

14c

Potassium carbonate (328 mg, 2.37 mmol, 3 eq) was added to a solution of **13c** (318 mg, 0.79 mmol, 1 eq) in acetonitrile (20 mL) and THF (10 mL) and the reactions was stirred at 85 °C on an aluminium heating block for 1 h. Bromobutane (0.11 mL, 0.95 mmol, 1.2 eq) was then added over a 15 min period and the reaction was heated to 85 °C for 23 h. The mixture was cooled to r.t., diluted with H_2O (50 mL), and extracted into hexane. After purification by chromatography (1-20% EtOAc in hexane) **14c** was isolated (67%, 242 mg, 0.53 mmol) as an off white solid (including 13% *meta* product). m.p. 76-78 °C.

IR vmax (solid): 285, 1498, 1107, 1043, cm^{-1}; ^{1}H NMR (500 MHz, $CDCl_3$) δ : 4.12 (t, *J* = 6.5 Hz, 2H), 3.91 (d, *J* = 6.4 Hz, 2H), 1.94 – 1.88 (m, 2H), 1.81 – 1.66 (m, 10H), 1.55 – 1.43 (m, 2H), 1.4(m, 3H), 1.30 (dq, *J* = 14.8, 7.5 Hz, 3H), 1.08 – 0.99 (m, 8H), 0.97 (t, *J* = 7.4 Hz, 3H), 0.87 (t, *J* = 7.3 Hz, 3H); $^{19}F\{H^1\}$ NMR (470 MHz, $CDCl_3$) δ : -158.5; $^{13}C\{^1H\}$ NMR (126 MHz, $CDCl_3$) δ : 143.3 – 142.3 (m, Ar-<u>C</u>F), 136.9 – 135.4 (m), 81.2, 75.5, 43.5, 43.4, 40.0, 38.8, 37.8, 33.7, 32.0, 30.3 - 29.3 (m), 20.2, 19.0, 14.6, 13.9; HRMS (EI^+) m/z: $[M]^+$ Calcd for $C_{26}H_{38}F_4O_2$ [M] 458.2802; Found 458.2822.

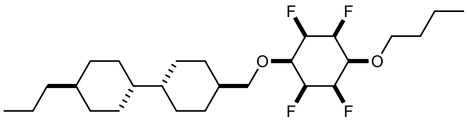

1c

Following the General Procedure **14c** (550 mg, 1.2 mmol), **3** (12.3 mg, 2 mol%), 4Å mol sieves (8 g), silica (4 g), and hexane (40 mL) at r.t., for 5d and at 50 bar H_2 gave, after chromatography (30-60% DCM in hexane), **1c** (54%, 302.3 mg, 0.65 mmol) as a rubbery white solid (including 12% *meta* product), m.p. 174-175 °C.

IR νmax (solid): 2910, 1107, 1043, cm^{-1}; ^{1}H NMR (500 MHz, $CDCl_3$) δ : 5.32 – 5.03 (m), 4.53 – 4.22 (m, 2H), 3.68 (t, 4H), 3.49 (dd, *J* = 31.1, 6.0 Hz, 2H), 3.37 – 3.10 (m, 2H), 1.88 (d, *J* = 10.5 Hz, 3H), 1.80 – 1.57 (m, 10H), 1.44 (p, *J* = 7.4 Hz, 3H), 1.36 – 1.26 (m, 9H), 1.20 – 1.11 (m, 3H), 0.98 – 0.86 (m, 6H); ^{19}F NMR (470 MHz, $CDCl_3$) δ **Major conformer**: -206.4 ($F_{eq,}$), -214.7 (s, $F_{ax,}$); ^{19}F NMR (470 MHz, $CDCl_3$) δ **Minor conformer**: -206.5 (F_{ax}), -214.9 (s, F_{eq}); ^{19}F NMR (470 MHz, $CDCl_3$) δ ***meta* product** (12%): -206.8 (t, *J* = 11.5 Hz), -214.2 (t, *J* = 24.5 Hz), -215.3 – -215.4 (m); ^{13}C{^{1}H} NMR (101 MHz, $CDCl_3$) δ : 112.6 – 105.7 (m), 80.0, 68.1, 43.6, 40.0, 37.8, 33.8, 31.8, 30.6 – 29.1 (m), 20.2, 19.2, 14.6, 14.0; HRMS (ESI^{+}) m/z: [M+Na]$^{+}$ Calcd for $C_{26}H_{44}F_4O_2Na$ 487.3175; Found 487.3170.

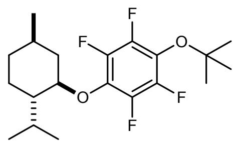

12d

A mixture of KOH (584 mg, 10.41 mmol), L-menthol (1.63 g, 10.41 mmol, 2.5 eq), THF (10 mL), and **11** (1 g, 4.164 mmol) was stirred for 24 h, 75°C on an aluminium heating block. The reaction was quenched by addition of water and the product extracted into DCM and washed with HCl (37%). The organics were dried and the product was purified by chromatography (0-5% EtOAc in hexane) to give **12d** (38%, 588.8 mg, 1.564 mmol) as clear oil (including 8% *meta* product).

IR vmax (solid): 2895, 1467, 1342, 1093 cm^{-1}; ^{1}H NMR (400 MHz, CDCl3) δ : 4.04 (td, *J* = 10.6, 4.4 Hz), 2.40 (pd, *J* = 7.0, 2.6 Hz), 1.88 (d, *J* = 11.9 Hz), 1.77 – 1.63 (m, 2H), 1.54 (ddt, *J* = 13.0, 10.2, 3.2 Hz), 1.44 – 1.24 (m), 1.19 – 0.99 (m), 0.97 (d, *J* = 7.0 Hz, 3H), 0.90 (d, *J* = 6.5 Hz, 3H), 0.87 (d, *J* = 6.9 Hz, 3H); ^{19}F NMR (377 MHz, $CDCl_3$) δ **Major *Para* Product**: -153.1 (dd, *J* = 22.6, 6.2 Hz, 2F), -157.2 (dd, *J* = 22.2, 6.3 Hz, 2F) ; ^{19}F NMR (377 MHz, $CDCl_3$) δ ***Meta* product** (8%): -143.7 (d, *J* = 6.0

Hz), -152.5 (d, J = 22.8 Hz), -154.8 (d, J = 22.0 Hz), -165.4 (td, J = 22.5, 6.1 Hz); $^{13}C\{^1H\}$ NMR (101 MHz, $CDCl_3$) δ : 143.9 (ddt, J = 245.8, 12.6, 4.5 Hz, 142.4 (ddt, J = 245.9, 13.9, 5.4 Hz), 84.4, 84.2, 48.6, 40.6, 34.3, 31.6, 28.4 (m), 25.8, 23.3, 22.2 , 21.1, 16.1; HRMS (ESI^+) m/z: $[M+Na]^+$ Calcd for $C_{20}H_{28}F_4O_2Na$ 399.1918; Found 399.1918.

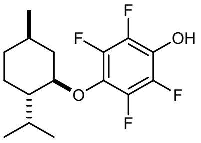

13d

TFA (2.6 mL) was added to a solution of **12d** (952 mg, 2.53 mmol, 1 eq) in DCM (50 mL) and the reaction was stirred for 48 h. The TFA and solvent were then removed in vacuo and the product was purified by chromatography (0-20% EtOAc in hexane) to give 1**3d** (quantitative, 851 mg, 2.5 mmol) as a clear oil (including 8% *meta* product).

IR νmax (solid): 3660, 2900, 1320, 1097, cm^{-1}; ^1H NMR (400 MHz, $CDCl_3$) δ : 5.60 – 5.02 (m, 1H, OH), 3.94 (td, J = 10.6, 4.4 Hz, 1H), 2.42 (pd, J = 7.0, 2.6 Hz, 1H), 1.86 (d, J = 12.4 Hz, 1H), 1.70 (ddq, J = 16.2, 12.7, 3.3 Hz, 2H), 1.52 (ddd, J = 16.3, 9.5, 3.1 Hz, 1H), 1.26 (t, J = 7.2 Hz, 2H), 1.16 – 1.01 (m, 2H), 0.97 (d, J = 7.1 Hz, 3H), 0.90 (d, J = 6.5 Hz, 3H), 0.87 (d, J = 6.9 Hz, 3H); $^{19}F\{^1H\}$ NMR (376 MHz, $CDCl_3$) δ : -156.7 (dq, J = 19.7, 5.9 Hz, 2F, F_b), -164.7 – -164.9 (m, 2F); $^{13}C\{^1H\}$ NMR (101 MHz, $CDCl_3$) δ : 140.5, 134.7, 113.9, 101.5, 84.3, 48.6, 40.4, 34.3, 31.6, 25.7, 23.2, 22.3, 21.1, 16.1; HRMS (ESI^-) m/z: $[M-H]^-$ Calcd for $C_{16}H_{19}F_4O_2$ 319.1321; Found 319.1327.

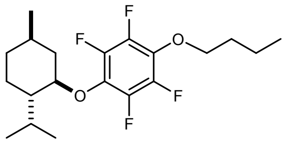

14d

Potassium carbonate (2.57 g, 18.63 mmol, 3 eq) was added to a solution of **13d** (1.99 g, 6.21 mmol, 1 eq) in acetonitrile (20 mL) and THF (10 mL) and stirred at 85 °C for 30 min on an aluminium heating block. Bromobutane (0.88 mL, 8.07 mmol, 1.3 eq) was then added over a 15

min period and the reaction was heated to 75 °C for 24 h on an aluminium heating block. The mixture was cooled to r.t., diluted with H_2O (50 mL), and the product extracted into hexane. Purification gave the title compound (65%, 1.51 g, 4.01 mmol) as a clear liquid (including 8% *meta* product).

IR νmax (solid): 2873, 1496, 1340, 1095 cm^{-1}; ^{1}H NMR (400 MHz, $CDCl_3$) δ : 4.17 – 4.11 (m, 2H), 4.00 (td, *J* = 10.6, 4.4 Hz, 1H), 2.40 (pd, *J* = 6.9, 2.6 Hz, 1H), 1.87 (d, *J* = 12.2 Hz, 1H), 1.80 – 1.63 (m, 4H), 1.60 – 1.46 (m, 3H), 1.39 – 1.31 (m, 2H), 1.18 – 1.02 (m, 2H), 1.00 – 0.94 (m, 6H), 0.92 – 0.85 (m, 6H); ^{19}F{H^1} NMR (377 MHz, $CDCl_3$) δ : -156.8 (dd, *J* = 21.8, 5.9 Hz, 2F), -158.7 (dd, *J* = 21.9, 5.7 Hz, 2F); ^{13}C{^{1}H} NMR (101 MHz, $CDCl_3$) δ : 144.0 – 143.1 (m), 141.6 – 140.5 (m), 131.7 – 130.3 (m), 84.2, 75.4, 48.6, 40.5, 34.3, 32.0, 31.6, 25.7, 23.3, 22.2, 21.1, 19.0, 16.1, 13.9; HRMS (ESI$^+$) m/z: [M+Na]$^+$ Calcd for $C_{20}H_{28}F_4O_2Na$ 399.1923; Found 399.1918.

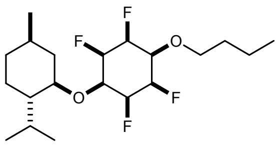

1d

Following the General Procedure, **14d**, (400 mg, 1.06 mmol), **3** (11 mg, 1.8 mol%), 4Å mol sieves (8 g), silica (4 g), and hexane (40 mL) at r.t., for 4d and at 50 bar H_2 gave, after chromatography (50-70% DCM in hexane) **1d** (64%, 261 mg, 0.682 mmol) as a white powder (including 3% *meta* product). m.p. 99-100 °C.

IR νmax (solid): 2908 , 1465, 1105, 1095, cm^{-1}; ^{1}H NMR (400 MHz, $CDCl_3$) δ : 5.26 – 4.75 (m, 2H), 4.63 – 4.16 (m, 2H), 3.80 – 3.54 (m, 2H), 3.43 – 3.16 (m, 2H), 2.62 – 2.23 (m, 1H), 2.18 – 1.89 (m, 2H), 1.74 – 1.47 (m, 5H), 1.47 – 1.16 (m, 6H), 0.90 (d, *J* = 6.5 Hz, 9H), 0.75 (d, *J* = 6.9 Hz, 3H); ^{19}F NMR (470 MHz, $CDCl_3$) δ **Major conformer**: -206.4 (dd, J = 47.8, 11.2 Hz), -214.5 (d, *J* = 13.3 Hz); ^{19}F NMR (470 MHz, $CDCl_3$) δ **Minor conformer**: -205.7 (d, *J* = 10.2 Hz), -214.1 (d, *J* = 16.9); ^{19}F NMR (470 MHz, $CDCl_3$) δ ***meta* product** (3%): -206.6 (t, *J* = 11.3 Hz), -214.2 (td, *J* = 24.4, 17.1 Hz), -214.9

– -215.5 (m); $^{13}C\{^1H\}$ NMR (101 MHz, $CDCl_3$) δ : 88.2 – 85.2 (m), 79.0, 76.2, 74.1, 48.2, 40.8, 34.4, 32.2, 31.7, 31.1, 23.1, 22.4, 19.1, 16.0, 14.0; HRMS (ESI^+) m/z: $[M+Na]^+$ Calcd for $C_{20}H_{34}F_4O_2Na$ 405.2393; Found 405.2387.

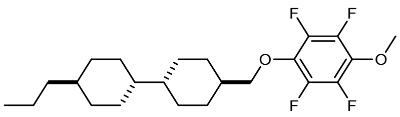

14e

Potassium carbonate (484 mg, 3.5 mmol, 3 eq) was added to a solution of 4-(cyclohexylmethoxy)-2,3,5,6-tetrafluorophenol (470 mg, 1.17 mmol, 1 eq) in acetonitrile (50 mL) and stirred at 85 °C for 1 h. Methyl iodide (0.1 mL, 1.99 mg, 1.4 mmol, 1.2 eq) was then added to the mixture over a 15 min period and the reaction was heated to 90 °C for 23 h on an aluminium heating block. The mixture was then cooled to r.t., diluted with H_2O (100 mL), and extracted into hexane (2x 50 mL). Purification by chromatography (1-20% EtOAc in hexane) gave **14e** (57%, 278.2 mg, 0.67 mmol) as a clear oil (including 15% *meta* product).

IR ν_{max} (solid): 2922, 1498, 1394, 1045, cm^{-1}; ^1H NMR (500 MHz, $CDCl_3$) δ : 3.99 (s, 3H), 3.91 (d, *J* = 6.4 Hz, 2H), 1.95 – 1.87 (m), 1.81 – 1.65 (m, 7H), 1.29 (dt, *J* = 14.4, 7.4 Hz, 2H), 1.14 (t, *J* = 6.0 Hz, 3H), 1.06 – 0.92 (m, 10H), 0.87 (t, *J* = 7.3 Hz, 3H); $^{19}F\{^1H\}$ NMR (470 MHz, $CDCl_3$) δ : -158.3 (dd, *J* = 21.4, 5.5 Hz), -159.1 (dd, *J* = 21.5, 5.7 Hz); $^{13}C\{^1H\}$ NMR (101 MHz, $CDCl_3$) δ : 141.9 (d, *J* = 244.4 Hz), 139.5 (m), 81.2, 43.5, 43.4, 40.0, 38.8, 37.8, 33.7, 30.5, 30.7 – 28.8 (m), 20.2, 14.6; HRMS (EI^+) m/z: $[M]^+$ Calcd for $C_{23}H_{32}F_4O_2$ 416.2333; Found 416.2331.

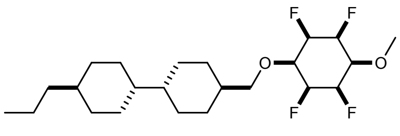

1e

Following the General Procedure, **14e**) (110 mg, 0.26 mmol), **3** (3.0 mg, 1-2 mol%), 4Å mol sieves (1 g), silica (250 mg), and hexane (20 mL) at r.t, for 5 d and at 50 bar H_2 gave, after

chromatography (50-70% DCM in hexane) **1e** (45%, 50 mg, 0.12 mmol) as a white powder (including 15% *meta* product). m.p. 210-211 °C.

IR ν_{max} (solid): 2906, 1444, 1392, 1107, 1047, cm^{-1}; ^1H NMR (400 MHz, $CDCl_3$) δ : 5.14 (d, *J* = 50.0 Hz, 2H), 4.56 – 4.20 (m, 2H), 3.79 – 3.68 (m, 2H), 3.58 (s, 3H), 3.44 (d, *J* = 6.5 Hz, 2H), 1.85 (apparent dt, *J* = 6.6, 3.0 Hz, 2H), 1.79 – 1.50 (m, 8H), 1.35 – 1.22 (m, 3H), 1.18 – 1.08 (m, 3H), 0.95 (apparent dd, *J* = 20.0, 9.7 Hz, 8H), 0.86 (t, *J* = 7.3 Hz, 3H); ^{19}F NMR (470 MHz, $CDCl_3$) δ **Major conformer**: -206.6 ($2F_{eq}$), -215.1 (s, $2F_{ax}$); ^{19}F NMR (470 MHz, $CDCl_3$) δ **Minor conformer**: -206.5 ($2F_{ax}$), -215.0 (s, $2F_{eq}$); ^{19}F NMR (470 MHz, $CDCl_3$) δ ***meta* product** (15%): -206.9 (t, *J* = 11.2 Hz), -214.4 (t, *J* = 24.5 Hz), -215.5 (ddd, *J* = 30.5, 24.5, 11.1 Hz); $^{13}C\{^1H\}$ NMR (101 MHz, $CDCl_3$) δ : 93.0 – 90.5 (m), 87.9 – 85.2 (m), 72.8, 66.2, 43.5, 40.0, 37.8, 33.8, 30.6 – 29.2 (m), 20.2, 14.6; HRMS (ESI^+) m/z: $[M+Na]^+$ Calcd for $C_{23}H_{38}F_4O_2Na$ 445.2706. Found 445.2700.

1-(*tert*-butoxy)-4-(cyclohexylmethoxy)-2,3,5,6-tetrafluorobenzene 12a

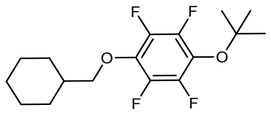

$^{19}F\{^1H\}$ NMR (470 MHz, Chloroform-*d*)

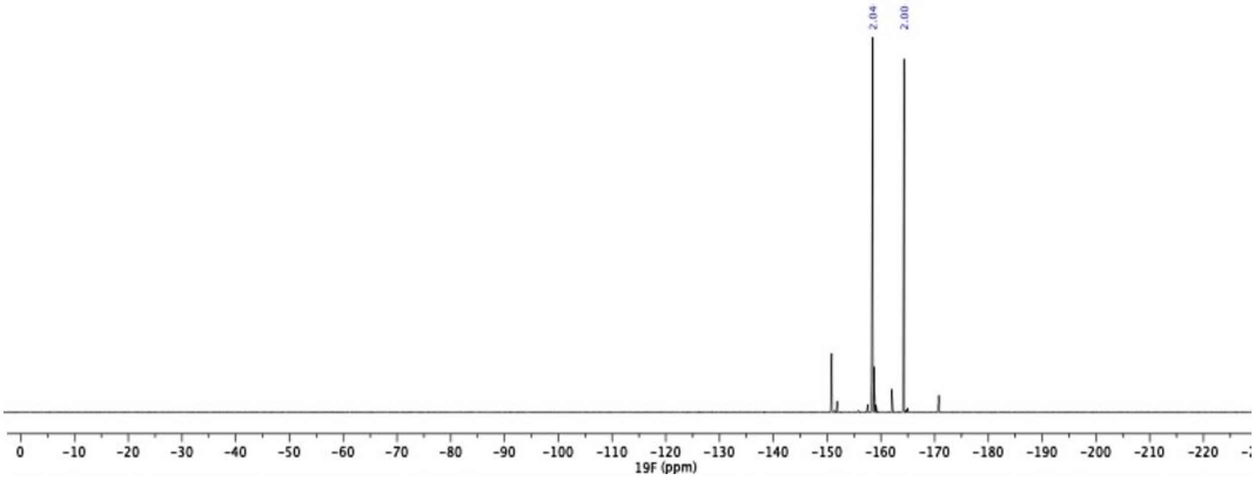

^1H NMR (500 MHz, Chloroform-*d*)

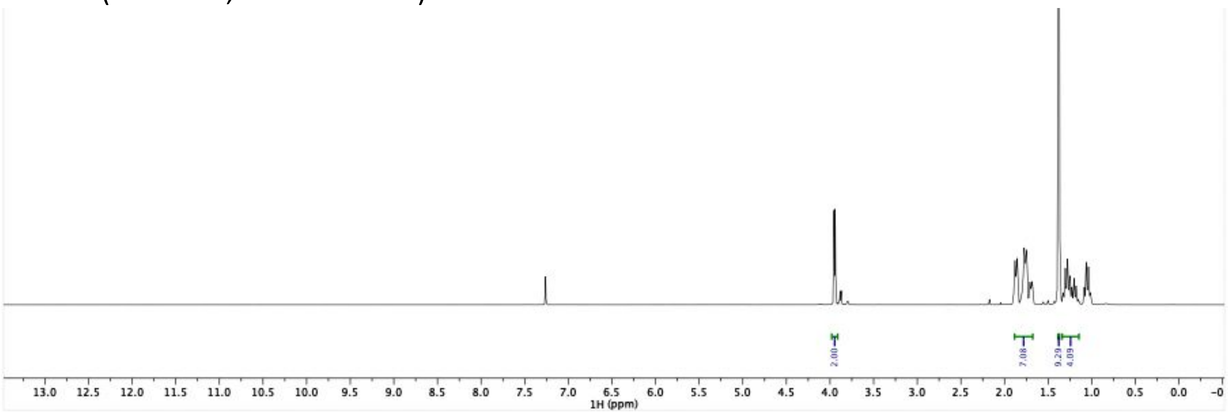

$^{13}C\{^1H\}$ NMR (126 MHz, Chloroform-*d*)

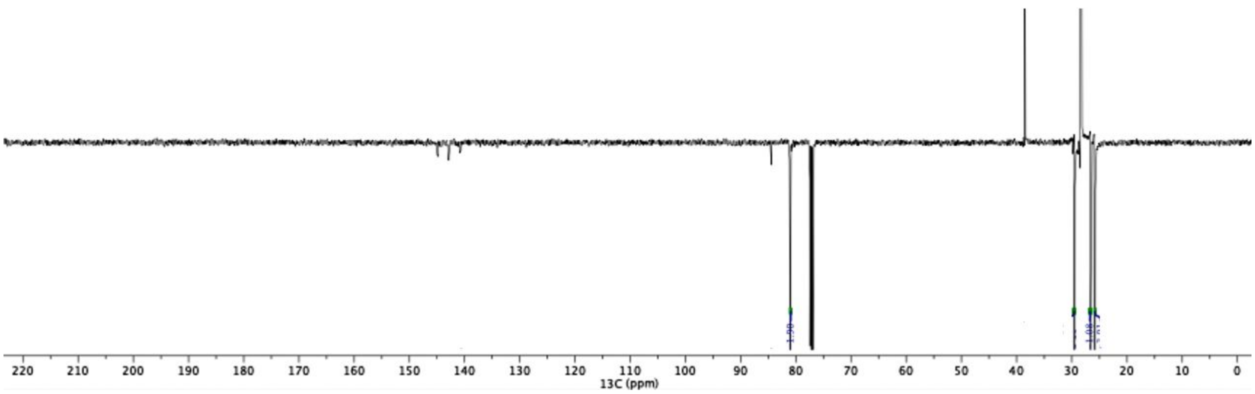

4-(cyclohexylmethoxy)-2,3,5,6-tetrafluorophenol 13a

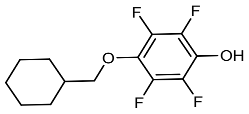

$^{19}F\{^{1}H\}$ NMR (377 MHz, Chloroform-*d*)

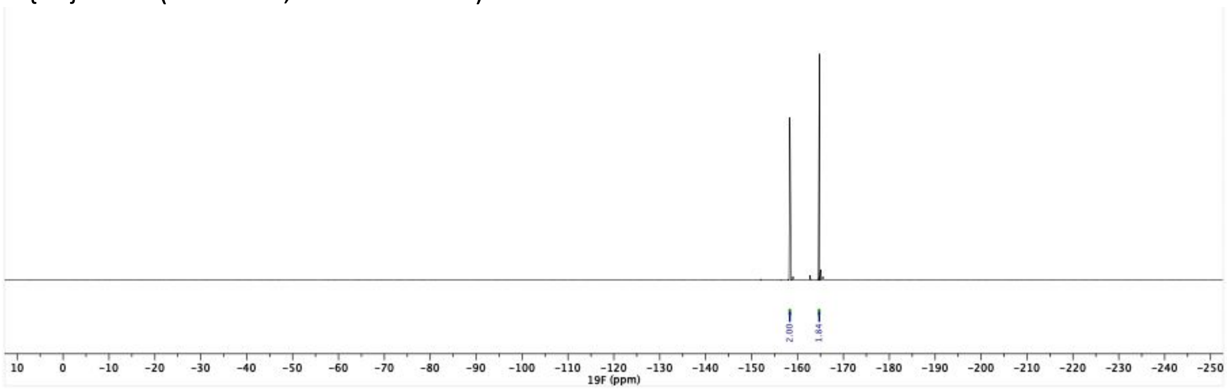

^{1}H NMR (500 MHz, Chloroform-*d*)

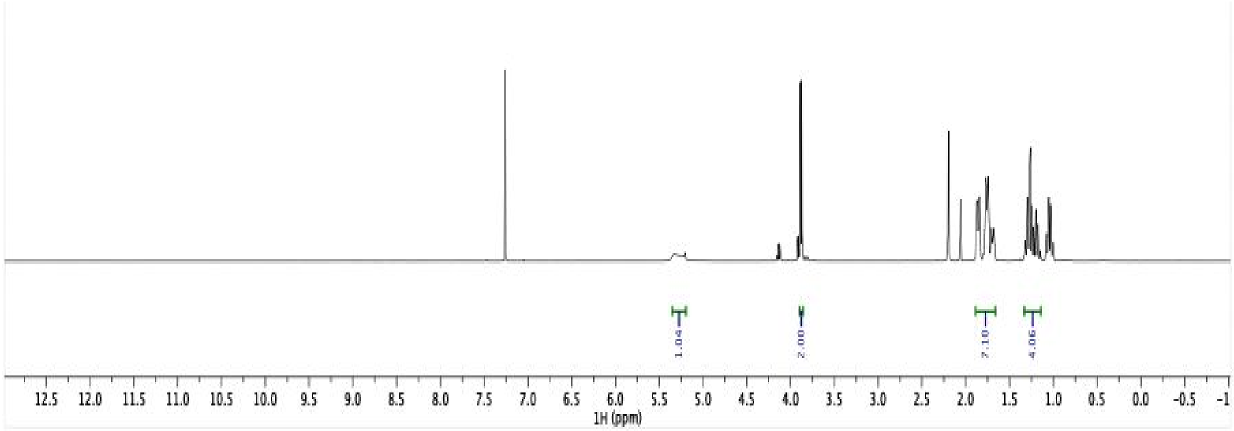

$^{13}C\{^{1}H\}$ NMR (126 MHz, Chloroform-*d*)

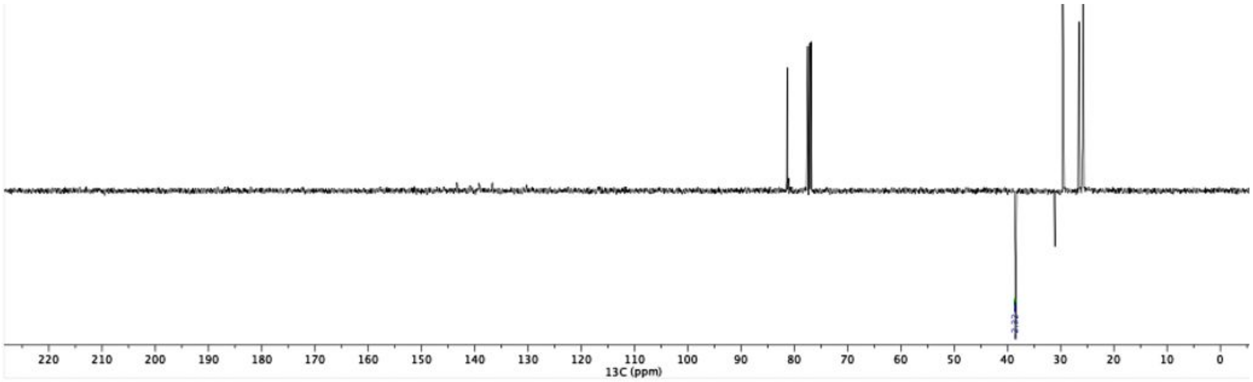
220 210 200 190 180 170 160 150 140 130 120 110 100 90 80 70 60 50 40 30 20 10 0
13C (ppm)

1-butoxy-4-(cyclohexylmethoxy)-2,3,5,6-tetrafluorobenzene 14a

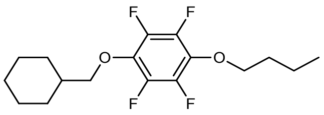

^{19}F NMR (470 MHz, Chloroform-*d*)

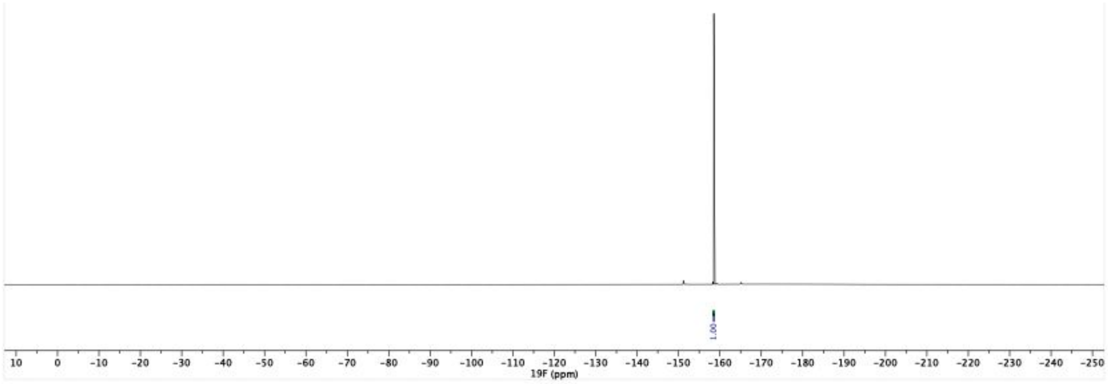

^{1}H NMR (500 MHz, Chloroform-*d*)

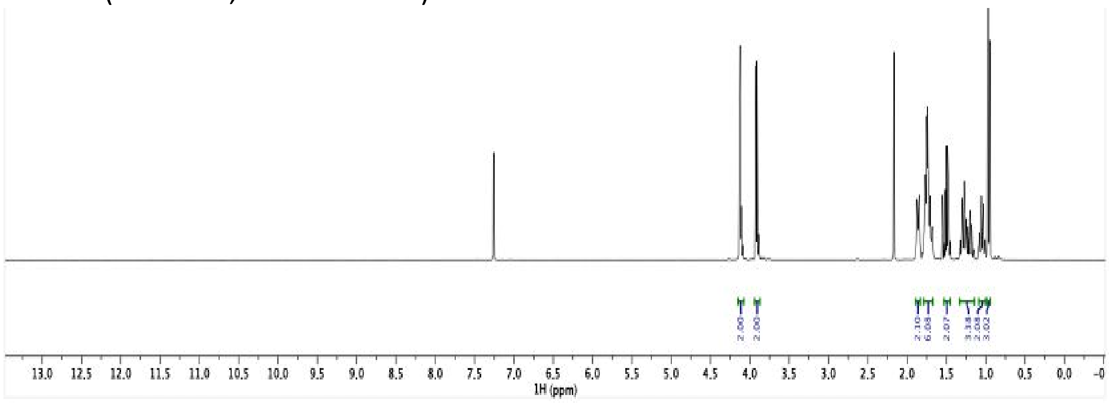

^{13}C{^{1}H} NMR (126 MHz, Chloroform-*d*)

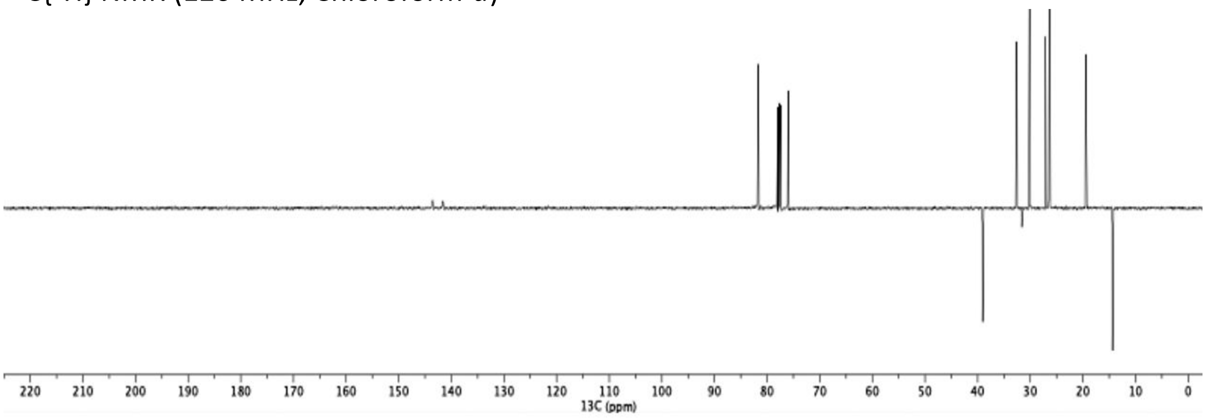

1-butoxy-4-(cyclohexylmethoxy)-2,3,5,6-tetrafluorocyclohexane 1a

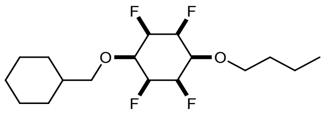

$^{19}F\{^{1}H\}$ NMR (470 MHz, Chloroform-*d*)

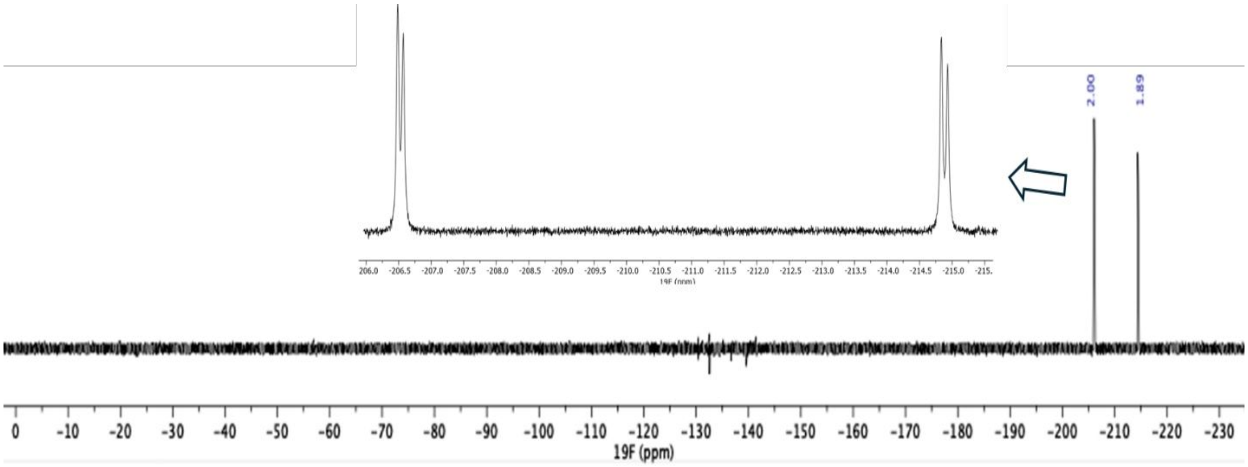

^{1}H NMR (500 MHz, Chloroform-*d*)

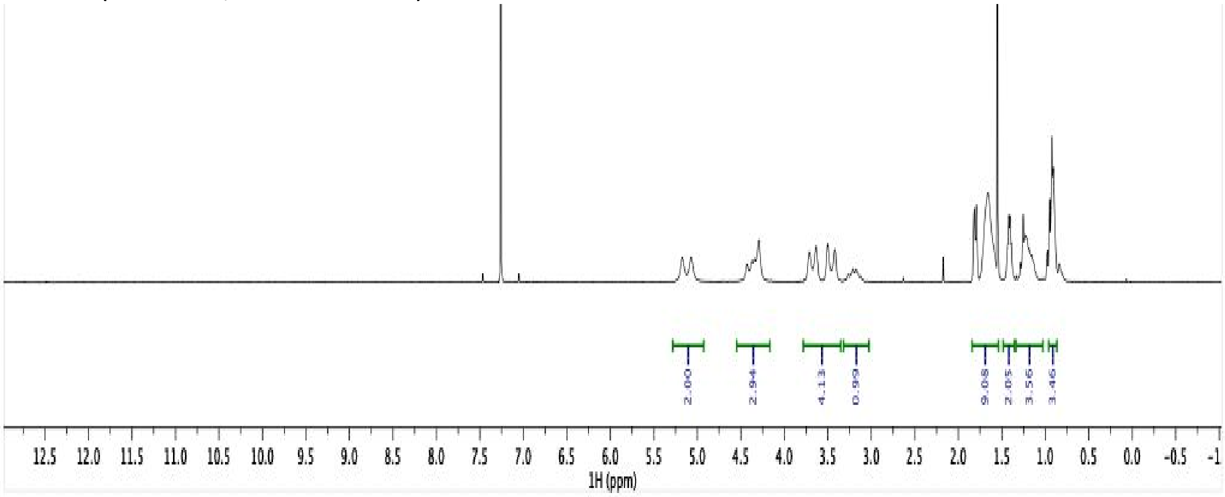

(4-(*tert*-butoxy)-2,3,5,6-tetrafluorophenoxy)-4'-propyl-1,1'-bi(cyclohexane) 12b

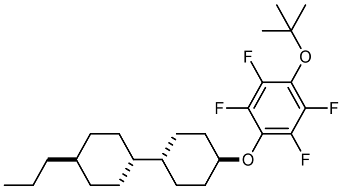

^{19}F{^{1}H} NMR (470 MHz, Chloroform-*d*)

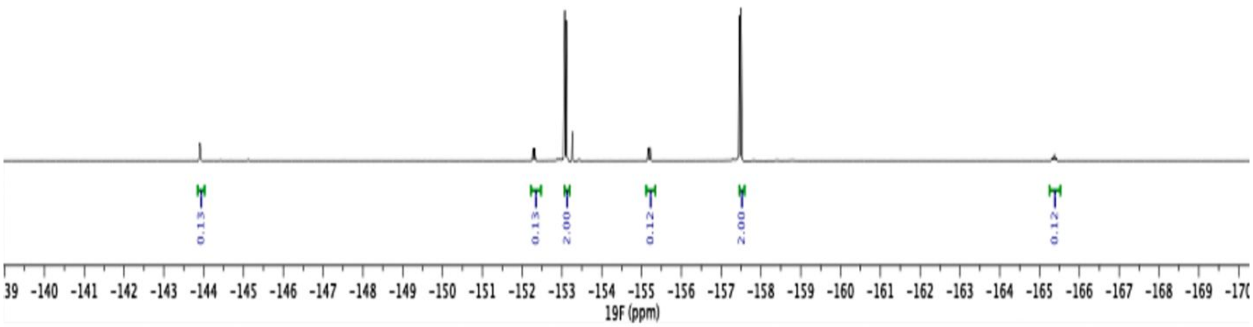

^{1}H NMR (500 MHz, Chloroform-*d*)

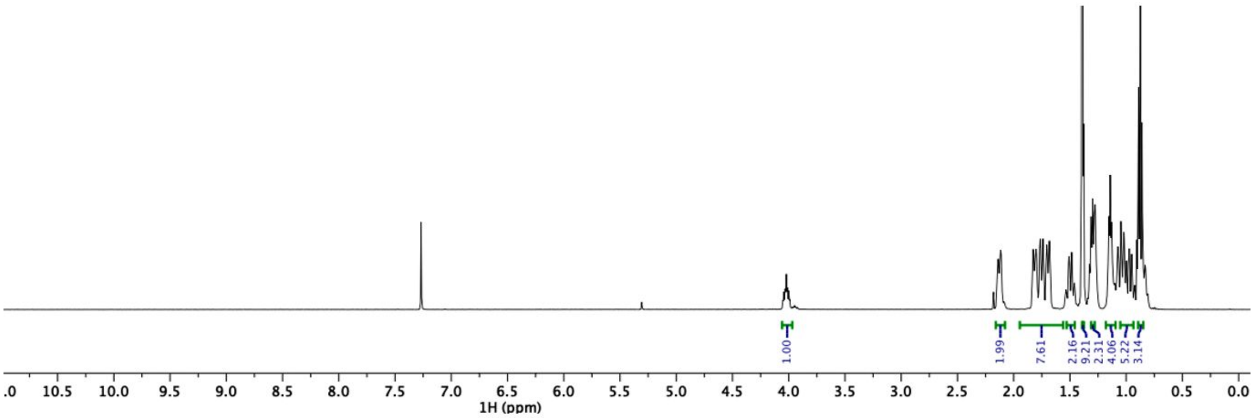

^{13}C{^{1}H} NMR (126 MHz, Chloroform-*d*)

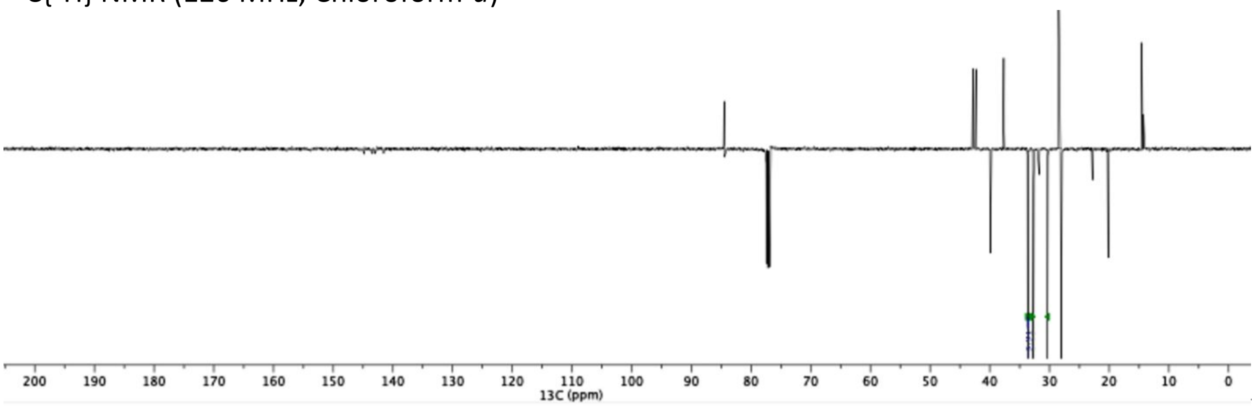

2,3,5,6-tetrafluoro-4'-propyl-[1,1'-bi(cyclohexan)]-4-yl)oxy)phenol) 13b

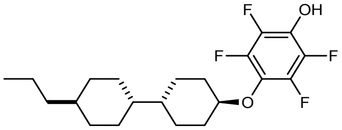

$^{19}F\{^{1}H\}$ NMR (470 MHz, Chloroform-*d*)

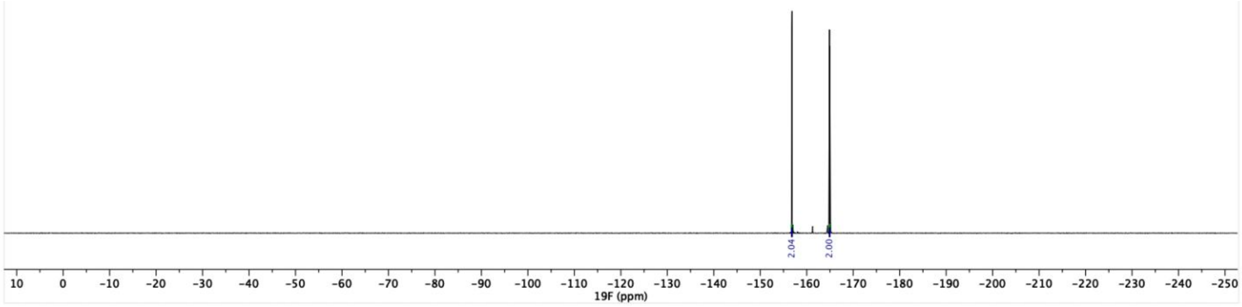

^{1}H NMR (400 MHz, Chloroform-*d*)

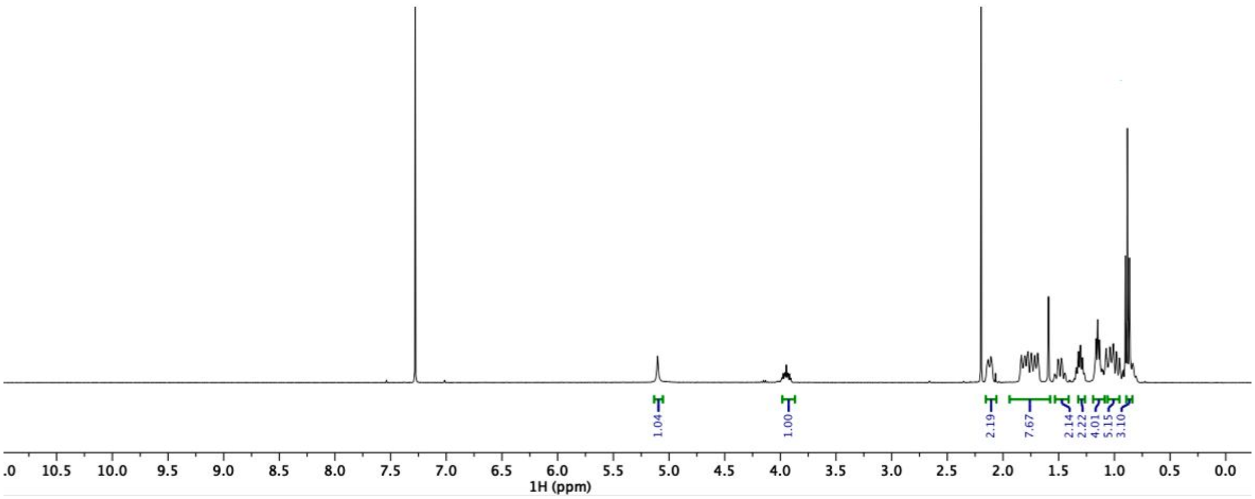

$^{13}C\{^{1}H\}$ NMR (101 MHz, Chloroform-*d*)

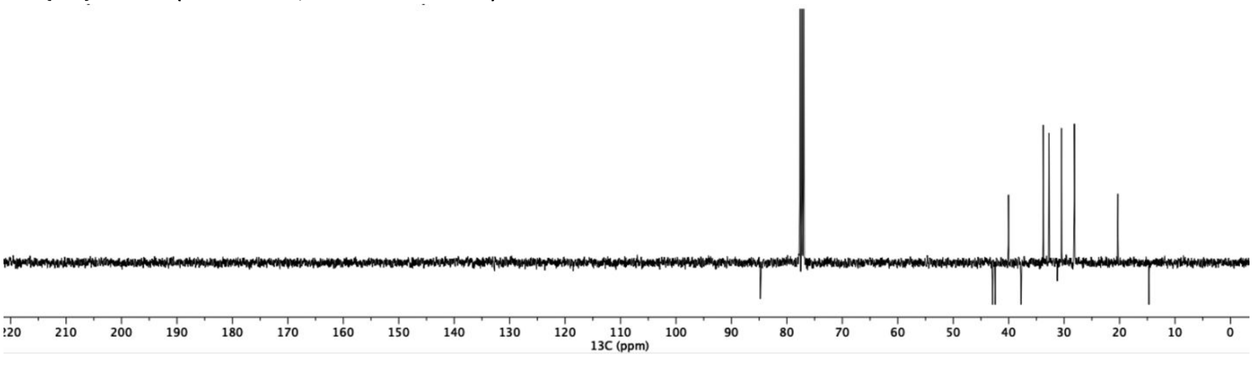

4-(4-butoxy-2,3,5,6-tetrafluorophenoxy)-4'-propyl-1,1'-bi(cyclohexane) 14b

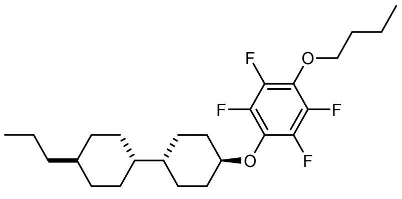

$^{19}F\{^{1}H\}$ NMR (470 MHz, Chloroform-*d*)

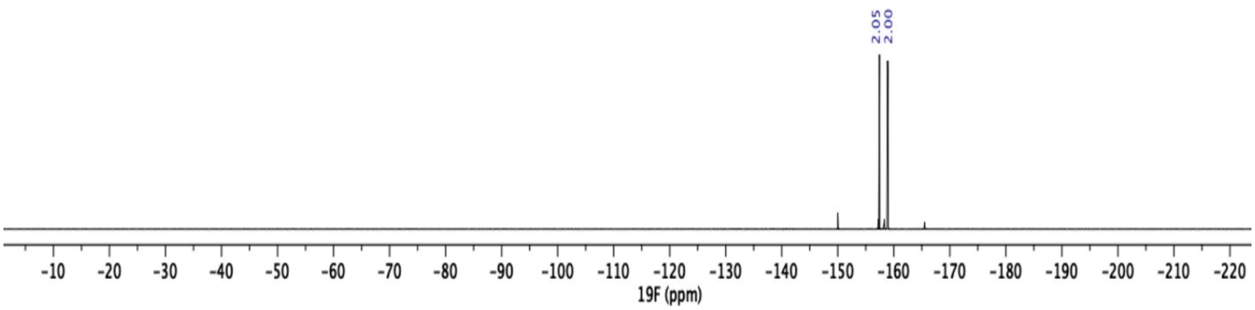

^{1}H NMR (500 MHz, Chloroform-*d*)

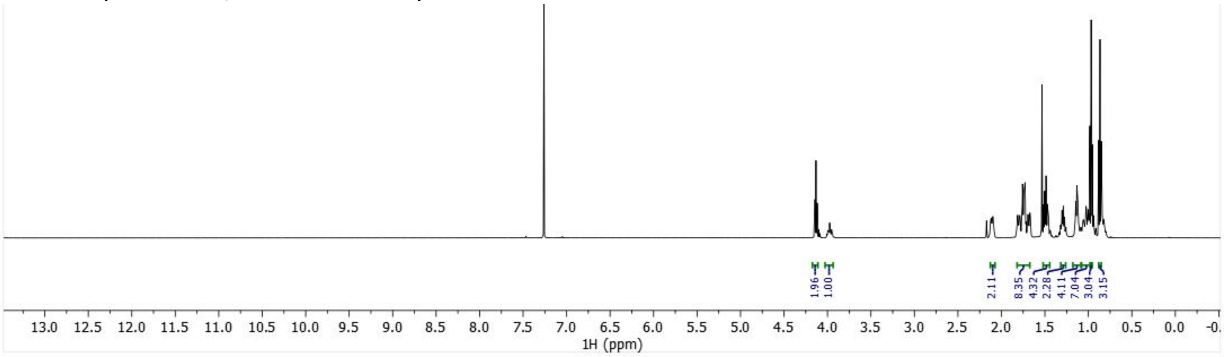

$^{13}C\{^{1}H\}$ NMR (126 MHz, Chloroform-*d*)

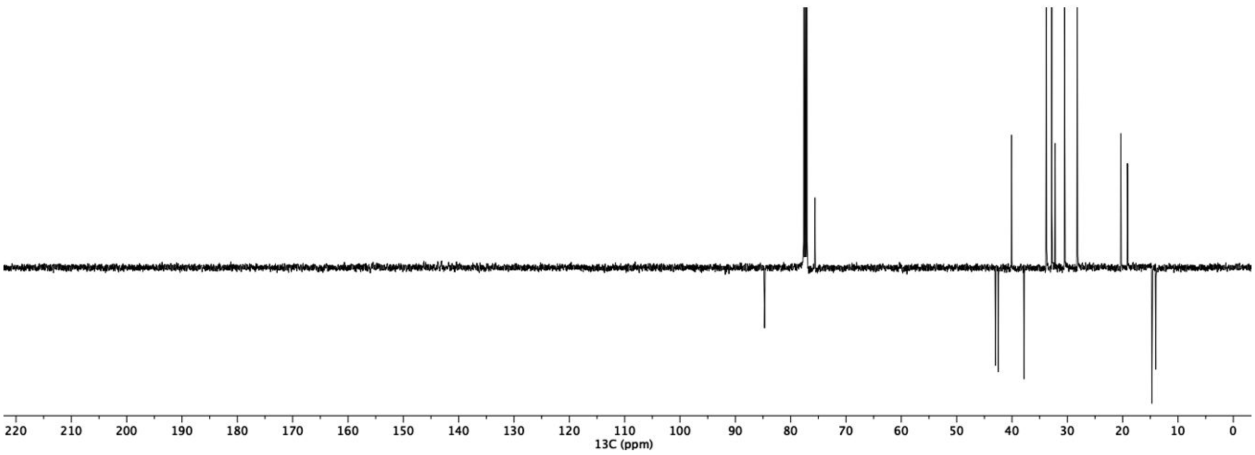

4-butoxy-2,3,5,6-tetrafluorohexyl)oxy)-4’-propyl-1,1’-bi(cyclohexane) 1b

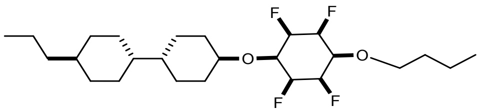

$^{19}F\{^{1}H\}$ NMR (376 MHz, Chloroform-*d*)

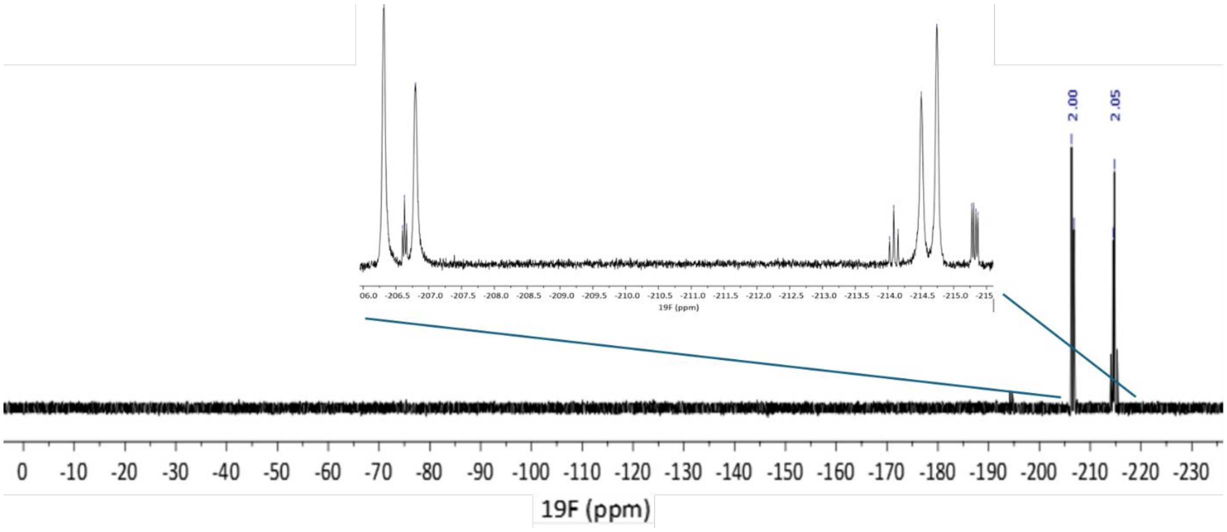

^{1}H NMR (400 MHz, Chloroform-*d*)

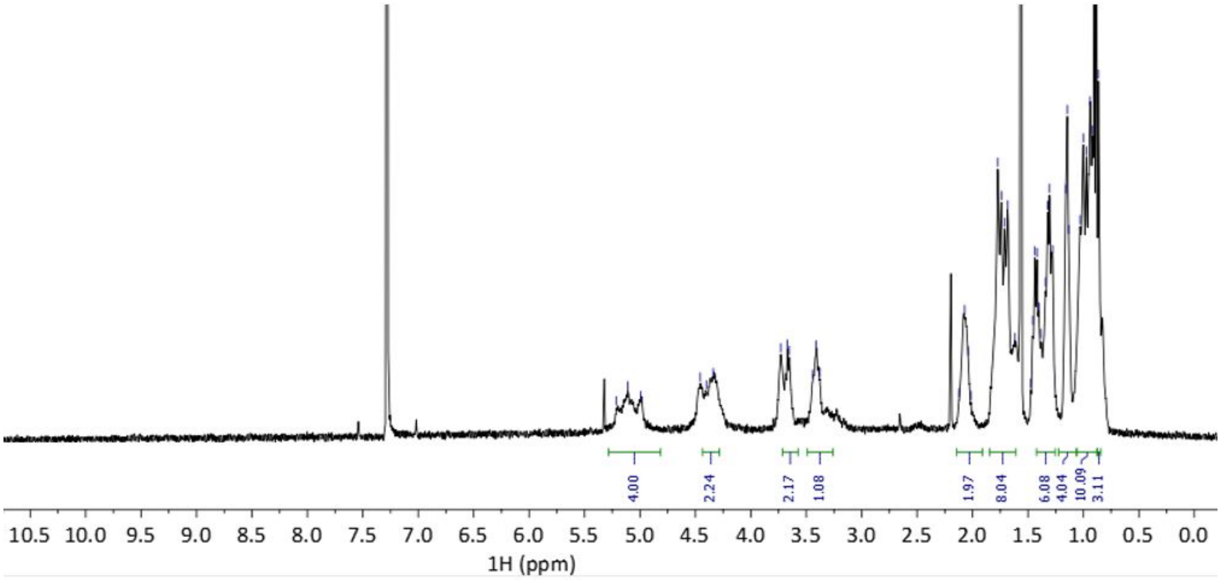

$^{13}C\{^{1}H\}$ NMR (101 MHz, Chloroform-*d*)

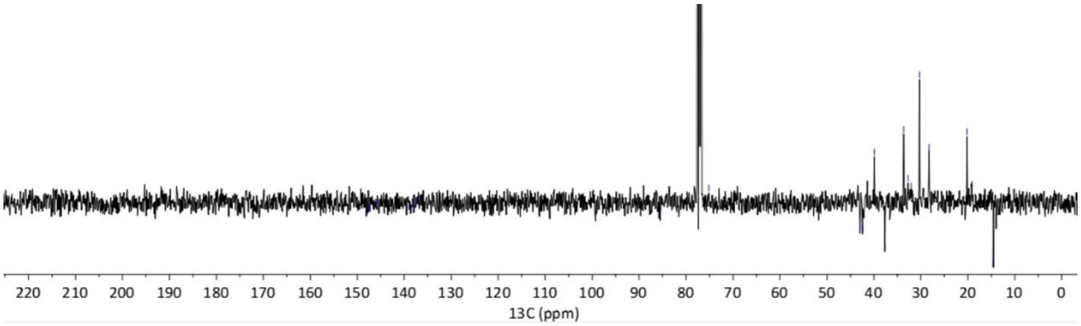
220 210 200 190 180 170 160 150 140 130 120 110 100 90 80 70 60 50 40 30 20 10 0
13C (ppm)

tert-butoxy-2,3,5,6-tetrafluorophenoxy-4-methylene-bicyclohexane 12c

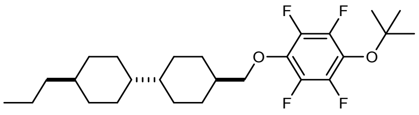

$^{19}F\{^1H\}$ NMR (470 MHz, Chloroform-*d*)

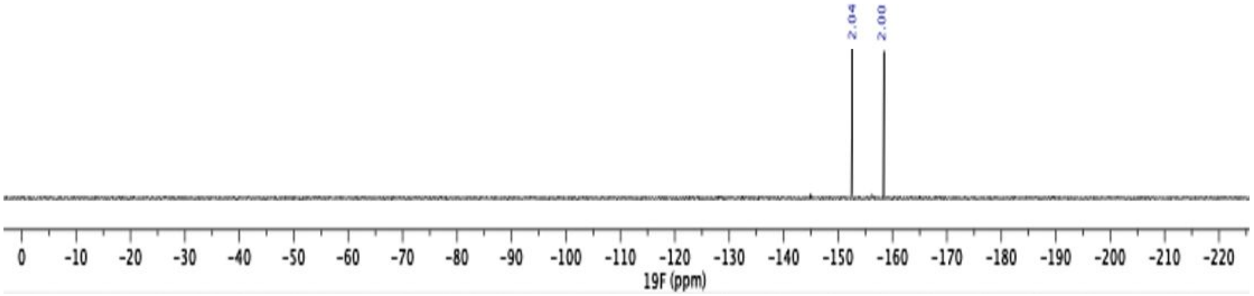

^1H NMR (500 MHz, Chloroform-*d*)

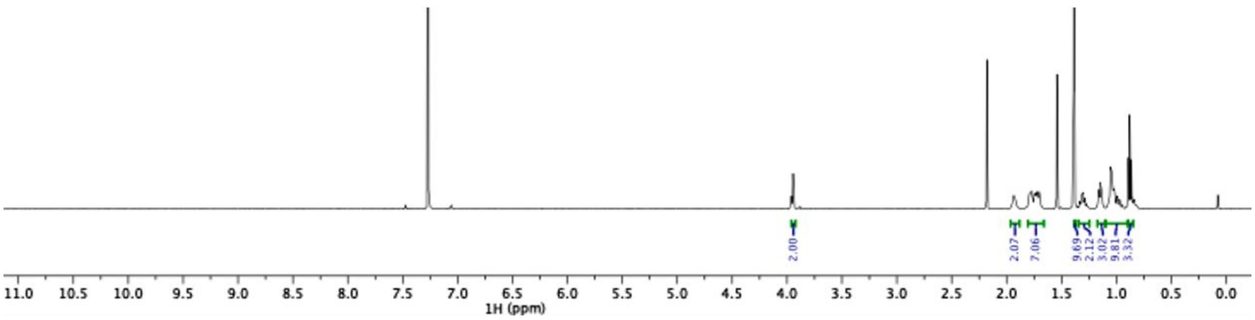

$^{13}C\{^1H\}$ NMR (126 MHz, Chloroform-*d*)

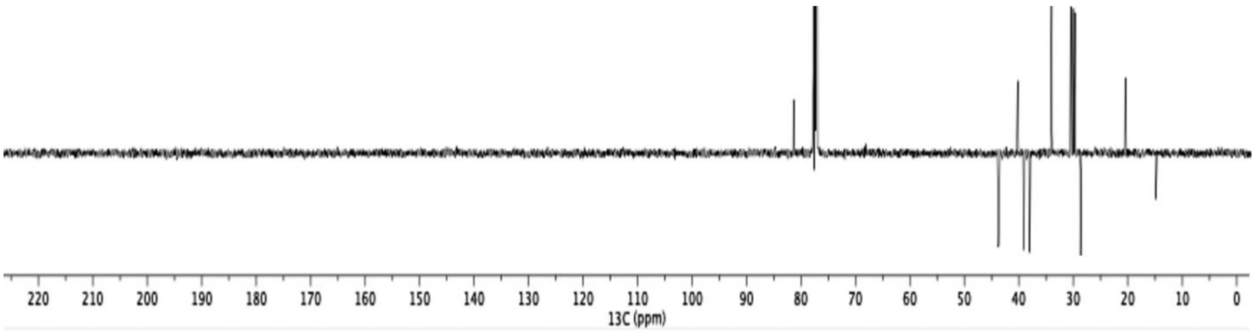

2,3,5,6-tetrafluorophenol-4'- methylene-bicyclohexane - 13c

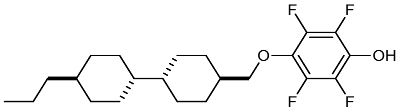

$^{19}F\{^1H\}$ NMR (470 MHz, Chloroform-*d*)

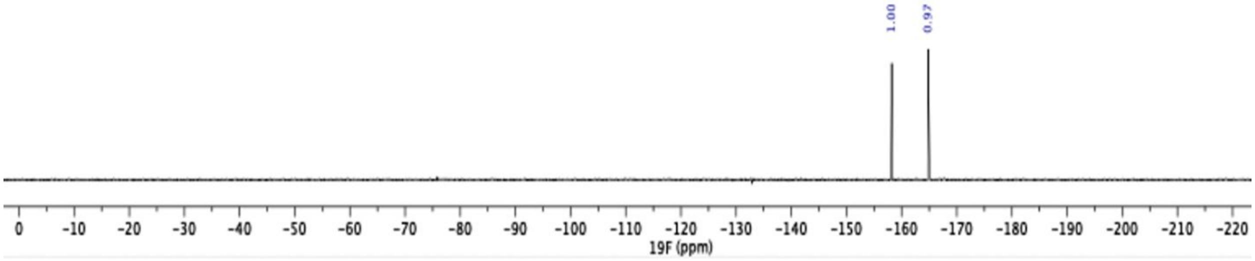

^1H NMR (500 MHz, Chloroform-*d*)

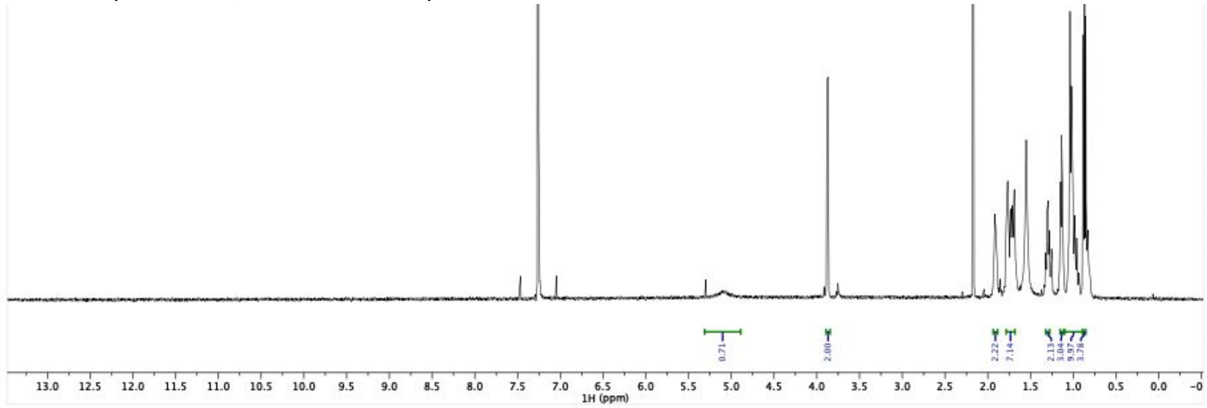

$^{13}C\{^1H\}$ NMR (126 MHz, Chloroform-*d*)

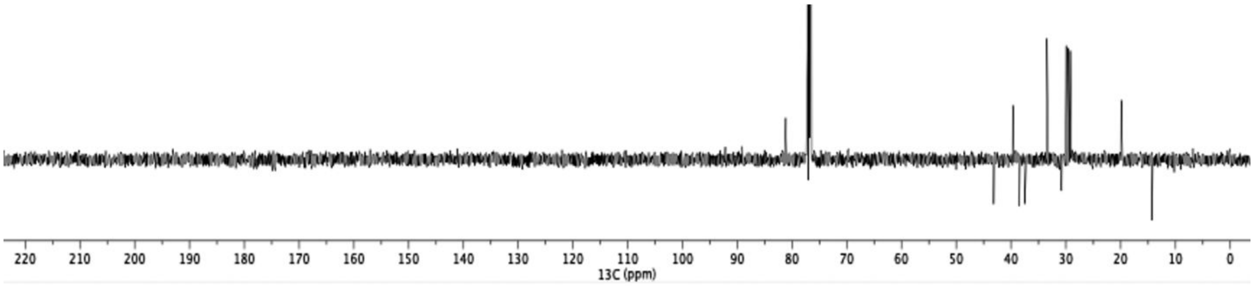

4-*n*-butoxy-2,3,5,6-tetrafluorophenoxy-4-methylene-4'-propyl bicyclohexane 14c

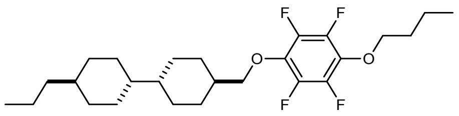

$^{19}F\{^{1}H\}$ NMR (470 MHz, Chloroform-*d*)

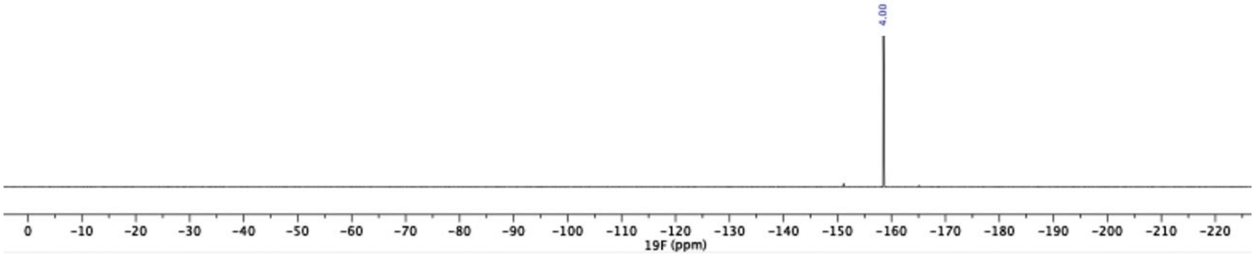

^{1}H NMR (500 MHz, Chloroform-*d*)

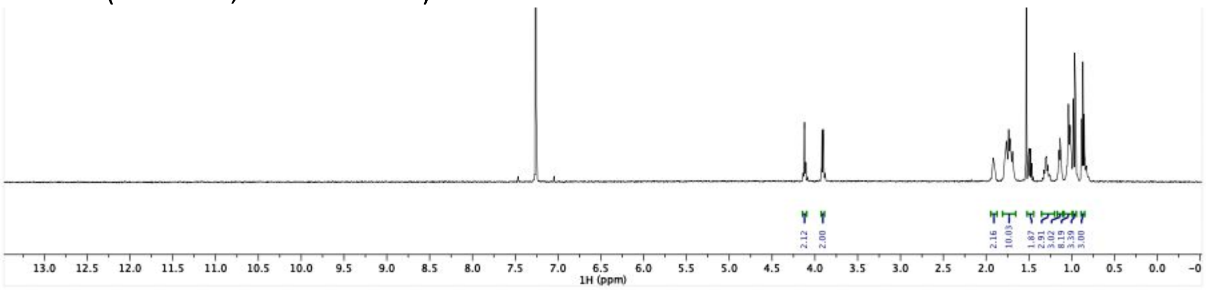

$^{13}C\{^{1}H\}$ NMR (126 MHz, Chloroform-*d*)

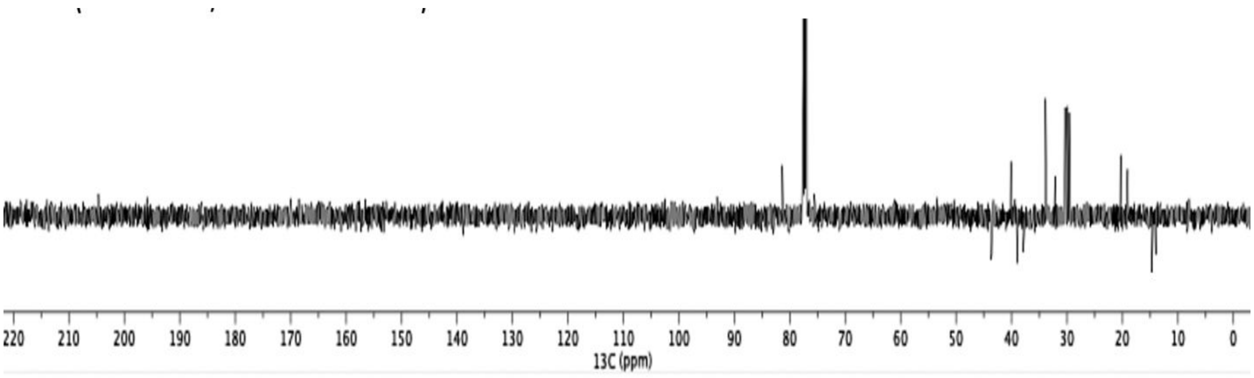

4-*n*-butoxy-2,3,5,6-tetrafluorocyclohexyloxy-4-methylene-4'-propyl bicyclohexane 1c

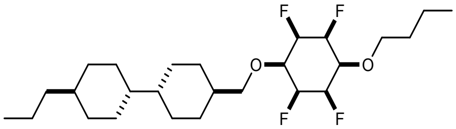

$^{19}F\{^{1}H\}$ NMR (470 MHz, Chloroform-*d*)

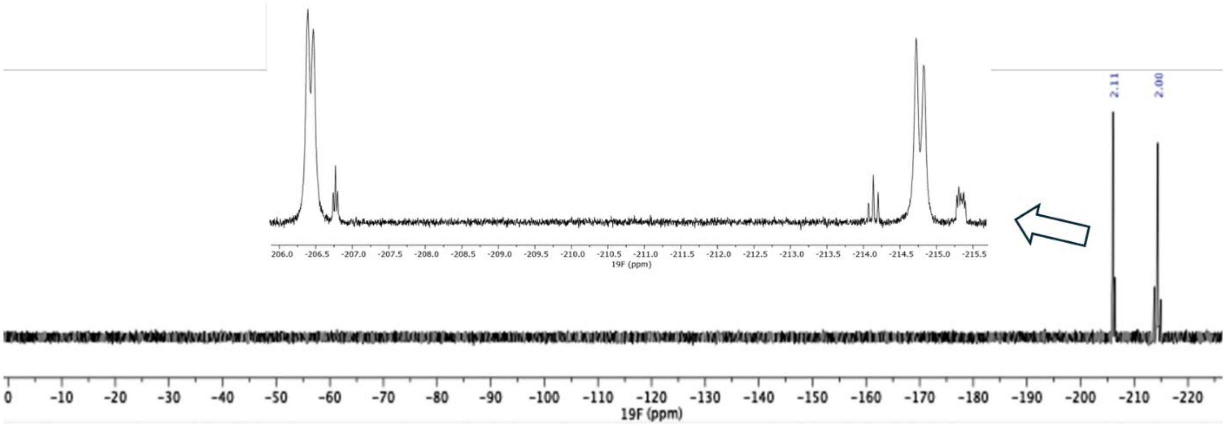

^{1}H NMR (500 MHz, Chloroform-*d*)

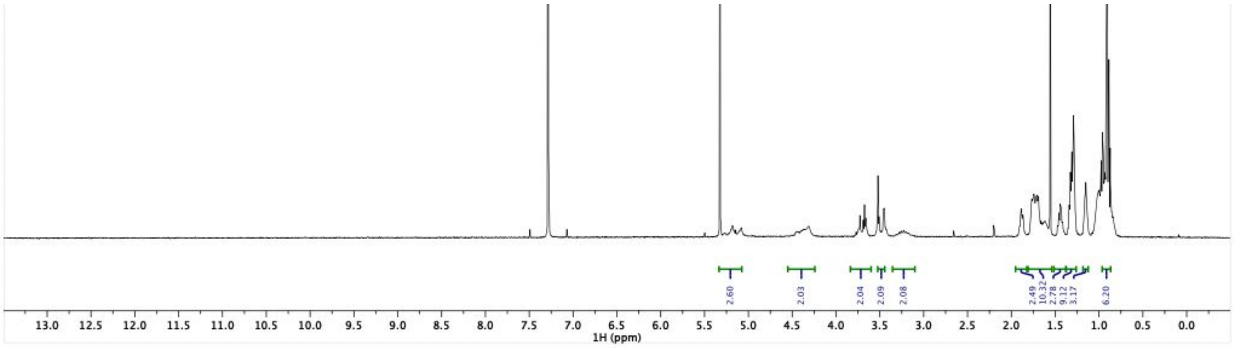

$^{13}C\{^{1}H\}$ NMR (126 MHz, Chloroform-*d*)

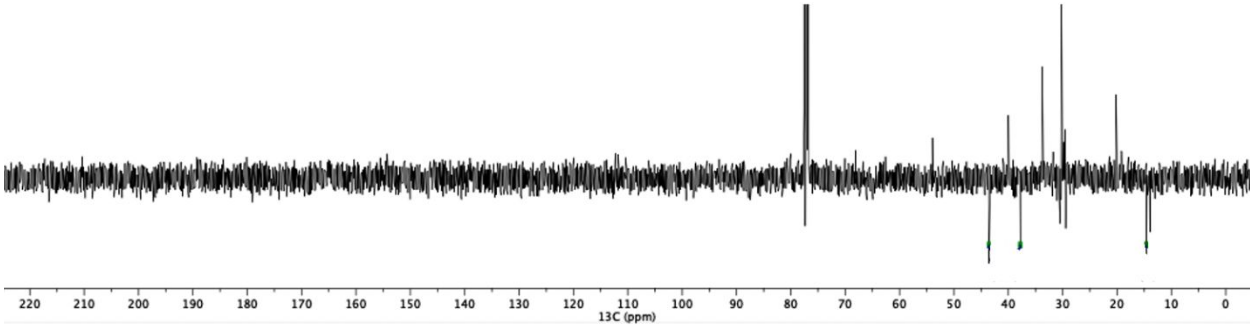

1-(*tert*-butoxy)-2,3,5,6-tetrafluoro-4-benzene - (1*R*,2*S*,5*R*)-menthyl diether 12d

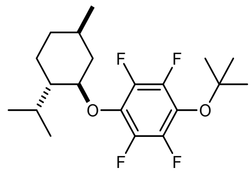

^{19}F{^{1}H} NMR (470 MHz, Chloroform-*d*)

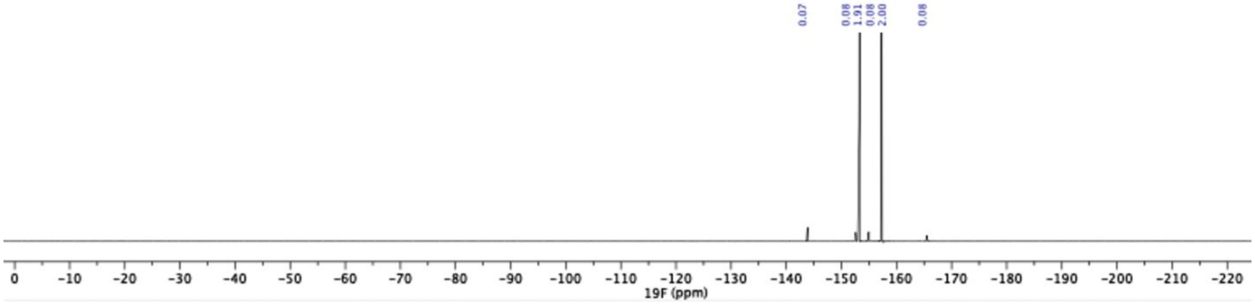

^{1}H NMR (500 MHz, Chloroform-*d*)

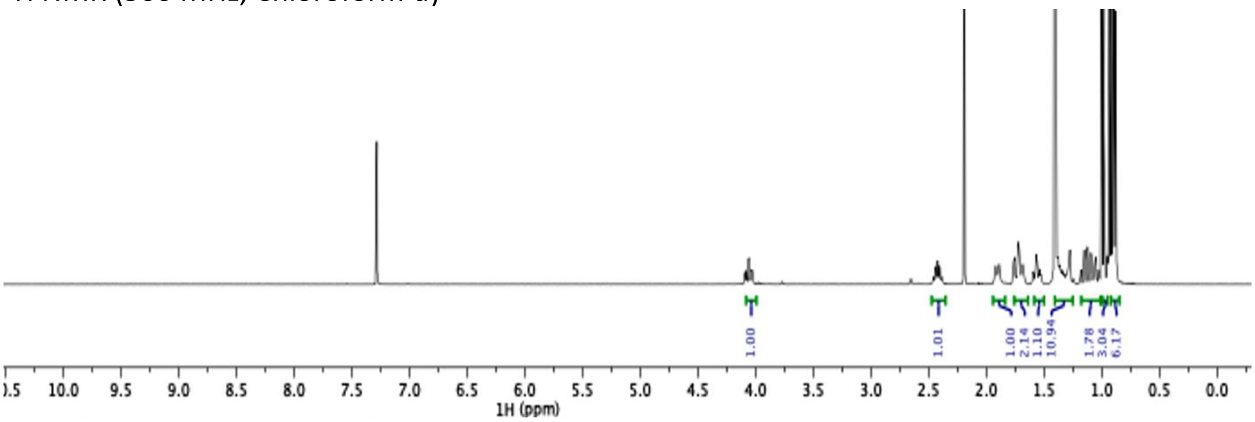

^{13}C{^{1}H} NMR (126 MHz, Chloroform-*d*)

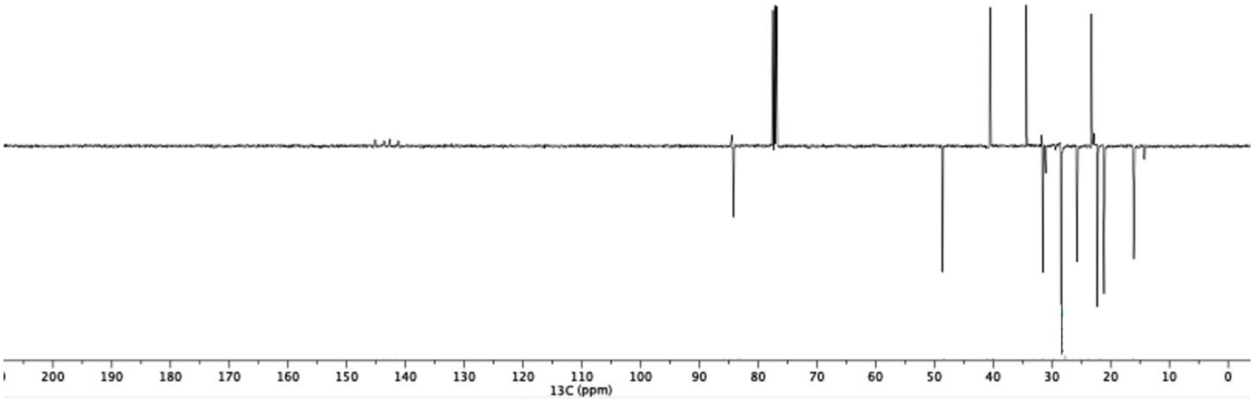

2,3,5,6-tetrafluorophenol -4- (1*R*,2*S*,5*R*)-menthyl ether 13d

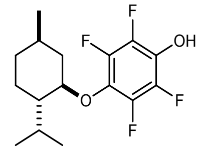

$^{19}F\{^{1}H\}$ NMR (470 MHz, Chloroform-*d*)

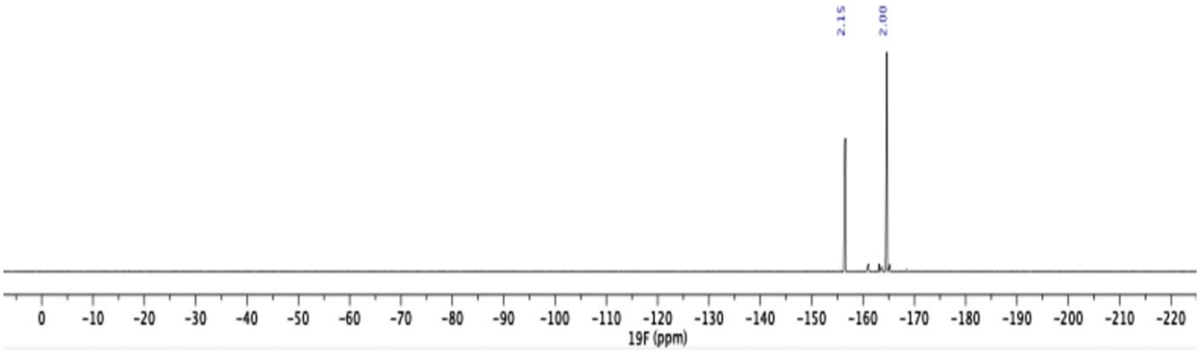

^{1}H NMR (500 MHz, Chloroform-*d*)

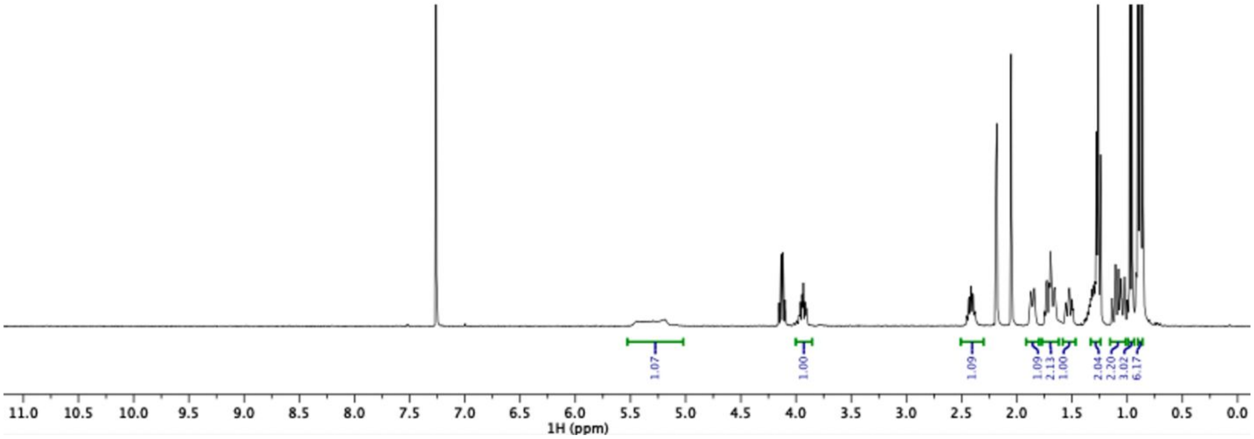

$^{13}C\{^{1}H\}$ NMR (101 MHz, Chloroform-*d*)

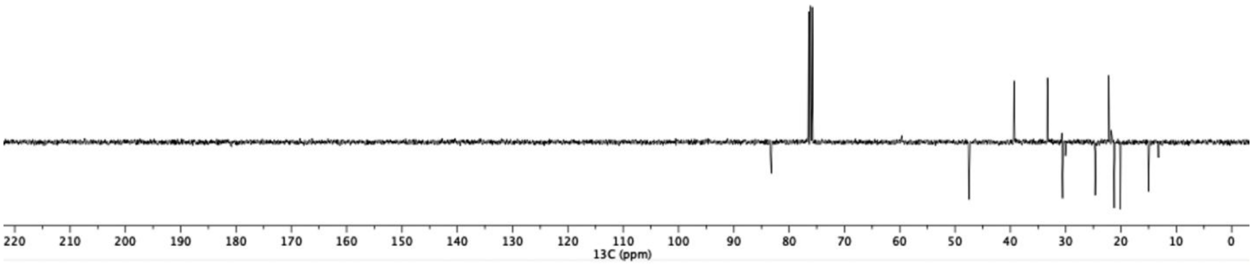

1-(*tert*-butoxy)-2,3,5,6-tetrafluoro-4-benzene - (1*R*,2*S*,5*R*)-menthyl diether 14d

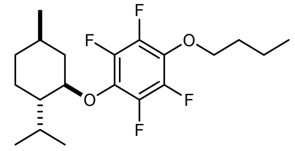

$^{19}F\{^{1}H\}$ NMR (377 MHz, Chloroform-*d*)

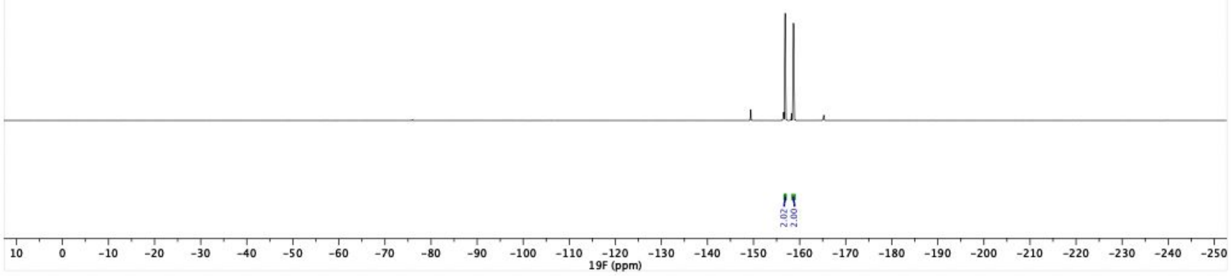

^{1}H NMR (400 MHz, Chloroform-*d*)

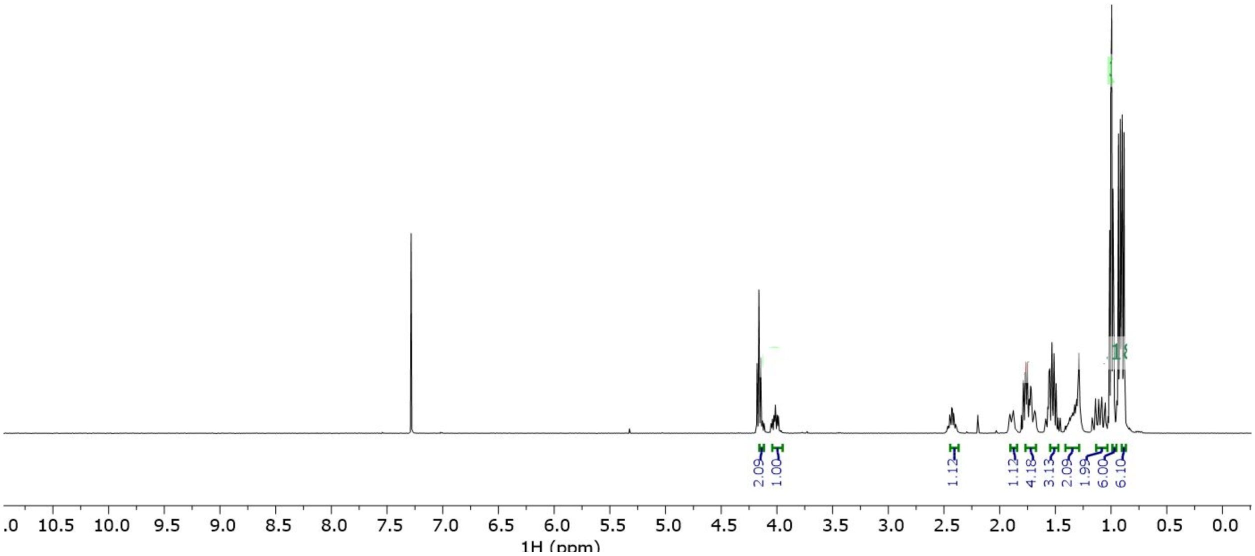

$^{13}C\{^{1}H\}$ NMR (101 MHz, Chloroform-*d*)

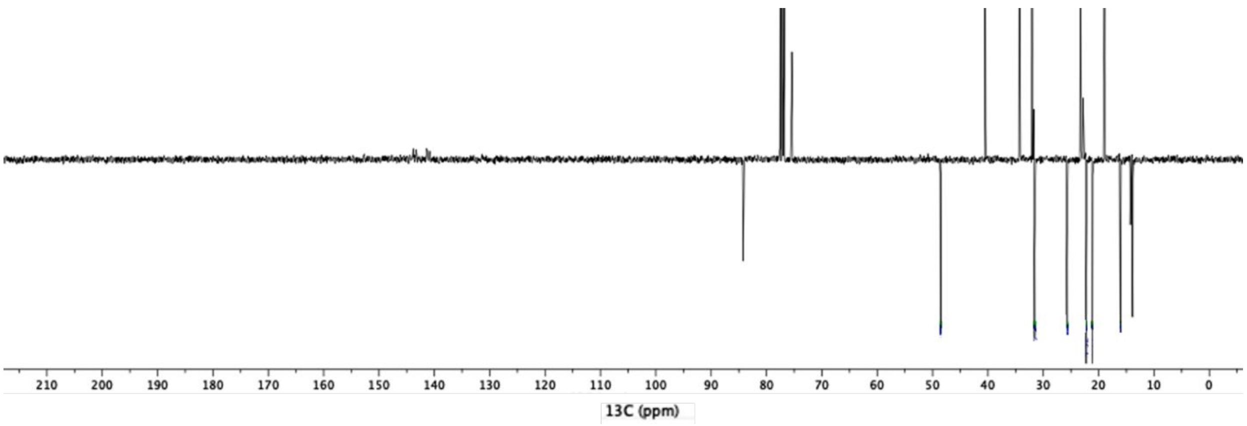
210 200 190 180 170 160 150 140 130 120 110 100 90 80 70 60 50 40 30 20 10 0
13C (ppm)

1-(*n*-butyl)-all-*cis* 2,3,5,6-tetrafluorocyclohexane-4 -(1*R*,2*S*,5*R*)-menthyl diether 1d

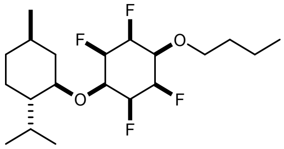

$^{19}F\{^{1}H\}$ NMR (377 MHz, Chloroform-*d*)

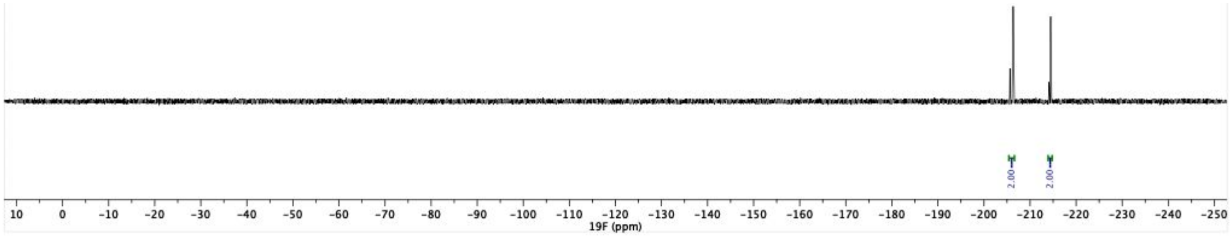

^{1}H NMR (500 MHz, Chloroform-*d*)

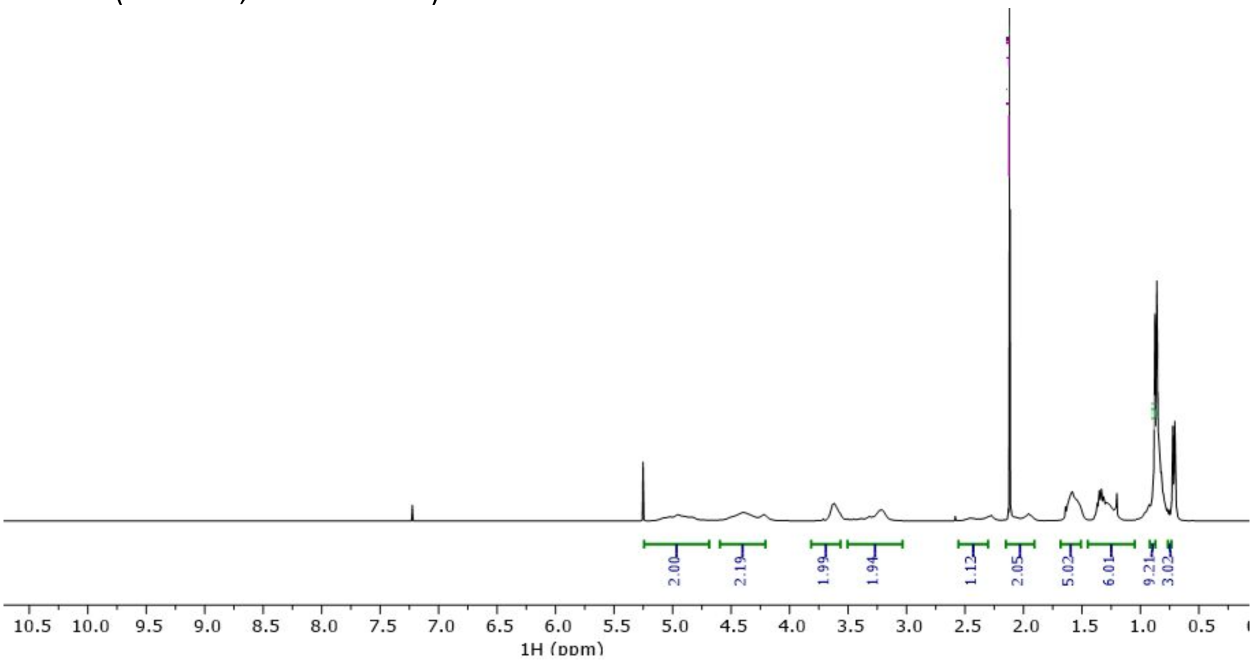

$^{13}C\{^{1}H\}$ NMR (101 MHz, Chloroform-*d*) **1d**

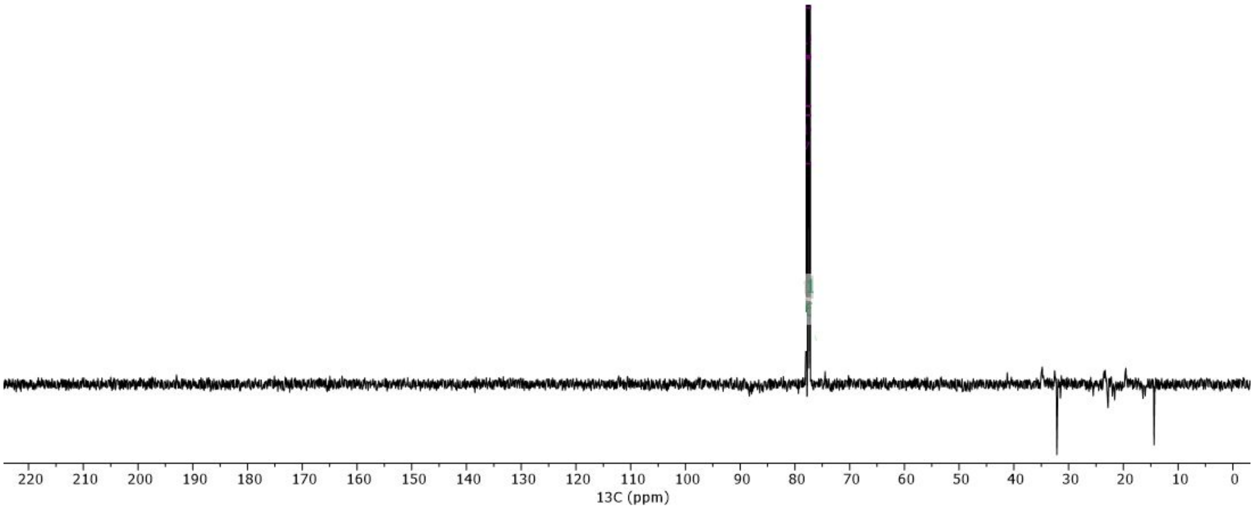

4-methoxyl-2,3,5,6-tetrafluorophenoxy-4-methylene-4’-propylbicyclohexane ether – 14e

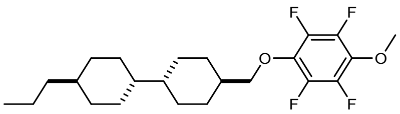

^{19}F{^{1}H} NMR (470 MHz, Chloroform-*d*)

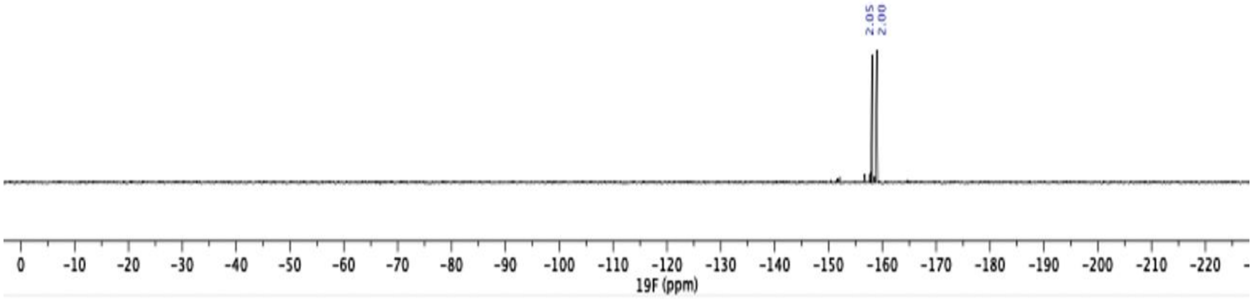

^{1}H NMR (500 MHz, Chloroform-*d*)

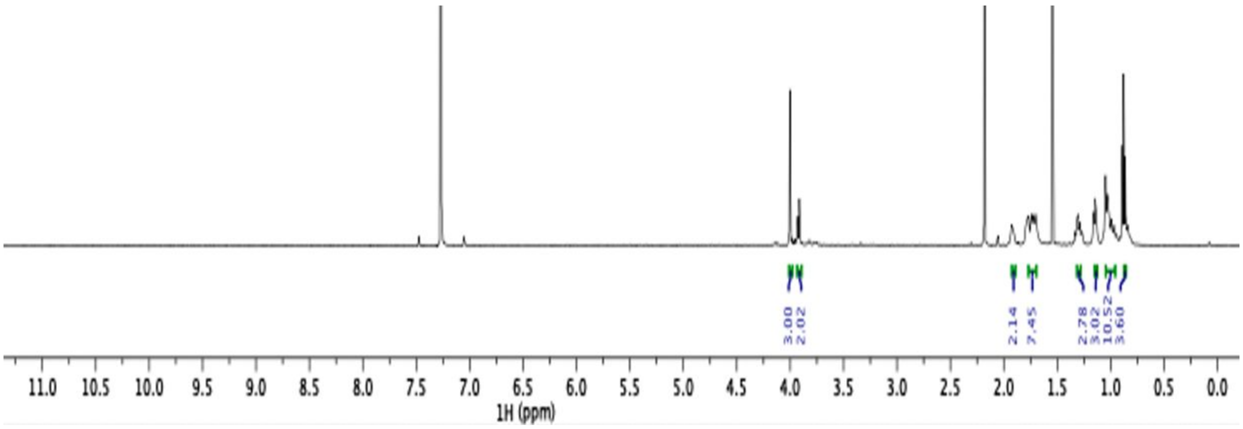

^{13}C{^{1}H} NMR (101 MHz, Chloroform-*d*)

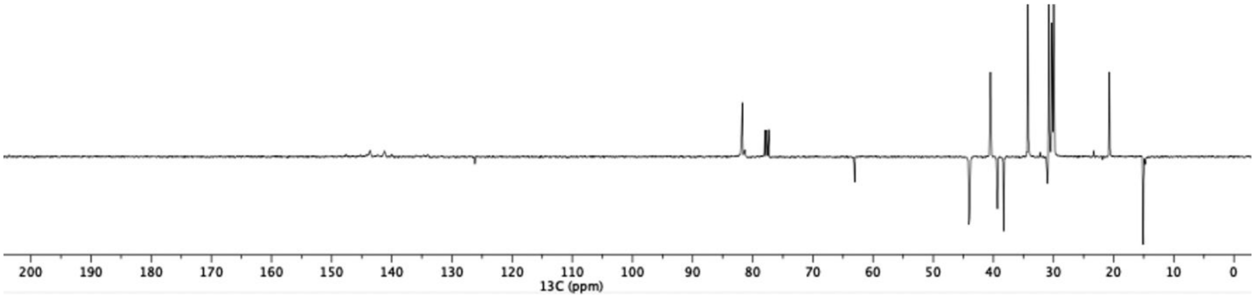

All-*cis* 4-methoxyl-2,3,5,6- tetrafluorocyclohexyl-1-methyleneoxy-(4'-propyl)bicyclohexyl ether 1e

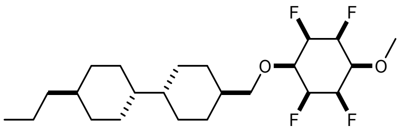

$^{19}F\{^{1}H\}$ NMR (470 MHz, Chloroform-*d*)

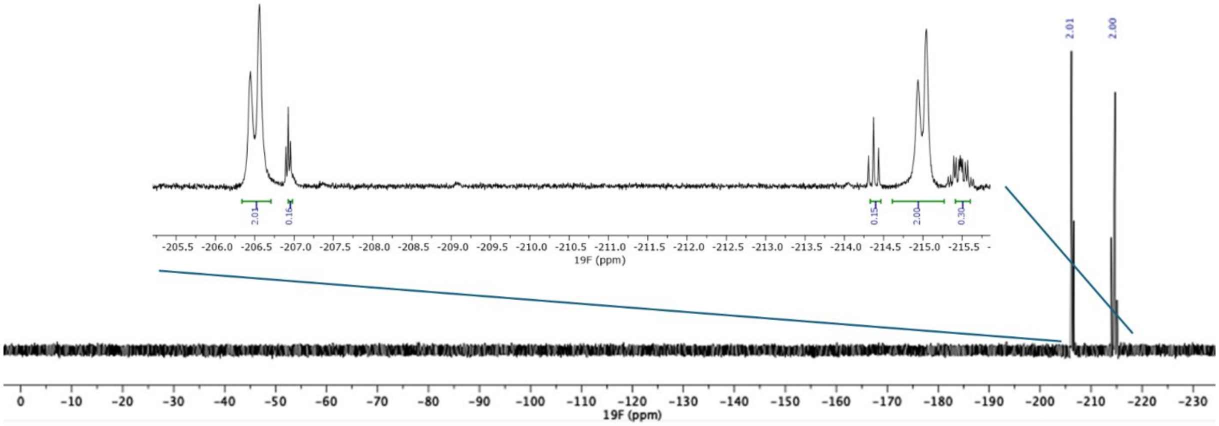

^{1}H NMR (400 MHz, Chloroform-*d*)

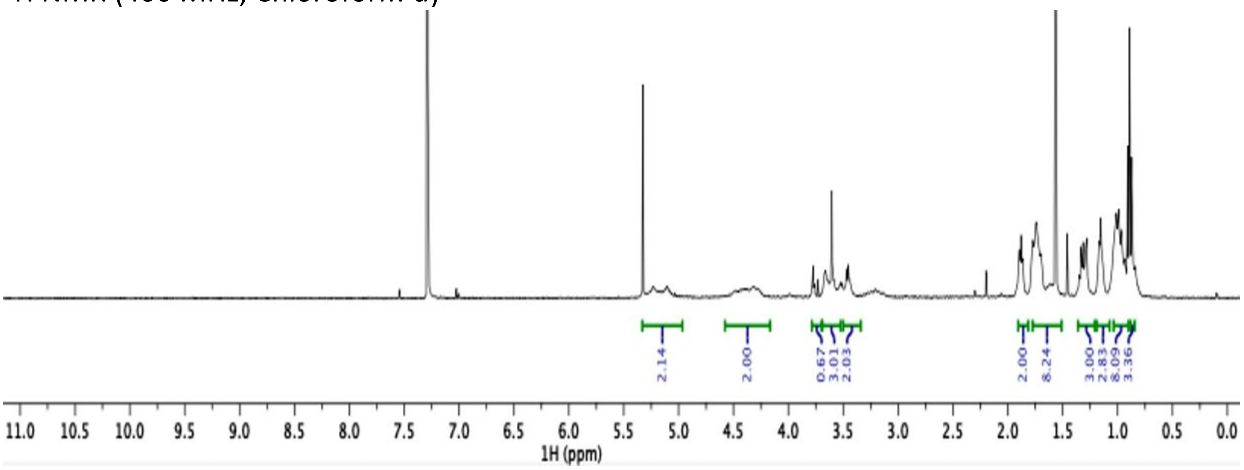

$^{13}C\{^{1}H\}$ NMR (101 MHz, Chloroform-*d*)

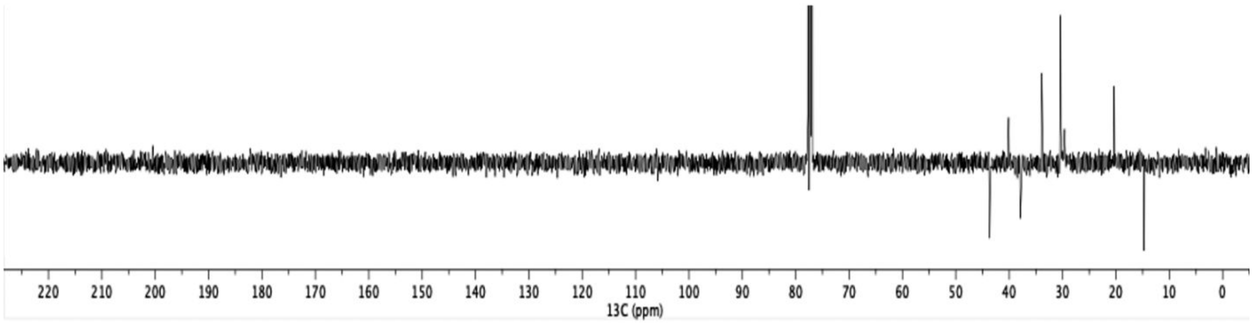
220
210
200
190
180
170
160
150
140
130
120
110
100
90
80
70
60
50
40
30
20
10
0
13C (ppm)

5. Differential Scanning Calorimetry (DSC) Profiles

Thermophysical studies

The phase transition behaviours of the compounds were examined by Polarising Optical Microscopy (POM) using a BX53 microscope (Olympus, Tokyo, Japan) equipped with a heating–cooling stage (10.002 L, Linkam Scientific Instruments, Redhill, UK). The phase sequences and transition enthalpies were determined by DSC using a DSC-60 (Shimadzu, Kyoto, Japan) at heating and cooling rates of 5.0 °C min^{-1} in a N_2 atmosphere. Weight loss versus temperature was measured by thermogravimetric analysis (TGA) using a TGA-50 instrument (Shimadzu, Kyoto, Japan).

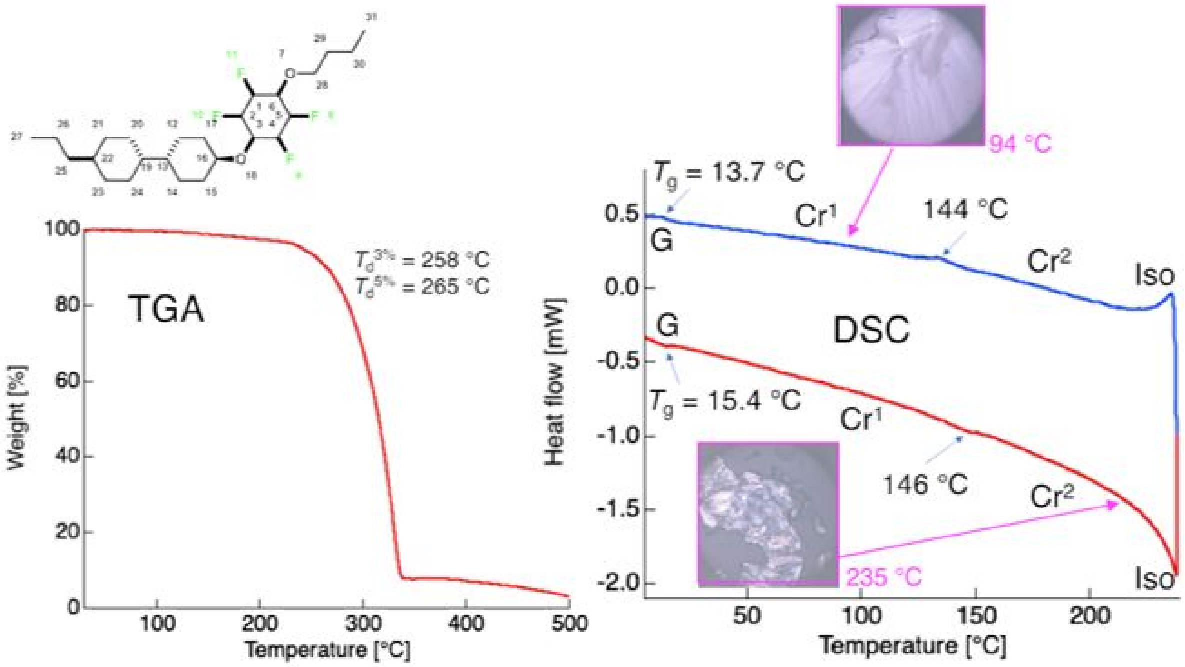

Figure S1 (Left) TGA thermogram of **1b** under N_2 atmosphere (5 °C min^{-1}). (Right) DSC thermogram of 1b during the second heating (red line) and cooling (blue line) processes under N_2 atmosphere. Inset: POM images at 235 °C on heating and 94 °C on cooling process. Abbreviations: G: glassy, Cr: crystalline, Iso: isotropic phases.

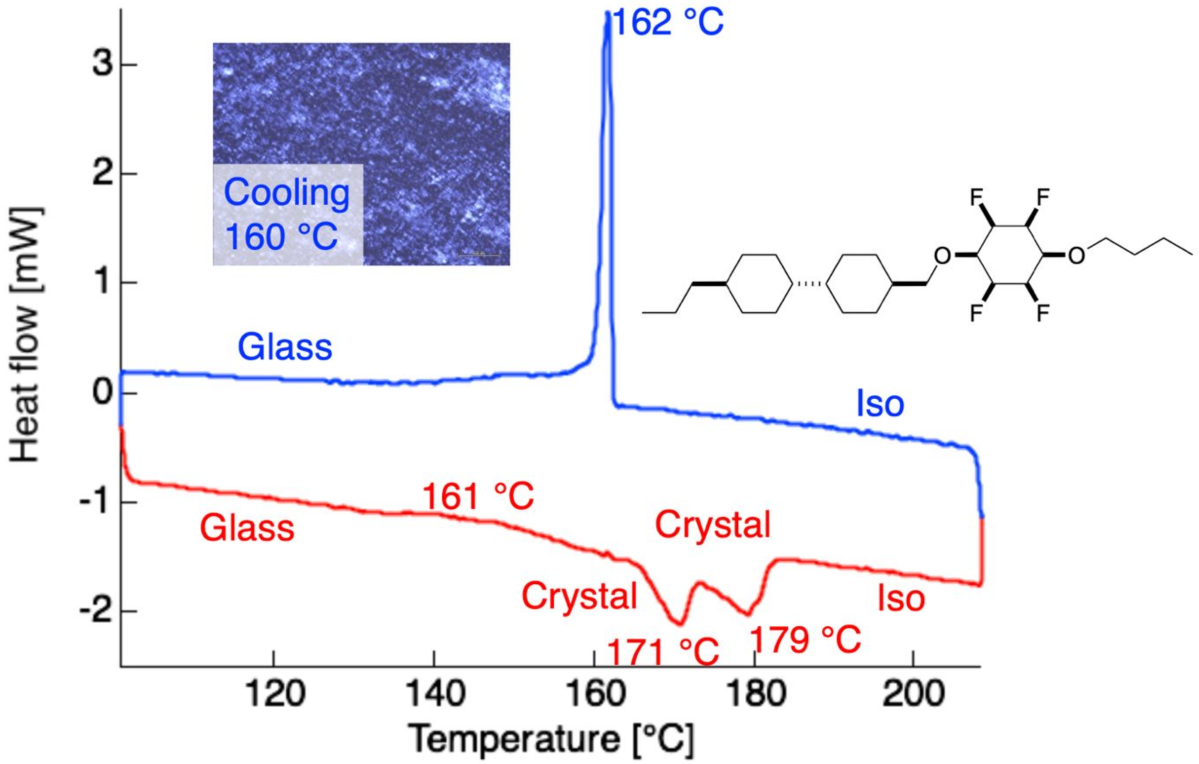

Figure S2 DSC thermogram of **1c** during the second heating (red line) and cooling (blue line) processes under N_2 atmosphere. Inset: POM image at 160 °C on cooling process.

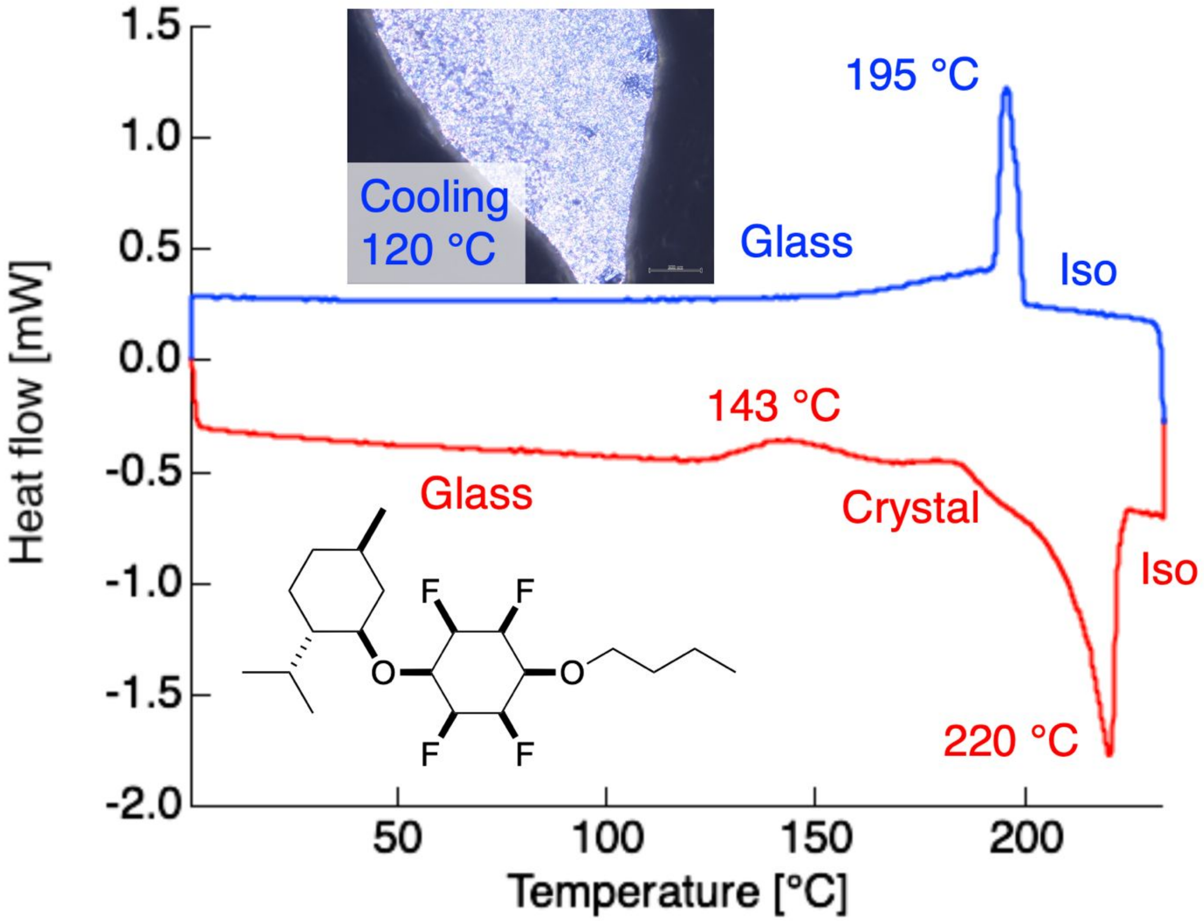

Figure S3 DSC thermogram of **1d** during the second heating (red line) and cooling (blue line) processes under N_2 atmosphere. Inset: POM image at 120 °C on cooling process.

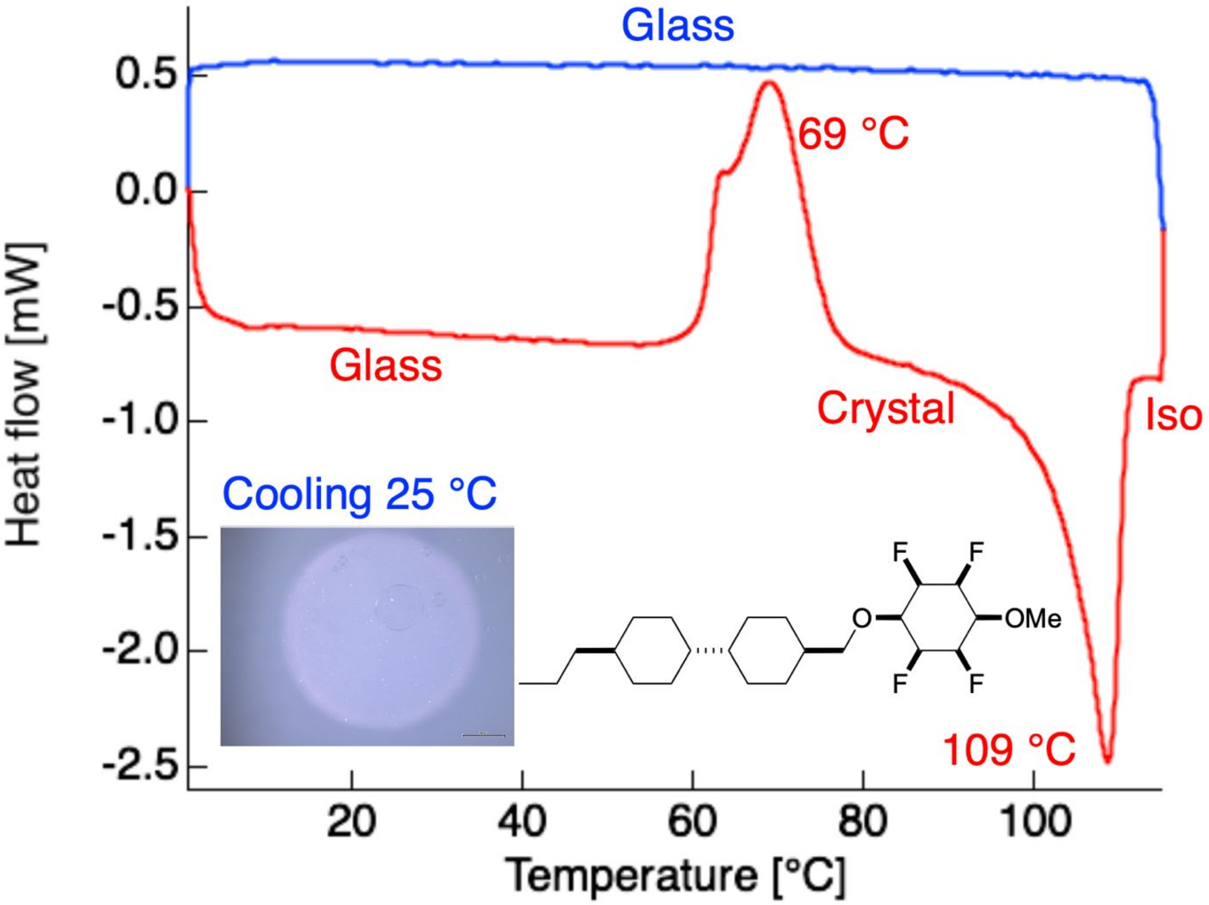

Figure S4 DSC thermogram of **1e** during the second heating (red line) and cooling (blue line) processes under N_2 atmosphere. Inset: POM image at 25 °C on cooling process.

6. Details of X-ray Crystallography

Compounds **1a** and **1d** were both crystallised using a mixed solvent diffusion method, with the compound dissolved in dichloromethane in each case and placed inside a vial and the vial placed in a larger sample bottle containing diethyl ether. Diethyl ether diffused into the dichloromethane solution and crystals formed in the inner vial. X-ray diffraction data for suitable crystals of **1a** and **1d** were collected at 100 K using a Rigaku MM-007HF High Brilliance RA generator/confocal optics with XtaLAB P200 diffractometer [Cu Kα radiation (λ = 1.54187 Å)]. Data for all compounds analysed were collected (using a calculated strategy) and processed (including correction for Lorentz, polarization and absorption) using CrysAlisPro.[1] Structures were solved by dual-space methods (SHELXT[2]) and refined by full-matrix least-squares against F^2 (SHELXL-2019/3[3]). Non-hydrogen atoms were refined anisotropically, and hydrogen atoms were refined using a riding model. All calculations were performed using the Olex2[4] interface. Selected crystallographic data are presented in Table S1. CCDC 2384410-2384411 contains the supplementary crystallographic data for this paper. These data can be obtained free of charge from The Cambridge Crystallographic Data Centre via www.ccdc.cam.ac.uk/structures.

Table S1 Selected crystallographic data.

	**1a**	**1d**
formula	$C_{17}H_{28}F_4O_2$	$C_{20}H_{34}F_4O_2$
fw	340.39	382.47
temperature [K]	100	100
crystal description	Colourless plate	Colourless needle
crystal size [mm^3]	0.47 × 0.1 × 0.01	0.1 × 0.02 × 0.01
space group	*Pbca*	$P2_12_12_1$
a [Å]	9.8423(2)	9.6282(7)
b [Å]	12.5202(5)	18.414(2)
c [Å]	28.6632(11)	23.668(2)
vol [Å]^3	3532.1(2)	4196.2(7)
Z	8	8
ρ (calc) [g/cm^3]	1.280	1.211
μ [mm^{-1}]	0.939	0.843
F(000)	1456	1648
reflections collected	66007	22565
independent reflections (R_{int})	3711 (0.1439)	3482 (0.2129)
parameters, restraints	267, 127	498, 46
GooF on F^2	1.054	1.024
R_1 [$I > 2\sigma(I)$]s	0.1499	0.0773
wR_2 (all data)	0.4188	0.1935
largest diff. peak/hole [e/Å^3]	0.44/-0.41	0.34/-0.23
flack parameter		0.1(3)

X-Ray structure of 1a (minor component of disorder omitted)

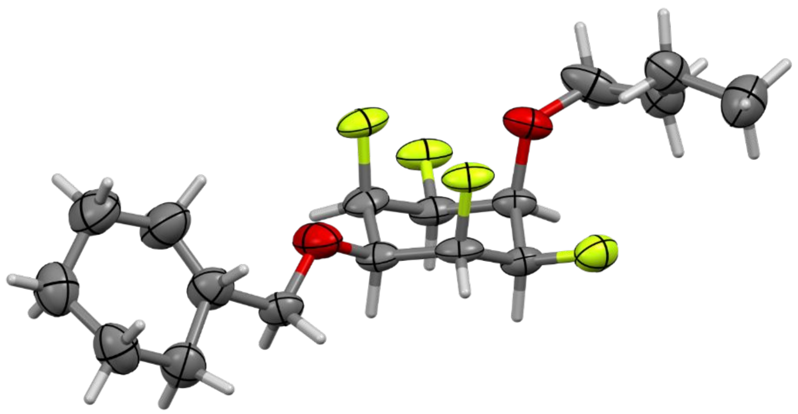

Figure S5 Thermal ellipsoid plot of the structure of **1a**. Ellipsoids are drawn at the 30 % probability level, and the minor component of disorder has been omitted.

X-Ray structure 1d (minor component of disorder omitted)

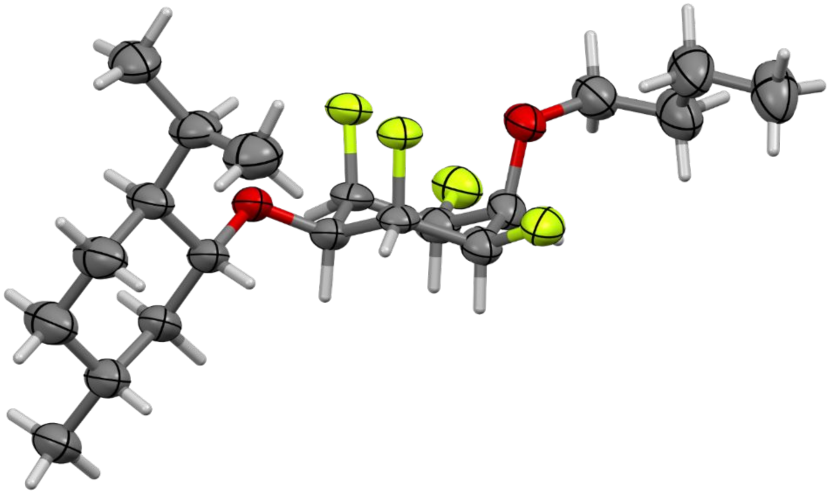

Figure S6 Thermal ellipsoid plot of the structure of **1d**. Ellipsoids are drawn at the 30 % probability level, and the minor component of disorder has been omitted.

References

1. *CrysAlisPro* v1.171.42.94a & 96a Rigaku Oxford Diffraction, Rigaku Corporation, *Tokyo,* Japan, 2023.
2. Sheldrick, G. M. SHELXT – Integrated space-group and crystal structure determination. *Acta Crystallogr., Sect. A: Found. Adv*. **2015**, *71*, 3-8. doi: 10.1107/S2053273314026370

3. Sheldrick, G. M. Crystal structure refinement with SHELXL. *Acta Crystallogr., Sect. C: Struct. Chem.* **2015**, *71*, 3-8. Doi: 10.1107/S2053229614024218
4. Dolomanov, O. V.; Bourhis, L. J.; Gildea, R. J.; Howard, J. A. K.; Puschmann, H. OLEX2: a complete structure solution, refinement and analysis program. *J. Appl. Crystallogr*. **2009**, *42*, 339-341. doi: 10.1107/S0021889808042726

7. Reaction barrier calculations S_NAr

Reaction and transition state search

Computational details: Transition states for *meta* and *para* nucleophile approaches were found by a restricted conformational search in the gas phase at the GFN2-xTB level using CREST. All conformers were reoptimized and had their frequencies calculated at standard temperature and pressure in Gaussian 16 at the M06-2X/6-311++G** level. Solvent effects were included using the IEFPCM method with parameters of tetrahydrofuran. IRC calculations were performed to characterize the found transition states. Resulting reactants and products obtained from the IRC calculations were reoptimized to calculate relative Gibbs free energy barriers. The above workflow was run in a development version of Autobench.

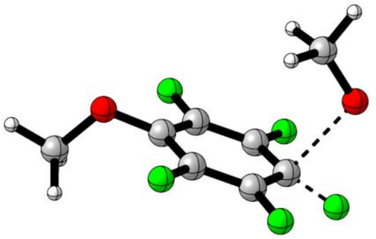

9-methoxide-TS-*para-trans-endo*
1.27

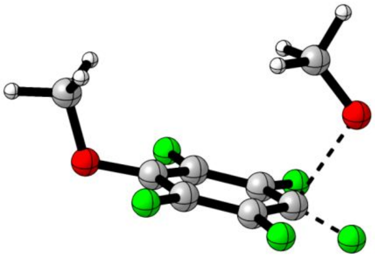

9-methoxide-TS-*para-cis-endo*
1.23

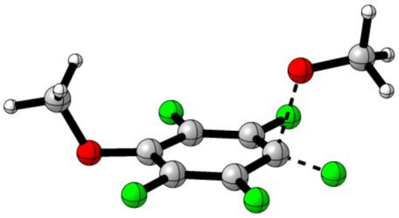

9-methoxide-TS-*para-cis-exo*
2.38

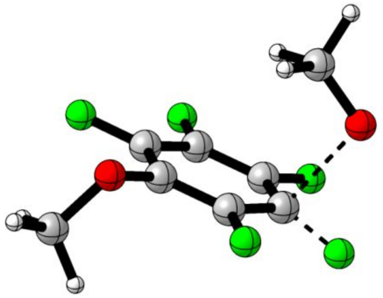

9-methoxide-TS-*meta-trans-endo*
1.59

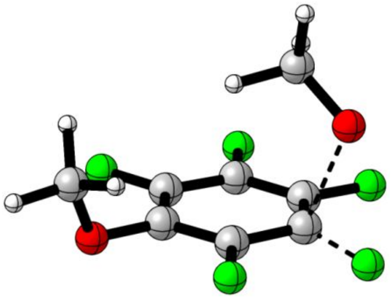

9-methoxide-TS-*meta-cis-endo*
2.11

Figure S7 Transition states found at the M06-2X/6-31++G** level for **9** as substrate and methoxide as nucleophile for the *para* and *meta*-approaches. Relative Gibbs free energies in kcal mol^{-1} to starting materials are given for each case.

Table S2 Transition state cartesian coordinates, electronic energy, and lowest harmonic vibrational frequency (LHVF) for **9** as substrate and methoxide as nucleophile for the *para* and *meta* at the *endo* approaches.

**9**-*methoxyde-para*-TS-*trans*				**9**-*methoxyde-meta*-TS- *trans*			
Electronic Energy (Hartree) = -958.065172				Electronic Energy (Hartree) = -958.064169			
LHVF (cm^{-1}) = -214.0186				LHVF (cm^{-1}) = -225.3624			
C	-1.291792	0.105291	-0.667234	C	1.084186	-0.549204	-0.631237
C	-0.664954	-1.129549	-0.531474	C	1.200741	0.836140	-0.549145
C	0.639656	-1.220144	-0.081663	C	0.147563	1.617699	-0.109553
C	1.397695	-0.088391	0.196018	C	-1.070330	1.039517	0.196743
C	0.773843	1.144373	0.021301	C	-1.237655	-0.338397	0.084866
C	-0.526134	1.244929	-0.431432	C	-0.169807	-1.100119	-0.367505
F	1.462399	2.264750	0.276127	F	0.307145	2.941683	-0.001837
F	1.187741	-2.431848	0.070482	F	2.361545	1.420942	-0.867969
F	-1.356759	-2.247129	-0.788090	F	1.965125	-1.231485	-1.368687
F	-2.407687	0.203682	-1.389362	F	-0.322946	-2.424902	-0.500573
F	-1.077399	2.450598	-0.609238	O	2.269285	-1.233670	1.104108
O	-2.664435	0.157025	1.109815	C	1.536512	-0.864074	2.200459
C	-1.875462	-0.221654	2.162357	H	2.117907	-0.878630	3.144463
H	-2.320614	0.001524	3.153569	H	1.130173	0.171118	2.129486
H	-1.644088	-1.311249	2.186982	H	0.650915	-1.510992	2.391651
H	-0.882458	0.284746	2.173679	O	-2.415200	-0.931659	0.423111
O	2.671648	-0.184559	0.669641	C	-3.435868	-0.779187	-0.568908
C	3.669061	-0.005718	-0.341286	H	-3.119735	-1.240522	-1.507572
H	4.632462	-0.103986	0.154330	H	-4.316869	-1.287884	-0.184337
H	3.567476	-0.772706	-1.112909	H	-3.659507	0.277754	-0.729473
H	3.583378	0.986657	-0.790083	F	-2.085048	1.806823	0.628368
**9**-*methoxyde-para*-TS-*cis*				**9**-*methoxyde-meta*-TS-*cis*			
Electronic Energy (Hartree) = -958.066088				Electronic Energy (Hartree) = -958.065496			
LHVF (cm^{-1}) = -221.6382				LHVF (cm^{-1}) = -226.3876			
C	-1.291792	0.105291	-0.667234	C	0.967111	-0.766542	-0.602166
C	-0.664954	-1.129549	-0.531474	C	1.447275	0.525889	-0.389270
C	0.639656	-1.220144	-0.081663	C	0.585772	1.570616	-0.120121
C	1.397695	-0.088391	0.196018	C	-0.785787	1.374723	-0.138859
C	0.773843	1.144373	0.021301	C	-1.302896	0.115417	-0.418279
C	-0.526134	1.244929	-0.431432	C	-0.415261	-0.921970	-0.677753
F	1.462399	2.264750	0.276127	F	1.082140	2.784469	0.147109
F	1.187741	-2.431848	0.070482	F	2.768096	0.742821	-0.374223
F	-1.356759	-2.247129	-0.788090	F	1.757987	-1.673585	-1.183647
F	-2.407687	0.203682	-1.389362	F	-0.907888	-2.135455	-0.974792
F	-1.077399	2.450598	-0.609238	O	1.506086	-1.744883	1.279041
O	-2.664435	0.157025	1.109815	C	0.884994	-1.021486	2.262648
C	-1.875462	-0.221654	2.162357	H	1.339685	-0.021153	2.441664
H	-2.320614	0.001524	3.153569	H	-0.190653	-0.821120	2.050589
H	-1.644088	-1.311249	2.186982	H	0.892829	-1.525651	3.249448
H	-0.882458	0.284746	2.173679	O	-2.649093	-0.075979	-0.482070
O	2.671648	-0.184559	0.669641	C	-3.177467	-0.768409	0.655734
C	3.669061	-0.005718	-0.341286	H	-3.002357	-0.185717	1.563255
H	4.632462	-0.103986	0.154330	H	-4.245864	-0.873156	0.480834
H	3.567476	-0.772706	-1.112909	H	-2.716882	-1.753539	0.752292
H	3.583378	0.986657	-0.790083	F	-1.606462	2.403728	0.124947

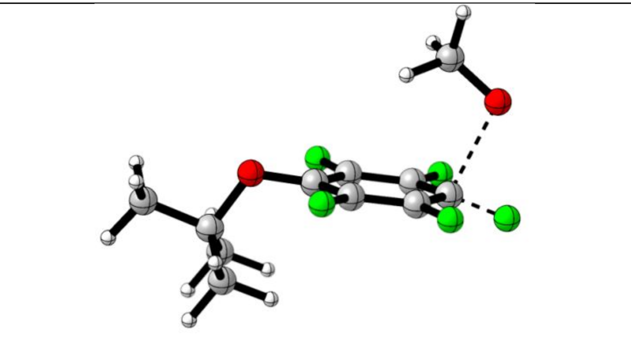	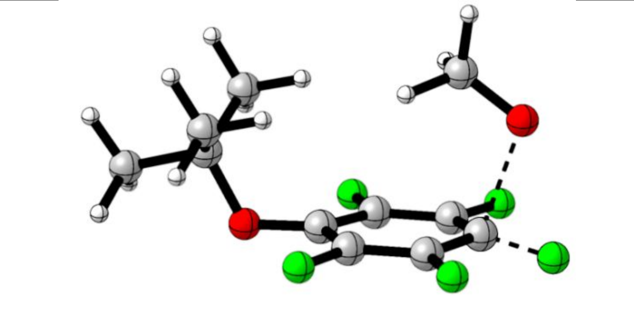
**11**-*methoxide*-*para*-TS--*trans*	**11**-*methoxide*-*para*-TS-*cis*
**1.10**	**1.54**
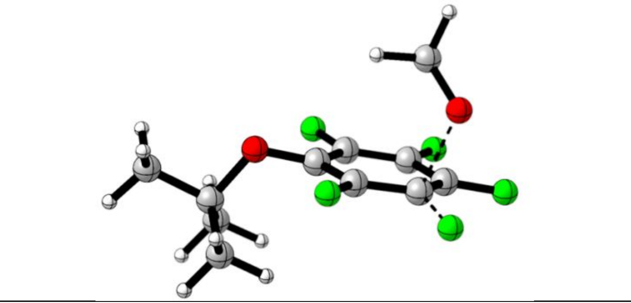	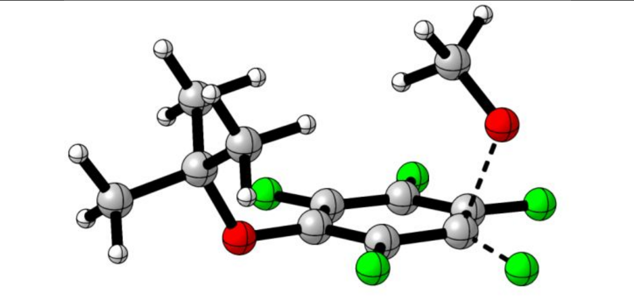
**11**-*methoxide*-*meta*-TS-*trans*	**11**-*methoxide*-*meta*-TS-*cis*
**1.96**	**2.89**

Figure S8 Transition states found at the M06-2X/6-31++G** level for **11** as substrate and methoxide as nucleophile for the *para* and *meta* at the *endo* approaches. Relative Gibbs free energies in kcal mol^{-1} to starting materials are given for each case.

Table S3 Cartesian coordinates, electronic energy, and lowest harmonic vibrational frequency (LHVF) for **11** as substrate and methoxide as nucleophile for *para* and *meta* at the *endo* approaches transition states.

**11**-*methoxyde-para*-TS--*trans*				**11**-*methoxyde-meta*-TS-*trans*			
Electronic Energy (Hartree) = -1075.994326<br>LHVF (cm^{-1}) = -236.9572				Electronic Energy (Hartree) = -1075.990874<br>LHVF (cm^{-1}) = -219.3051			
C	-1.992146	0.072226	-0.674067	C	1.561963	-0.758197	-0.604729
C	-1.316818	-1.131963	-0.490615	C	2.011737	0.559522	-0.625491
C	-0.039248	-1.163029	0.032711	C	1.226219	1.585533	-0.136414
C	0.671838	0.001050	0.324546	C	-0.058698	1.334131	0.311554
C	-0.008736	1.199860	0.125331	C	-0.572765	0.038651	0.288171
C	-1.291934	1.240790	-0.388643	C	0.248809	-0.977345	-0.189144
F	0.591732	2.359269	0.422962	F	1.708999	2.834142	-0.112987
F	0.526756	-2.356488	0.254311	F	3.241257	0.830135	-1.080402
F	-1.941933	-2.281808	-0.774363	F	2.168842	-1.664305	-1.379183
F	-3.051219	0.124054	-1.483923	F	-0.203086	-2.240307	-0.191654
F	-1.881415	2.425723	-0.596279	O	2.756774	-1.612437	1.028110
O	-3.498445	-0.028673	0.970730	C	2.231864	-1.064167	2.168818
C	-2.768022	-0.256527	2.105787	H	2.867416	-1.219296	3.063636
H	-2.515696	-1.327388	2.276441	H	2.091986	0.039172	2.102194
H	-1.788716	0.276160	2.111411	H	1.232272	-1.468474	2.445535
H	-3.276203	0.075159	3.034391	O	-1.807508	-0.231127	0.790889
O	1.916045	-0.033602	0.876083	C	-2.953126	-0.184227	-0.122804
C	3.064916	-0.024211	-0.034328	F	-0.789367	2.349495	0.799821
C	4.257438	-0.035316	0.908703	C	-3.086952	1.206437	-0.731974
H	5.186313	-0.033574	0.335116	H	-3.974827	1.235598	-1.367130
H	4.239051	0.847394	1.550684	H	-3.187244	1.960986	0.049131
H	4.233762	-0.928023	1.536535	H	-2.221633	1.452423	-1.352554
C	3.048003	-1.271173	-0.910221	C	-4.132096	-0.496902	0.784914
H	3.041004	-2.172197	-0.295457	H	-5.058042	-0.494694	0.206663
H	2.172499	-1.283237	-1.564222	H	-4.005109	-1.479713	1.242610
H	3.938972	-1.281044	-1.541677	H	-4.208815	0.252453	1.574918
C	3.052028	1.240256	-0.884898	C	-2.802393	-1.243475	-1.207838
H	3.049731	2.129151	-0.252804	H	-2.692404	-2.233842	-0.764717
H	3.942690	1.258540	-1.516533	H	-3.690786	-1.236044	-1.843024
H	2.176587	1.268735	-1.538602	H	-1.934111	-1.042273	-1.839991

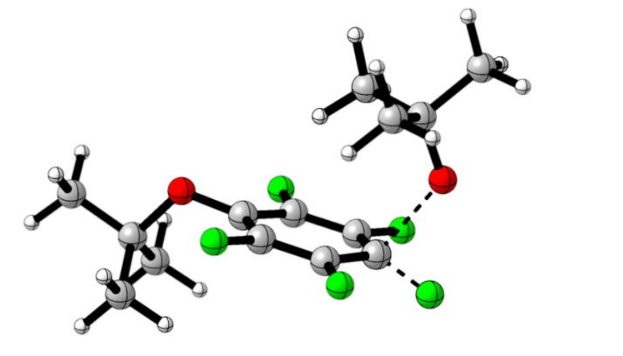	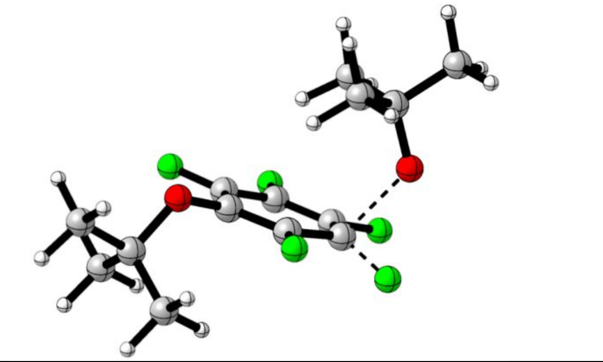
**11**-*tert*-butoxide-*para*-TS-*trans*	**11**-*tert*-butoxide-*meta*-TS-*cis*
**4.26**	**6.77**

Figure S9 Transition states found at the M06-2X/6-31++G** level for **11** as substrate and *tert*-butoxide as nucleophile for the *para* and *meta* at the *endo* approaches. Relative Gibbs free energies in kcal mol^{-1} to starting materials are given for each case.

Table S4 Cartesian coordinates, electronic energy, and lowest harmonic vibrational frequency (LHVF) for **11** *tert*-butoxide transition states.

**11-*tert*-butoxide-*para*-TS-*trans***				**11**-*tert*-butoxide-*meta*-TS-*cis*			
Electronic Energy (Hartree) = -1193.914695<br>LHVF (cm^{-1}) = -236.8633				Electronic Energy (Hartree) = -1193.913125<br>LHVF (cm^{-1}) = -235.0393			
C	-1.236385	0.302459	-1.317836	C	-1.245128	0.499881	1.195278
C	-0.625510	-0.945110	-1.157842	C	-1.472381	1.627709	0.394220
C	0.513746	-1.097184	-0.395490	C	-0.470417	2.179089	-0.370473
C	1.180424	-0.011489	0.173346	C	0.825119	1.687978	-0.307137
C	0.604257	1.238894	-0.048385	C	1.126195	0.618503	0.531480
C	-0.538204	1.399671	-0.803941	C	0.085499	0.064841	1.275057
F	1.155508	2.327345	0.507718	F	-0.744603	3.221068	-1.168838
F	0.983040	-2.337666	-0.197219	F	-2.703382	2.153520	0.339634
F	-1.204870	-2.026413	-1.702907	F	-2.016760	0.333277	2.288794
F	-2.031077	0.496702	-2.380758	F	0.362033	-0.929049	2.141018
F	-1.038496	2.631462	-0.990739	O	-2.545591	-0.866627	0.331525
O	-3.059384	0.225684	-0.251094	C	-2.185031	-1.658736	-0.738118
C	-3.141844	-0.201621	1.058619	O	2.417619	0.239586	0.744241
O	2.258919	-0.179466	0.990312	C	3.023374	-0.770686	-0.123965
C	3.591792	-0.130041	0.386382	F	1.792919	2.314918	-1.000135
C	3.847417	1.252403	-0.201610	C	3.091300	-0.264473	-1.559904
H	3.731351	2.021339	0.563441	H	3.653790	0.668433	-1.615084
H	4.865107	1.296038	-0.595600	H	3.586422	-1.014252	-2.181050
H	3.158345	1.465438	-1.022553	H	2.090258	-0.097791	-1.966079
C	4.516901	-0.403078	1.561961	C	2.232946	-2.068907	-0.047687
H	4.377740	0.354755	2.335325	H	1.239204	-1.944805	-0.480876
H	4.303467	-1.384853	1.988892	H	2.752813	-2.843731	-0.615757
H	5.557359	-0.381861	1.232098	H	2.128374	-2.399187	0.987064
C	3.729902	-1.211069	-0.678852	C	-3.456529	-2.133120	-1.469221
H	4.745093	-1.193374	-1.081025	H	-3.224619	-2.797015	-2.310137
H	3.536584	-2.196774	-0.253603	H	-4.102389	-2.669180	-0.767854
H	3.037146	-1.040961	-1.506840	H	-4.007293	-1.267359	-1.848048
C	-4.468761	0.296932	1.666491	C	-1.418243	-2.904673	-0.247257
H	-4.605743	-0.043055	2.699493	H	-0.586607	-2.592579	0.388497
H	-4.487322	1.390718	1.653112	H	-2.085073	-3.523065	0.361143
H	-5.306362	-0.065628	1.064373	H	-1.028109	-3.517125	-1.068693
C	-1.993330	0.340904	1.938925	C	-1.315796	-0.899917	-1.768041
H	-2.157883	0.104749	2.995709	H	-1.847418	-0.005439	-2.107521
H	-1.036292	-0.096294	1.644738	H	-0.368774	-0.580542	-1.328335
H	-1.924043	1.428326	1.832485	H	-1.093496	-1.523764	-2.640447
C	-3.118886	-1.742884	1.144075	C	4.416239	-0.939553	0.462803
H	-2.180628	-2.125412	0.731461	H	4.350326	-1.282470	1.497172
H	-3.214989	-2.104788	2.173988	H	4.977480	-1.675286	-0.116429
H	-3.941264	-2.153696	0.550852	H	4.954140	0.010261	0.439839

8. Details of Theory/Computation for cyclohexane interconversions 1f, 2 and 15

The accessible conformers for the structures [chair → half-chair (TS) → twisted boat → boat (TS)] associated with the ring flip of compounds **1f**, **2** and **15** were explored using the iterative workflow approach with static metadynamics simulations (--v4 keyword) as implemented in CREST software[1] at the semiempirical GFN2-xTB level of theory.[2] For the conformational search of transition state structures, the dihedral angles consisting of carbon atoms of the ring were kept frozen to avoid the TSs optimizing to a minimum structure during CREST routine. In each case, the most stable conformation from the conformational search was used as starting structure to a full geometry optimization at DFT level, employing the hybrid Minnesota family functional M06-2X[3] and Pople's triple ζ with polarization and diffuse functions 6-311++G(d,p) basis set[4] in Gaussian 16 rev C.01 program.[5] Thermal corrections for each stationary point was obtained from frequency calculations at standard temperature and pressure within the harmonic oscillator and rigid rotor models at the M06-2X/def2-TZVP theoretical level. No imaginary frequency was found for minima geometries, and a single imaginary frequency, corresponding to the ring flip, was found for the saddle points (TSs).

1. (a) Pracht, P.; F. Bohle, F.; Grimme, S. Automated Exploration of the Low-Energy Chemical Space with Fast Quantum Chemical Methods. *Phys. Chem. Chem. Phys*. **2020**, *22*, 7169-7192. (b) Grimme, S. Exploration of Chemical Compound, Conformer, and Reaction Space with Meta-Dynamics Simulations Based on Tight-Binding Quantum Chemical Calculations. *J. Chem. Theory. Comput.,* **2019**, *15*, 2847-2862.

2. Bannwarth C.; Ehlert, S.; Grimme, S. GFN2-xTB-An Accurate and Broadly Parametrized Self-Consistent Tight-Binding Quantum Chemical Method with Multipole Electrostatics and Density-Dependent Dispersion Contributions. *J. Chem. Theory Comput.,* **2019**, *15*, 1652–1671.

3. Zhao, Y.; D. Truhlar, D. G., The Mo6 Suite of Density Functionals for Main Group Thermochemistry, Thermochemical Kinetics, Noncovalent Interactions, Excited States, and Transition Elements: Two New Functionals and Systematic Testing of Four Mo6-class Functionals and 12 Other Functionals. *Theor. Chem. Acc.,* **2008**, *120*, 215–241.

4. (a) Krishnan, R., Binkley, J. S., Pople, J. A., Self-consistent molecular orbital methods. XX. A basis set for correlated wave functions. *J. Chem. Phys.*, **1980**, *72*, 650-654; (b) McLean, A. D., Chandler, G. S., Contracted Gaussian basis sets for molecular calculations. I. Second row atoms, *Z*=11–18. *J. Chem. Phys.*, **1980**, *72*, 5639-5648; (c) Clark, T., Chandrasekhar, J., Spitznagel, G. W., Schleyer, P. R., Efficient diffuse function-augmented basis sets for anion calculations. III.[†] The 3-21+G basis set for first-row elements, Li–F. **1983**, *4*, 294-301.

5. Gaussian 16 Revision C.01; Frisch, M. J.; Trucks, G. W.; Schlegel, H. B.; Scuseria, G. E.; Robb, M. A.; Cheeseman, J. R.; Scalmani, G.; Barone, V.; Petersson, G. A.; Nakatsuji, H.; Li, X.; Caricato, M.; Marenich, A. V; Bloino, J.; Janesko, B. G.; Gomperts, R.; Mennucci, B.; Hratchian, H. P.; Ortiz, J. V; Izmaylov, A. F.; Sonnenberg, J. L.; Williams-Young, D.; Ding, F.; Lipparini, F.; Egidi, F.; Goings, J.; Peng, B.; Petrone, A.; Henderson, T.; Ranasinghe, D.; Zakrzewski, V. G.; Gao, J.; Rega, N.; Zheng, G.; Liang, W.; Hada, M.; Ehara, M.; Toyota, K.; Fukuda, R.; Hasegawa, J.; Ishida, M.; Nakajima, T.; Honda, Y.; Kitao, O.; Nakai, H.; Vreven, T.; Throssell, K.; Montgomery Jr., J. A.; Peralta, J. E.; Ogliaro, F.; Bearpark, M. J.; Heyd, J. J.; Brothers, E. N.; Kudin, K. N.; Staroverov, V. N.; Keith, T. A.; Kobayashi, R.; Normand, J.; Raghavachari, K.; Rendell, A. P.;

Burant, J. C.; Iyengar, S. S.; Tomasi, J.; Cossi, M.; Millam, J. M.; Klene, M.; Adamo, C.; Cammi, R.; Ochterski, J. W.; Martin, R. L.; Morokuma, K.; Farkas, O.; Foresman, J. B.; Fox, D. J.; Inc., G. Gaussian 16 Revision C.01. *Wallingford CT*. Gaussian Inc., Walingford CT 2016.

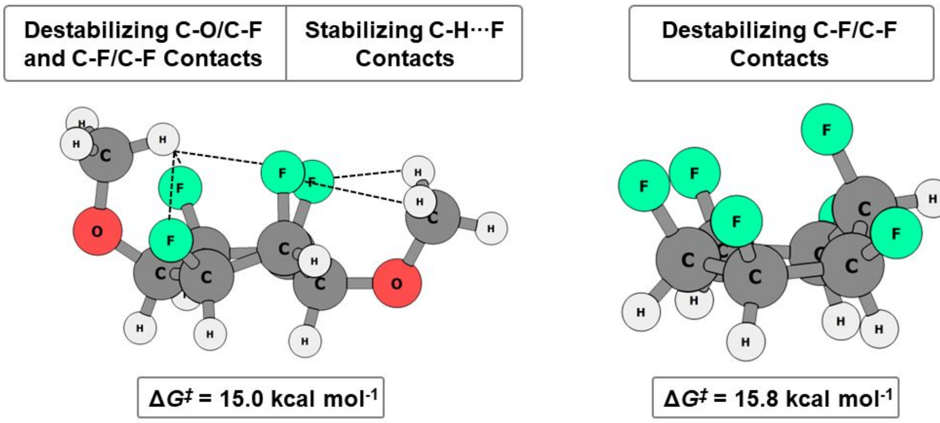

Figure S10 CH···F stabilizing contacts in 'half-chair' TS of **1f** contributes to lowering its energy compared to the same TS of **2**.

Table S5 Cartesian coordinates, electronic energy, and lowest harmonic vibrational frequency (LHVF) for the structures associated with the ring flip of compounds **1f**, **2** and **15**.

**1f (chair)<br>Electronic Energy (Hartree) = -861.783004<br>LHVF (cm^{-1}) = 49.99**				**1f (half-chair TS 1)<br>Electronic Energy (Hartree) = -861.761248<br>LHVF (cm^{-1}) = 73.41i**			
C	-1.43415800	-0.15742000	-0.80042300	C	-1.47124700	0.03988200	-0.95299000
C	-0.46012200	-1.29967100	-0.49475800	C	-0.72664500	-1.31947000	-0.71829400
C	0.91362700	-0.94570900	-1.04069300	C	0.63614700	-1.21250100	-0.06790900
C	1.47168200	0.36845900	-0.47955400	C	1.44269700	-0.16036600	-0.81742800
C	0.46422400	1.46353900	-0.83430200	C	0.90414000	1.24022700	-0.53559300
C	-0.93528900	1.19710700	-0.28655400	C	-0.63497300	1.35775100	-0.74261500
H	-1.61374400	1.98636200	-0.62529700	H	-0.57892100	-1.79843600	-1.69095900
H	-1.43192600	-0.06933200	-1.89519400	H	1.13781400	-2.18355500	-0.08952100
H	-0.82658800	-2.21506800	-0.96766700	H	1.26996300	-0.34219400	-1.88762700
H	0.83030300	-0.85100400	-2.13035600	H	1.42157200	1.94496300	-1.19011600
H	2.42105000	0.58090800	-0.99599100	H	-0.81135000	1.98000000	-1.62245200
H	0.38841300	1.53150400	-1.92634500	H	-1.73420100	0.04170600	-2.01320700
F	-0.91939700	1.24007900	1.08894700	F	1.21871400	1.61232400	0.75262600
F	0.91308500	2.67873200	-0.37906200	F	-1.14640900	2.05664100	0.32240100
F	1.79276300	-1.98281400	-0.79523400	F	-1.51102700	-2.17082200	0.02419600
F	-0.37247500	-1.55541700	0.85927900	F	0.47713400	-0.86031300	1.26169500
O	1.65512900	0.38974000	0.89879900	O	2.83283200	-0.24595600	-0.66356600
O	-2.76337900	-0.46300600	-0.48880600	O	-2.70059400	0.10516500	-0.31010400
C	2.71203700	-0.40865800	1.39961000	C	3.33250300	-0.44259200	0.65647700
H	2.41702800	-1.45679100	1.47488300	H	4.41578500	-0.46703700	0.55573300
H	3.60015400	-0.32749600	0.76202500	H	3.03765700	0.36456800	1.32644500
H	2.94364000	-0.02089400	2.39020600	H	2.99373900	-1.39660900	1.07061900
C	-3.17762400	-0.37244900	0.87472300	C	-2.71213700	0.03114800	1.11867600
H	-3.29375200	0.66954300	1.17990800	H	-3.32520800	-0.82383400	1.40400200
H	-4.14483700	-0.87032800	0.91489300	H	-1.71084000	-0.09263800	1.53077600
H	-2.47578300	-0.86769400	1.54533100	H	-3.14253300	0.95444400	1.50546100
**1f (half-chair TS 2)<br>Electronic Energy (Hartree) = -861.762261<br>LHVF (cm^{-1}) = 78.65i**				**1f (half-chair TS 3)<br>Electronic Energy (Hartree) = -861.75923<br>LHVF (cm^{-1}) = 88.58i**			
C	1.47065700	0.49357800	-0.91272800	C	0.91754700	-1.08159600	-0.86733900
C	0.41367300	1.60420900	-0.60690100	C	1.50861400	0.34540000	-0.77756600
C	-0.97327000	1.09328400	-0.21552700	C	0.62893300	1.26403900	0.05581500
C	-1.28515100	-0.06470000	-1.15936900	C	-0.69148700	1.39339600	-0.67097900
C	-0.47842800	-1.30678500	-0.83857100	C	-1.51620400	0.09658000	-0.62068700
C	1.05782000	-1.02378700	-0.82444400	C	-0.63689000	-1.21549900	-0.70037900
H	0.29831700	2.20583400	-1.51370700	H	1.48659000	0.75113900	-1.79487100
H	-1.71136500	1.89052300	-0.38686700	H	1.09120000	2.24652100	0.18490100
H	-1.04490700	0.24550400	-2.18629200	H	-0.47653800	1.64091200	-1.71562500
H	-0.70058900	-2.08164900	-1.57549600	H	-2.18621000	0.10042800	-1.49183800
H	1.44740100	-1.47313300	-1.74049100	H	-1.00809400	-1.82749700	-1.52530100
H	1.80205300	0.66117100	-1.94092600	H	1.20494100	-1.50079200	-1.83422500
F	-2.63430400	-0.36838200	-1.13659400	F	-1.43023100	2.43486000	-0.16494300
F	-0.88620900	-1.81631800	0.37572700	F	-0.86452700	-1.93678300	0.44303300
F	2.57362500	0.69364100	-0.12442100	F	1.51214300	-1.87895000	0.08037700
F	0.88338900	2.43926400	0.36855600	F	0.41696100	0.71668000	1.30737900
O	-0.93867500	0.75120300	1.13427600	O	-2.27566500	0.12320300	0.54306000
O	1.71303600	-1.71673900	0.18360000	O	2.86628800	0.32918200	-0.44328600
C	-2.20274700	0.48103200	1.71716900	C	-3.44816200	-0.66218100	0.48450100
H	-2.61622900	-0.46130300	1.35409700	H	-3.21755400	-1.72522200	0.37283500
H	-2.03797600	0.41966100	2.79125200	H	-3.97129900	-0.50932400	1.42638200
H	-2.90295700	1.29668700	1.50377500	H	-4.09091500	-0.33335700	-0.34159100
C	1.54710300	-1.27569600	1.53408200	C	3.20327700	0.29809600	0.94106100
H	0.95268800	-2.01196600	2.07598900	H	2.92886600	1.23609600	1.43231000

H	2.54316800	-1.19868900	1.96966200
H	1.04904800	-0.30901000	1.59432700

H	2.72596200	-0.53388400	1.45705000
H	4.28518900	0.18422600	0.97261800

1f (twisted boat 1)
Electronic Energy (Hartree) = -861.772279
LHVF (cm^{-1}) = 60.04

C	-1.37203500	0.12010200	-0.89077500
C	-0.80627700	-1.27143300	-0.51432600
C	0.68410400	-1.24266600	-0.12591100
C	1.37202300	-0.12015500	-0.89078900
C	0.80627100	1.27140100	-0.51441000
C	-0.68410400	1.24265700	-0.12597100
H	-1.10335700	0.27722000	-1.94333400
H	-0.93844600	-1.92904200	-1.37823200
H	1.15268400	-2.20484100	-0.34834800
H	1.10333400	-0.27733400	-1.94333700
H	0.93842900	1.92896200	-1.37835400
H	-1.15268600	2.20481900	-0.34845600
F	-1.51949300	-1.82710000	0.52050900
F	0.80244700	-1.03949300	1.23218000
F	1.51950500	1.82712200	0.52038400
F	-0.80242400	1.03956100	1.23213400
O	-2.76969000	0.14139300	-0.88260200
O	2.76967800	-0.14144300	-0.88262600
C	3.41775700	-0.40723100	0.35930300
H	3.25325600	-1.44153300	0.67382200
H	4.47879300	-0.25743800	0.16922900
H	3.08057500	0.26765000	1.14515700
C	-3.41776500	0.40726600	0.35931100
H	-3.08058100	-0.26756000	1.14521200
H	-3.25326500	1.44159000	0.67375800
H	-4.47880100	0.25745900	0.16925100

1f (twisted boat 2)
Electronic Energy (Hartree) = -861.770077
LHVF (cm^{-1}) = 69.02

C	-1.31409100	-0.12120900	-1.11951800
C	-0.58529200	-1.39496500	-0.66526700
C	0.90884400	-1.12042200	-0.32718900
C	1.31409100	0.12119300	-1.11952100
C	0.58529100	1.39495600	-0.66528600
C	-0.90884400	1.12041800	-0.32720400
H	-1.08314200	0.05626400	-2.17660400
H	-0.65101300	-2.13562800	-1.46738900
H	1.52549900	-1.96212400	-0.67635500
H	1.08314100	-0.05629500	-2.17660400
H	0.65101300	2.13560800	-1.46741900
H	-1.52549900	1.96211400	-0.67638200
F	-2.67625600	-0.33096100	-1.05440500
F	-1.22972000	-1.92245600	0.42068200
F	2.67625600	0.33094600	-1.05441100
F	1.22972000	1.92246200	0.42065400
O	1.02457500	-0.98096800	1.05012800
O	-1.02457500	0.98098300	1.05011500
C	2.34952600	-0.87402000	1.54070900
H	2.75779100	0.12347400	1.36880400
H	2.29191800	-1.06616300	2.61060900
H	3.00177300	-1.62033800	1.07140200
C	-2.34952600	0.87404000	1.54069700
H	-3.00177300	1.62035100	1.07137900
H	-2.75778900	-0.12345700	1.36880700
H	-2.29191700	1.06620000	2.61059500

1f (twisted boat 3)
Electronic Energy (Hartree) = -861.771006
LHVF (cm^{-1}) = 83.28

C	-1.14953500	0.73066600	-1.04667400
C	-1.32380400	-0.78101200	-0.70667500
C	0.02465500	-1.41768300	-0.30123900
C	1.14978600	-0.73106900	-1.04643900
C	1.32414900	0.78072600	-0.70702500
C	-0.02427600	1.41768800	-0.30199800
H	-0.90793900	0.80697200	-2.11410300
H	-1.59984000	-1.26783400	-1.64732600
H	0.01075800	-2.48441000	-0.53494000
H	0.90802200	-0.80782700	-2.11379700
H	1.60057800	1.26720600	-1.64774000
H	-0.01033500	2.48427200	-0.53634700
F	-2.33655500	1.40338400	-0.86358100
F	0.24182800	-1.31955700	1.05999900
F	2.33680500	-1.40376200	-0.86324200
F	-0.24123300	1.32046000	1.05934000
O	-2.37275700	-1.10623000	0.14921300
O	2.37270700	1.10595100	0.14931000
C	-2.50257100	-0.45069500	1.41156600
H	-2.64157700	0.62257000	1.28413100
H	-3.39940800	-0.88065100	1.85421000
H	-1.64650100	-0.63840400	2.05650400
C	2.50111100	0.45100900	1.41210800
H	2.63960000	-0.62240600	1.28535500
H	3.39779500	0.88070200	1.85531500
H	1.64455900	0.63954600	2.05617700

1f (boat TS 1)
Electronic Energy (Hartree) = -861.763777
LHVF (cm^{-1}) = 72.24i

C	1.35485400	0.08543600	0.89876000
C	0.85800900	-1.23212500	0.29717700
C	-0.70193400	-1.30653500	0.25196400
C	-1.35487800	-0.08562700	0.89870700
C	-0.85801700	1.23206100	0.29741400
C	0.70192700	1.30648000	0.25225800
H	1.01718600	0.08300900	1.94107300
H	1.26116400	-2.05770700	0.88948300
H	-1.05863500	-2.20213100	0.76541000
H	-1.01723900	-0.08341900	1.94103000
H	-1.26118700	2.05751900	0.88988200
H	1.05861500	2.20196800	0.76590200
F	1.33690500	-1.38721300	-0.98407600
F	-1.10188500	-1.41371500	-1.06155900
F	-1.33688000	1.38741700	-0.98381900
F	1.10191200	1.41393800	-1.06123300
O	-2.74627700	-0.19843900	1.01519900
O	2.74624900	0.19822200	1.01531700
C	-3.56891300	0.02744200	-0.13027000
H	-4.54573400	-0.37239300	0.13685700
H	-3.66099400	1.09457700	-0.34126600
H	-3.18762200	-0.48293600	-1.01358600
C	3.56891900	-0.02740600	-0.13017700
H	3.66099500	-1.09449300	-0.34141400
H	3.18766100	0.48317800	-1.01338900
H	4.54573500	0.37235900	0.13707100

1f (boat TS 2)
Electronic Energy (Hartree) = -861.767907
LHVF (cm^{-1}) = 49.44i

C	-0.92699200	-0.95230300	-1.10270600
C	0.37061800	-1.45258600	-0.48570000
C	1.47155100	-0.32959800	-0.47343800
C	0.92706900	0.95258300	-1.10239900
C	-0.37058700	1.45271000	-0.48536100
C	-1.47152000	0.32971800	-0.47345600
H	-0.74123700	-0.76722900	-2.16683300
H	0.71047700	-2.32411400	-1.05155400
H	2.32090900	-0.65810600	-1.08959500
H	0.74139100	0.76778000	-2.16658700
H	-0.71040600	2.32438000	-1.05102100
H	-2.32083700	0.65838200	-1.08958500
F	-1.87991300	-1.94120300	-1.03499300
F	0.13288500	-1.87880600	0.79587500
F	1.87998600	1.94146500	-1.03436500
F	-0.13294600	1.87861100	0.79633800
O	1.90500100	-0.03539200	0.81319600
O	-1.90505400	0.03518500	0.81307400
C	2.85205600	-0.95355900	1.32020200
H	3.13444300	-0.59563300	2.30816100
H	2.42956300	-1.95798100	1.41055400
H	3.74144900	-0.98111400	0.67861200
C	-2.85214300	0.95321900	1.32025500
H	-2.42965500	1.95761700	1.41090200
H	-3.74149200	0.98094400	0.67861100
H	-3.13460000	0.59503400	2.30810000

2 (chair)
Electronic Energy (Hartree) = -831.245368
LHVF (cm^{-1}) = 114.77

C	1.39433900	-0.46246000	-0.22576100
C	1.07072000	0.95229300	-0.69277900
C	-0.29671700	1.43862600	-0.22561600
C	-1.36024200	0.45093200	-0.69262500
C	-1.09775300	-0.97620500	-0.22518700
C	0.28915600	-1.40345700	-0.69256100
H	0.29208900	-1.41959900	-1.78992800
H	2.35365900	-0.78086500	-0.64367900
H	1.08319400	0.96332500	-1.79012000
H	-0.50072600	2.42877400	-0.64312400
H	-1.37581200	0.45594700	-1.78998400
H	-1.85340500	-1.64799000	-0.64239000
F	1.50381300	-0.49850000	1.13712600
F	0.55131800	-2.67594400	-0.25494600
F	-1.18306500	-1.05188700	1.13777300
F	-2.59334600	0.86008700	-0.25498200
F	-0.31982100	1.55090700	1.13731100
F	2.04154300	1.81556300	-0.25490500

2 (half-chair TS)
Electronic Energy (Hartree) = -831.220351
LHVF (cm^{-1}) = 87.82i

C	-1.36265500	0.48552100	-0.71036000
C	-1.14608800	-1.03431200	-0.51586000
C	0.12243100	-1.34873900	0.24452000
C	1.26791500	-0.83146700	-0.59897700
C	1.32266200	0.69748300	-0.59860600
C	-0.08376500	1.39295300	-0.61771400

2 (twisted boat)
Electronic Energy (Hartree) = -831.232340
LHVF (cm^{-1}) = 80.99

C	1.28912000	-0.40096200	-0.83237800
C	1.07455600	1.06739300	-0.41703700
C	-0.40301300	1.36154500	-0.06594200
C	-1.28912000	0.40096100	-0.83237900
C	-1.07455600	-1.06739300	-0.41703700
C	0.40301300	-1.36154500	-0.06594000

H	-1.07993600	-1.51266900	-1.49869400
H	0.21731900	-2.42225600	0.42456900
H	1.12790800	-1.18394400	-1.62757000
H	1.90674400	1.03194000	-1.45897400
H	-0.11032400	2.09016000	-1.45719400
H	-1.82906000	0.64342200	-1.68577500
F	2.47064700	-1.32344800	-0.16110400
F	1.99518300	1.10399300	0.51739400
F	-0.18546100	2.14159800	0.51625700
F	-2.25235300	0.92361800	0.22627500
F	-2.23315800	-1.56886400	0.12101300
F	0.09895800	-0.70081700	1.45634400

H	1.05269200	-0.49805400	-1.89964300
H	1.38741100	1.71122400	-1.24419300
H	-0.65950500	2.39483600	-0.30950500
H	-1.05269100	0.49805200	-1.89964400
H	-1.38741000	-1.71122500	-1.24419100
H	0.65950500	-2.39483600	-0.30950200
F	2.60910800	-0.73063700	-0.67428800
F	1.85606600	1.37053600	0.65874100
F	-0.60607800	1.19092400	1.27615500
F	-2.60910800	0.73063600	-0.67428900
F	-1.85606700	-1.37053500	0.65874100
F	0.60607700	-1.19092300	1.27615600

2 (boat TS)
Electronic Energy (Hartree) = -831.227949
LHVF (cm^{-1}) = 67.72i

C	-1.33421500	-0.00002400	-0.87033800
C	-0.78124900	-1.27612900	-0.24739200
C	0.78124300	-1.27612900	-0.24741300
C	1.33419300	-0.00002300	-0.87037300
C	0.78124300	1.27611600	-0.24748100
C	-0.78125000	1.27611600	-0.24746100
H	-1.08709000	-0.00005200	-1.93861000
H	-1.16618400	-2.13515600	-0.80191600
H	1.16616400	-2.13515600	-0.80194600
H	1.08704000	-0.00005200	-1.93863800
H	1.16616300	2.13511300	-0.80206100
H	-1.16618400	2.13511300	-0.80203100
F	-2.70239500	-0.00002100	-0.76997800
F	-1.23172300	-1.38394300	1.03694700
F	1.23175100	-1.38394300	1.03691500
F	2.70237500	-0.00002000	-0.77004800
F	1.23175000	1.38399800	1.03684100
F	-1.23172400	1.38399800	1.03687300

15 (chair)
Electronic Energy (Hartree) = -235.816855
LHVF (cm^{-1}) = 237.93

C	0.51880300	1.36224400	0.23403300
C	-0.92044400	1.13049800	-0.23377400
C	-1.43929600	-0.23192500	0.23368300
C	-0.51881200	-1.36226500	-0.23403900
C	0.92043100	-1.13048200	0.23376300
C	1.43931800	0.23193000	-0.23368900
H	1.48778300	0.23980500	-1.32967300
H	0.88591300	2.32606000	-0.12899900
H	-0.95128100	1.16859900	-1.32976900
H	-2.45747300	-0.39581400	-0.12958800
H	-0.53622800	-1.40790600	-1.33002200
H	1.57158300	-1.93026000	-0.12934300
H	0.53618600	1.40779900	1.33001300
H	2.45747800	0.39581600	0.12964600
H	0.95121100	-1.16851200	1.32975100
H	-0.88591600	-2.32606500	0.12905900
H	-1.48766900	-0.23978500	1.32966600
H	-1.57158500	1.93026100	0.12939800

15 (half-chair TS)
Electronic Energy (Hartree) = -235.797757
LHVF (cm^{-1}) = 238.50i

C	1.51553400	0.06616700	0.08737800
C	0.65939700	1.24012200	-0.38459900
C	-0.65822600	1.24064400	0.38475000
C	-1.51542600	0.06762800	-0.08760800
C	-0.76875100	-1.29099800	-0.12731800
C	0.76747800	-1.29171000	0.12739000
H	0.44777700	1.15799100	-1.45781700
H	-1.20546500	2.17536100	0.23825600
H	-1.86608200	0.30912500	-1.09589200
H	-0.95600400	-1.74941600	-1.10018800
H	1.22311800	-1.96653700	-0.59985500
H	2.41039400	-0.02214500	-0.53306100
H	-2.41062200	-0.01978900	0.53247700
H	-1.22503600	-1.96517900	0.60012600
H	0.95424800	-1.75005800	1.10038100
H	1.86684400	0.30725700	1.09553400
H	1.20753000	2.17425900	-0.23787600
H	-0.44673500	1.15801500	1.45795600

15 (twisted boat)
Electronic Energy (Hartree) = -235.807190
LHVF (cm^{-1}) = 133.80

C	0.65521600	-1.21362400	-0.39529000
C	1.51935000	0.00050900	0.00034800
C	0.65425000	1.21438900	0.39486000
C	-0.65521700	1.21362300	-0.39529100
C	-1.51935000	-0.00050900	0.00034900
C	-0.65425000	-1.21438900	0.39486100
H	0.42019700	-1.17373800	-1.46447000
H	2.17378900	0.26911800	-0.83377200
H	1.20916700	2.14131500	0.23384200
H	-0.42019700	1.17373700	-1.46447000
H	-2.17378900	-0.26911900	-0.83377100
H	-1.20916700	-2.14131500	0.23384500
H	1.21079900	-2.14012100	-0.23481000
H	2.17225200	-0.26816600	0.83548100
H	0.41884200	1.17504700	1.46421500
H	-1.21079900	2.14012100	-0.23481200
H	-2.17225200	0.26816700	0.83548100
H	-0.41884200	-1.17504600	1.46421700

15 (boat TS)
Electronic Energy (Hartree) = -235.804968
LHVF (cm^{-1}) = 113.35i

C	0.00153000	-1.35700300	-0.43407200
C	1.26168100	-0.77484000	0.21342900
C	1.25984800	0.77766300	0.21365600
C	-0.00152200	1.35702900	-0.43395800
C	-1.26174300	0.77483400	0.21338500
C	-1.25978400	-0.77768000	0.21371400
H	0.00115300	-1.14256900	-1.50739800
H	2.14857000	-1.15441400	-0.29925800
H	2.14586800	1.15946900	-0.29887600
H	-0.00118000	1.14260400	-1.50729000
H	-2.14848100	1.15422000	-0.29970400
H	-2.14591200	-1.15952400	-0.29860300
H	0.00278100	-2.44573600	-0.33754300
H	1.32034600	-1.14369400	1.24208800
H	1.31753700	1.14642100	1.24246700
H	-0.00272600	2.44576800	-0.33742400
H	-1.32081500	1.14383100	1.24205500
H	-1.31719800	-1.14638800	1.24257400
